# Supplementary material for: Long-term mortality outcome of a primary care-based mobile health intervention for stroke management: Six-year follow-up of a cluster-randomized controlled trial
Source: PLoS Med. 2025 Mar 27;22(3):e1004564. doi: 10.1371/journal.pmed.1004564 (PMC11949329; doi:10.1371/journal.pmed.1004564)
Supplement: S1 Protocol — (DOCX) [file pmed.1004564.s001.docx]

**Long-term effect on mortality of a primary care-based mobile health intervention for stroke: six-year follow-up of a cluster-randomized controlled trial**

**Supplement 1**

**Study Protocol**

**Protocol for the SINEMA* study –**

A cluster-randomized controlled trial and long-term observational follow-up

*System-integrated technology-enabled model of care to improve the health of stroke patients in rural China

Principal investigator: Lijing L. Yan

Co-Principal investigator: Enying Gong

Approved by: Lijing L. Yan

Enying Gong

Version Dates:

May 4^th^, 2017 (Part A: main trial)

August 10^th^, 2022 (Part B: the observational follow-up)

Table of Contents

**Part A: Protocol for the SINEMA study: a cluster-randomized controlled trial**

[1. BACKGROUND 7](#_Toc169720288)

[2. RESEARCH QUESTIONS AND SPECIFIC AIMS 8](#_Toc169720289)

[3. ETHICS REVIEW AND HUMAN SUBJECT PROTECTION 8](#_Toc169720290)

[3.1 Human subject protection training 8](#_Toc169720291)

[3.2 Participant informed consent 8](#_Toc169720292)

[3.3 Safety monitoring 9](#_Toc169720293)

[4. PRE-TRIAL RESEARCH 9](#_Toc169720294)

[4.1 Contextual research 9](#_Toc169720295)

[4.2 Technological preparation 11](#_Toc169720296)

[4.2.1 Development of the SINEMA APP 11](#_Toc169720297)

[4.2.2 Development of the voice message dispatching system 16](#_Toc169720298)

[4.3 Pilot study 18](#_Toc169720299)

[4.3.1 Study population and baseline survey 18](#_Toc169720300)

[4.3.2 Pilot study procedure 18](#_Toc169720301)

[4.3.3 Evaluation and finalization 18](#_Toc169720302)

[5. STUDY DESIGN OF THE SINEMA TRIAL 19](#_Toc169720303)

[5.1 Study site 20](#_Toc169720304)

[5.2 Study population 20](#_Toc169720305)

[5.2.1 Selection of township and villages 20](#_Toc169720306)

[5.2.2 Selection of village doctors. 21](#_Toc169720307)

[5.2.3 Recruitment of participants. 21](#_Toc169720308)

[5.3 Sample size 21](#_Toc169720309)

[5.4 Randomization 21](#_Toc169720310)

[5.5 Intervention and control 23](#_Toc169720311)

[5.5.1 Provider-facing intervention 23](#_Toc169720312)

[5.5.2 Patient-facing intervention 24](#_Toc169720313)

[5.5.3 Control arm 24](#_Toc169720314)

[5.6 Data collection 24](#_Toc169720315)

[5.7 Outcome evaluation 28](#_Toc169720316)

[5.8 Process evaluation 29](#_Toc169720317)

[5.9 Economic evaluation 29](#_Toc169720318)

[6. DATA MANAGEMENT PLAN 30](#_Toc169720319)

[6.1 Data description 30](#_Toc169720320)

[6.2 Format and data standards 30](#_Toc169720321)

[6.3 Data storage, security and backup 31](#_Toc169720322)

[6.4 Access, sharing and dissemination 31](#_Toc169720323)

[7. STATISTICAL ANALYSIS PLAN 32](#_Toc169720324)

[7.1 General analysis principles 32](#_Toc169720325)

[7.1.1 Participant population. 32](#_Toc169720326)

[7.1.2 Presentation and data handling rules. 32](#_Toc169720327)

[7.2 Primary effectiveness analysis 32](#_Toc169720328)

[7.3 Secondary effectiveness analyses 33](#_Toc169720329)

[7.3.1 Secondary effectiveness analyses of continuous outcomes 34](#_Toc169720330)

[7.3.2 Secondary effectiveness analyses of binary outcomes 35](#_Toc169720331)

[7.3.2 Exploratory effectiveness analyses 35](#_Toc169720332)

[7.4 Subgroup analyses 35](#_Toc169720333)

[7.5 Strategies to handle missing data in effectiveness analyses 35](#_Toc169720334)

[7.6 Reporting 36](#_Toc169720335)

[8. QUALITY CONTROL AND PROJECT MANAGEMENT 36](#_Toc169720336)

[8.1 Project management 36](#_Toc169720337)

[8.2 Quality control for intervention 37](#_Toc169720338)

[8.3 Quality control for assessment 37](#_Toc169720339)

[8.4 Quality control for the entire study 37](#_Toc169720340)

[9. APPENDIX 38](#_Toc169720341)

[9.1 Appendix A-1. Informed consent forms 38](#_Toc169720342)

[SINEMA project consent form for stroke patients 38](#_Toc169720343)

[SINEMA project consent form for village doctors in 50 villages for survey 41](#_Toc169720344)

[SINEMA pilot research consent form for stakeholder interview 43](#_Toc169720345)

[SINEMA project consent form for verbal autopsy 45](#_Toc169720346)

[9.2 Appendix A-2: Introduction about the development of SINEMA App 46](#_Toc169720347)

[9.3 Appendix A-3. Questionnaire for participants’ survey at baseline and follow-up 54](#_Toc169720348)

[9.4 Appendix A-4. Questionnaire for Village doctors’ survey 60](#_Toc169720349)

[9.5 Appendix A-5. Population health metrics research consortium shortened verbal autopsy questionnaire 64](#_Toc169720350)

[9.6 Appendix A-6. Interview guide for process evaluation 84](#_Toc169720351)

[1. AIMS 88](#_Toc169720352)

[2. ETHICS REVIEW AND HUMAN SUBJECT PROTECTION 88](#_Toc169720353)

[2.1 Ethical review 88](#_Toc169720354)

[2.2 Participant informed consent 88](#_Toc169720355)

[3. STUDY DESIGN 89](#_Toc169720356)

[3.1 Study design, setting, and participants 89](#_Toc169720357)

[3.2 Procedures of follow-up assessment among participants 89](#_Toc169720358)

[3.2.1 Follow-up assessment for survived participants 89](#_Toc169720359)

[3.2.2 Data collection for deceased participants 89](#_Toc169720360)

[3.2.3 Strategies to improve follow-up rate 90](#_Toc169720361)

[3.3 Outcomes evaluation and measurement 90](#_Toc169720362)

[3.3.1 Primary outcomes 90](#_Toc169720363)

[3.3.2 Secondary outcomes 91](#_Toc169720364)

[3.3.3 Additional measurement in follow-up assessment 91](#_Toc169720365)

[3.4 Statistical analysis 92](#_Toc169720366)

[3.4.1 General analysis 92](#_Toc169720367)

[3.4.2 Analysis of mortality 92](#_Toc169720368)

[3.4.3 Subgroup analyses 93](#_Toc169720369)

[3.4.4 Sensitivity analyses 93](#_Toc169720370)

[3.5 Qualitative assessment 94](#_Toc169720371)

[4. QUALITY CONTROL AND PROJECT MANAGEMENT 94](#_Toc169720372)

[4.1 Pilot study 94](#_Toc169720373)

[4.2 Project Management 94](#_Toc169720374)

[5. APPENDIX 95](#_Toc169720375)

[5.1 Appendix B-1. Informed Consent Forms 95](#_Toc169720376)

[SINEMA long-term follow-up consent form for stroke patients 95](#_Toc169720377)

[SINEMA long-term follow-up consent form for village doctors 98](#_Toc169720378)

[SINEMA long-term follow-up consent form for verbal autopsy interview 102](#_Toc169720379)

[5.2 Appendix B2. Questionnaire of Patient Post-trial Follow-up 105](#_Toc169720380)

[5.3 Appendix B3. Interview outline for SINEMA long-term follow-up 126](#_Toc169720381)

[Interview guide for patients 126](#_Toc169720382)

[Interview guide for VDs, township and county managers 128](#_Toc169720383)

[REFERENCES: 131](#_Toc169720384)

**Part A: Protocol for the SINEMA study:**

**A cluster-randomised controlled trial**

**Version Date: May 4th, 2017**

# **1. BACKGROUND**

Stroke is the leading cause of deaths and disabilities in China with about 1.5-2 million new stroke cases each year and a total of about 7.5 million stroke survivors.^1,2^ The most recent estimation showed that stroke led to about 18.5% of deaths in China and was the largest contributor to the loss of disability-adjusted life years in 2016.^3^ The incidence, prevalence, and mortality of stroke in rural China have all surpassed those in urban areas in recent years.^4^ In addition, recurrent stroke constitutes a relatively high proportion of all preventable stroke in China.^2^ Among stroke survivors, the first-year recurrence rate is 11.2%, signifying the importance of secondary prevention of stroke.^5^

The main pillars of effective secondary prevention of stroke include lifestyle modification and evidence-based pharmacological treatments. These strategies have been extensively researched,^6-8^ endorsed by clinical guidelines,^9^ and proposed by WHO guidelines as cost-effective approaches for stroke patients in resource-constrained settings.^10^ However, access to even essential care is highly variable and the quality of evidence-based care is far below China’s guideline recommendations, especially in rural areas and despite the large and growing disease burden from stroke.^11-13^

There are several barriers that results into the suboptimal implementation of recommended secondary prevention of stroke in rural China. From the healthcare system’s point of view, the key barriers include fragmentation of acute-oriented care, low technical and educational capability of primary care providers, and lack of awareness and incentives for secondary prevention.^14^ Currently, there is no specific strategy to deliver follow-up services to stroke patients after acute stages in rural China. Village doctors provide primary healthcare services to the rural population under the management of township health centers; however, the quality of services is relatively poor.^15^ Although there is no solution to address all of these rural health system barriers, strengthening the primary healthcare system by capacity building to improve the quality of services have been recognized as an effective strategy for improving the prevention and control of chronic conditions.^16^

Patients’ awareness and health behaviors also contribute to the suboptimal secondary prevention of stroke in rural China. With rapid urbanization, rural communities in China are left behind with large number of older people who are vulnerable. They have relatively poor health conditions and receive limited insurance protection through the China’s New Cooperative Medical Scheme.^17^ Lacking awareness of the self-management of chronic conditions and economic difficulties result in poor adherence to secondary prevention. The PURE China study compared cardiovascular patients in urban and rural communities and found that the adherence to healthy lifestyles and the use of secondary prevention drugs are lower among cardiovascular patients in rural communities than in urban settings in all regions with different economic development levels.^18^ A study based on the China National Stroke Registry also showed that only 46.2% of the stroke patients continue the use of prescribed secondary prevention drugs after 3 months post hospital-discharge, and the persistence of use was positively associated with younger age, higher family income and the hospitals where the patients received treatment.^19^

Mobile health (mHealth) technologies, in the form of text messaging and mobile applications, have emerged as a promising approach to support behavior change among both the providers and patients.^20^ Studies showed that mHealth technologies can empower patients’ self-management of chronic conditions, increase access to healthcare services for vulnerable population, enhance communication flow, and improve the delivery of training to healthcare workers.^21-25^ However, despite the ubiquitous mobile network coverage and high penetration rate of mobile phones, few mHealth interventions have been implemented in rural China. The feasibility and effectiveness of such interventions is worthy of investigation.^26^

To improve community-based stroke care and outcomes among stroke survivors, we developed a system-integrated technology-enabled model of care (SINEMA) for the secondary prevention of stroke in rural China. The model was designed on the basis of our previous studies in rural China^27,28^ and further refined through extensive contextual research. The SINEMA model is currently being implemented and evaluated through a 1-year cluster-randomized controlled trial in fifty villages which were stratified randomized in a 1:1 ratio to either the intervention arm or the control arm in Nanhe County, China.

# **2. RESEARCH QUESTIONS AND SPECIFIC AIMS**

The **principal research question** is: Can trained village doctors and family caregivers, equipped with digital health technology, provide essential evidence-based care to stroke survivors in rural China?

The **specific aims** are:

1. To assemble and integrate each component of the SINEMA model into a holistic model of practical, scalable and seamless essential healthcare delivery for stroke patients;
2. **To evaluate the effectiveness the SINEMA model via a one-year cluster randomized controlled trial in a resource-poor rural area in China.**

# **3. ETHICS REVIEW AND HUMAN SUBJECT PROTECTION**

The project was reviewed by the Ethics Committee of Tiantan Hospital in China and Duke University in US. The pilot study has been approval by the ethics committee of Duke Kunshan University in China.

## **3.1 Human subject protection training**

All main study team members have received or will receive either online or in-person trainings on human subject protections before the study begins. All county- and township-level officers, Village doctors will receive a systematic training on human subject protections before they begin the study. The training will be organized by Duke Kunshan University.

## **3.2 Participant informed consent**

All participants will provide written informed consent. In addition to the usual individual consent, we also need to obtain the township consent. The study team will work with our local collaborators – Nanhe County CDC to introduce the study to the county department of health to seek their supports and inputs on the selection of townships for the study. The consent process in each township will be similar: first, we will hold a meeting with township stakeholders to introduce and discuss our project, followed by time for reflection and an opportunity to discuss with the research team. A final decision to participate in, or not, will be made at the second face-to-face meeting. Individual informed consent from participants, including patients, village doctors and village health promoters will be obtained before their enrollment.

#### Written informed consent from stroke patients for participating the study

A written informed consent will be obtained from participants (**Appendix A-1**). Before the recruitment, village doctors will introduce the study to all prospective participants and invite them to participate in the study. On the day of recruitment, the trained research staffs will present the information to all prospective participants. Then, a one-on-one meeting will be organized between prospective participants and research staffs in a private space. Research staffs will address questions and concerns that prospective participants have and then ask their decision. The overall consent discussion will take about 15-25 minutes. Patients will have a copy of the form with contact information.

#### Written Informed consent from village doctors for participating the study

A written informed consent will be obtained from village doctors (**Appendix A-1**). The trained researchers will conduct the consent process in a private place. The researcher will present the information on the consent document and answer questions from the village doctors. The overall process may take about 10-15 minutes. Village doctors will have a copy of the informed consent form with the contact information.

#### Written informed consent for stakeholders for participating the interview

A written informed consent will be obtained from stakeholders before the interview (**Appendix A-1**). The trained research staffs will conduct the consent process at a private place. The research staffs will present the information on the consent document and answer questions from the interviewees. The overall consent discussion may take about 10-15 minutes. Interviewees will have a copy of the informed consent form with the contact information.

#### Written informed consent of verbal autopsy for family members of deceased participants

A written informed consent will be obtained from the family member before the interview (**Appendix A-1**). The trained research staffs will contact the interviewees in advance and conduct the consent process at a private place. The overall consent discussion may take about 20-30 minutes. Interviewees will have a copy of the informed consent form with the contact information.

## **3.3 Safety monitoring**

To protect the safety of participants, we designed a multi-level support and supervision system for VDs. Each township and county hospital will have a designated physician on call to provide support to VDs on safe practice. If an event occurs, VDs have to report the event to project coordinator within 12 hours. Death and hospitalization are two main safety monitoring indexes. We will collect data through Nanhe CDC death surveillance system and the county hospital. If there are significant more death or hospitalization in the intervention group (p<0.10), we will stop the study.

# **4. PRE-TRIAL RESEARCH**

## **4.1 Contextual research**

Before the main trial, the research team visited the local communities 3 times and conducted 49 in-depth interviews with stakeholders including physicians at township and county hospitals, village doctors, stroke survivors and family caregivers. Through the contextual research, the research team identified key challenges faced by both healthcare providers and stroke survivors, and the potential solutions for addressing the barriers, which informed the design of the SINEMA model. **Table 1** shows the key results from the contextual research and the approach that the SINEMA model could be applied for addressing the barriers. The SINEMA model is designed to address the system barriers through strengthening the capacity of the village doctors through training and support, and to shift the tasks of post-acute stage management from tertiary hospital to community-based primary healthcare settings. The SINEMA model also emphasizes patients’ self-management by promoting patients’ awareness and knowledge on the secondary prevention and adherence to the preventive treatments. Thus, we expect that the SINEMA model is able to improve the access and quality of the secondary prevention of stroke among rural patients.

**Table 1. Key results of contextual research and SINEMA ways to address the barriers**

| **Barriers** | **SINEMA ways to address** |
| --- | --- |
| **Village doctors** | |
| Low adherence to clinical guidelines on the secondary prevention of stroke | Capacity building and training by specialists |
| Low health information technology and low educational capability | Use of digital health technology (SINEMA APP) |
| Lack of incentives for secondary preventive care | Providing performance-based incentives to village doctors |
| **Stroke survivors** | |
| Uncontrol of blood pressure due to poor medical adherence (Main factors related to poor medication adherence include lack of awareness, forgetfulness and economic difficulties.) | Monthly follow-up visits by village doctors  Health education on medication adherence through voice message |
| Poor physical functioning due to lack of physical activity and rehabilitation | Health education on physical activity through voice message |
| Illiteracy and health illiteracy | Use of voice message call |
| **Physicians at township and county hospitals** | |
| Fragmentation of care | Support and communication across different tiers of health facilities through training and project management |
| Over-burden of specialists | Task-sharing by primary healthcare workers |
| **Specialists at tertiary hospital in Beijing** | |
| Experienced in stroke treatment but not familiar with rural situation | Rely on the train-the trainer to train model  The training material is localized by specialists from county hospital |

## **4.2 Technological preparation**

### 4.2.1 Development of the SINEMA APP

In line with the user-centered design principles, our design framework consisted of 5 key steps, including (1) assessing the needs of relevant end users through in-depth interviews with stakeholders; (2) designing the functional modules and evidence-based contents based on end users’ needs; (3) iteratively designing and building the mHealth system structure and the app user interface based on end users’ roles and characteristics; (4) improving and enhancing the system through pilot testing and agile development based on end users ‘feedback; and (5) finalizing the mHealth system, deploying it in field trial, and evaluating its feasibility through a survey of the dominant user group (summarized in **Figure 1**) (detailed in **Appendix A-2**).


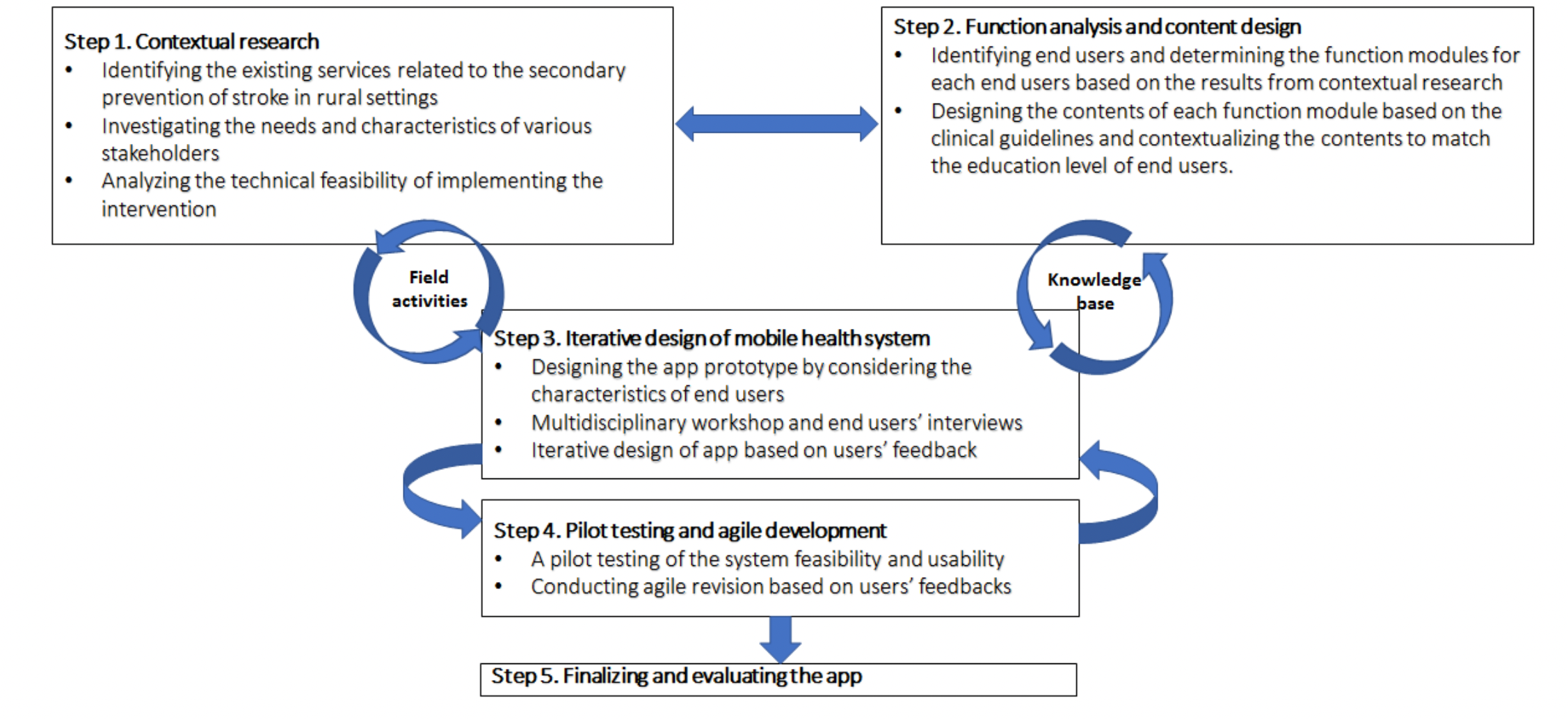


**Figure 1. Steps involved in the design of the system-integrated technology-enabled model of care mobile health system.**

To successfully complete all these steps and tasks, we assembled a multidisciplinary team of researchers who have diverse expertise in public health, medical anthropology, behavioral science, stroke treatment, care transition, software design and development, and user interface design.

#### Step 1: Contextual Research and Needs Assessment

To design an effective system that caters to the needs of both health care providers and patients on secondary prevention of stroke, we first carried out contextual research in 4 villages in Nanhe County to identify existing health care services available and accessible in the villages and assessed the needs of relevant end users.

Considering the concerns from patients and families in discussing and sharing their experience with other people in the village, and participants’ geographically dispersed locations, we adopted face-to-face in-depth interviews as the most feasible method to collect data. Participants included stroke patients, family caregivers, village doctors, and health care providers in township hospitals. Participant recruitment occurred in collaboration with local health facilities, and stroke patients and their caregivers were referred by village doctors. The number of participants interviewed was decided based on a saturation point of obtaining a comprehensive understanding where no new substantive information was being acquired. The interviews were conducted by experienced researchers. Interviews followed preprepared semi structure interview guides, which were developed after discussions among the multidisciplinary research team. Separate guides were used for different group of participants. Interviews were conducted in a private room (either in a private room in the clinics for health care providers or in the patients ‘own house) to ensure the confidentiality of the information. All interviews were audio-recorded and transcribed verbatim in Mandarin Chinese.

#### Step 2: Functional Analysis and Content Design

Based on of the results obtained from contextual research and the SINEMA intervention model design, the research team chose to design the mHealth system (**Figure 2**) in 2 parts, i.e., an app and a cloud platform that is linked with a patient-oriented message dispatch system. The research team drafted a requirement document. In the document, types of end users were identified, including village doctors, township physicians, and county managers, with the latter two also assuming management roles. The overall aims of the mHealth system—empowering primary health care providers to perform guideline-based care for stroke patients and supporting them in delivering the SINEMA intervention method—were also clearly stated.


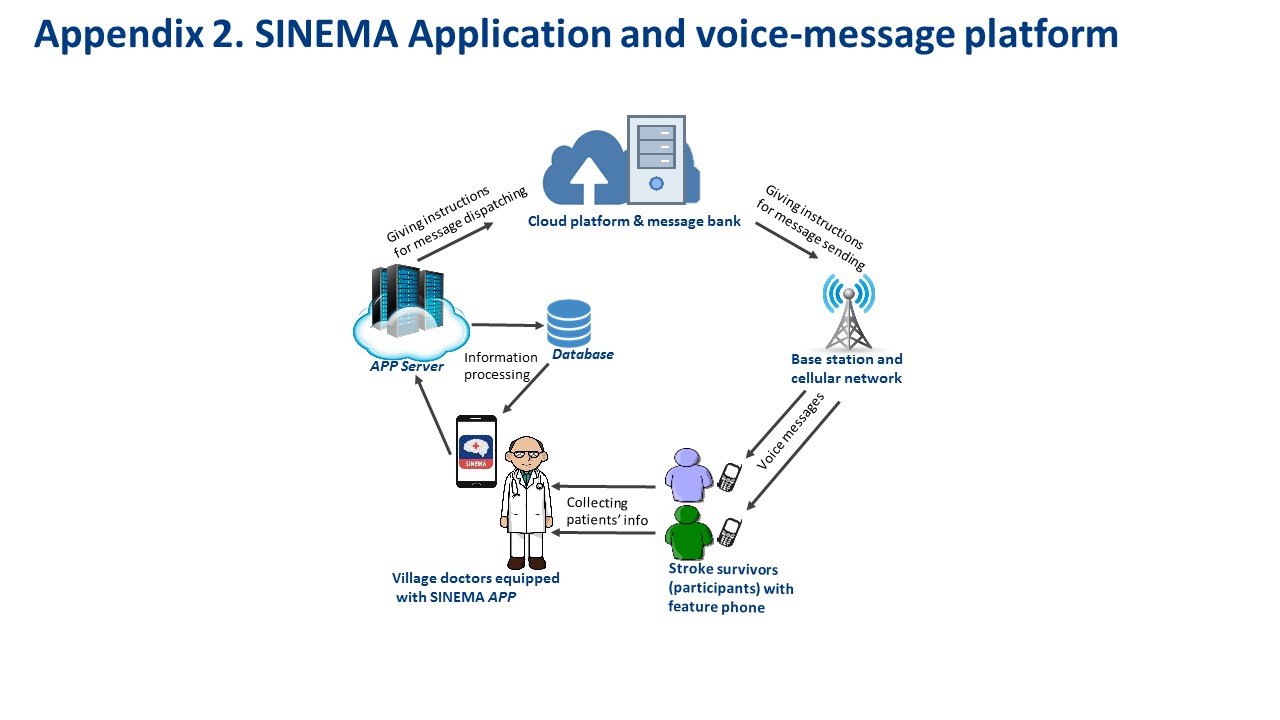


**Figure 2. A schematic diagram of the system-integrated technology-enabled model of care mobile health system.**

In this requirement document, the key functions and contents of the system modules were also drafted. The content of the modules was developed based on the Chinese clinical guideline for the secondary prevention of stroke in primary health care settings.^9^ The guideline emphasizes on early diagnosis of stroke-related symptoms and risk factor management through lifestyle modification and a combination of medical therapies. On the basis of the guideline, stroke specialists proposed the structure of the follow-up visits, taking full consideration of the different types of strokes. Then a team of researchers translated the information from the guideline into a series of questions, following a standard logic flow, for village doctors’ follow-up visit. In line with the guideline, they also prepared training materials with videos, graphs, and texts, and a list of essential medicines to assist village doctors’ decision making on medication prescription. Behavior change techniques such as goals and planning, feedback and monitoring, and social support were applied to promote the actual behavior changes in end users. The contents were further simplified and contextualized based on the principles of meeting the basic education level of village doctors and availability of medicines in the villages. A paper-based demo version of the contents on follow-up visits, training materials, and medicine lists was then reviewed by stroke specialists. Using the clinical guideline as references, stroke specialists provided their feedback on contents during a workshop meeting and verified that the contents after the simplification process were in line with the clinical guideline recommendations after the simplification process.

#### Step 3: Iterative Design of the Mobile Health System

***Design of the System Architecture***

After clarifying the functions and modules, we designed the system architecture. The whole system comprised an app client and a cloud platform (**Figure 2**). The client was a 3-in-1 app for village doctors, township physicians, and county managers, which was running on an Android mobile platform, with different user interfaces and functional modules based on the type of roles assigned. The main reasons for choosing the Android phone were that a range of affordable phone models were available and that they were very popular among village doctors, which would facilitate future scale-up of the system in rural areas. The cloud platform contained 2 parts, the app server and database server. The app server had built-in functions of security authentication, services management, and data analytics as well as communication with the third-party voice and short messaging service (SMS) text messaging gateways. The database server was deployed in a protected network (private network) inaccessible to the public network, ensuring that the database service only opens the data ports to the app server.

Medical data and personal identifiable information (PII) are sensitive data concerning a user’s privacy that need to be transmitted over the network and stored in a secure manner to avoid data loss, breach, or malicious attacks. PII data security, as one of the key issues raised by end users, was thoroughly discussed within the team and was emphasized in this system under the premise of architecture safety (**Figure 3**). In our system, to ensure the security of the data on the mobile phone, the app client did not store data;^29^ all the data were obtained through the network, and they were automatically erased when the app was closed. For the data uploading process, first, the PII data were encrypted using Advanced Encryption Standard in the app with random keys, and then the https, a secure data transmission protocol, was used to transmit the data over the network; finally, the data were stored in the database server on the cloud. This dual method of encryption offered more effective data protection. Encrypted PII data were directly stored in databases, which enhanced the level of data storage security. Similarly, for the data retrieval process in which the app client requested for data stored in the database, the cloud transmitted the encrypted PII and other data requested to the app client to be used by the end user. As the app server needed to obtain the plain text of a patient’s phone number for sending an SMS text messaging, the phone number was not encrypted on the app client but encrypted on the app server.

We designed the SINEMA mHealth system with the methods of modularization^30^ and good encapsulation to implement separate service modules and commonly used components^31^ of tools to meet the iterative design and development needs.^32^


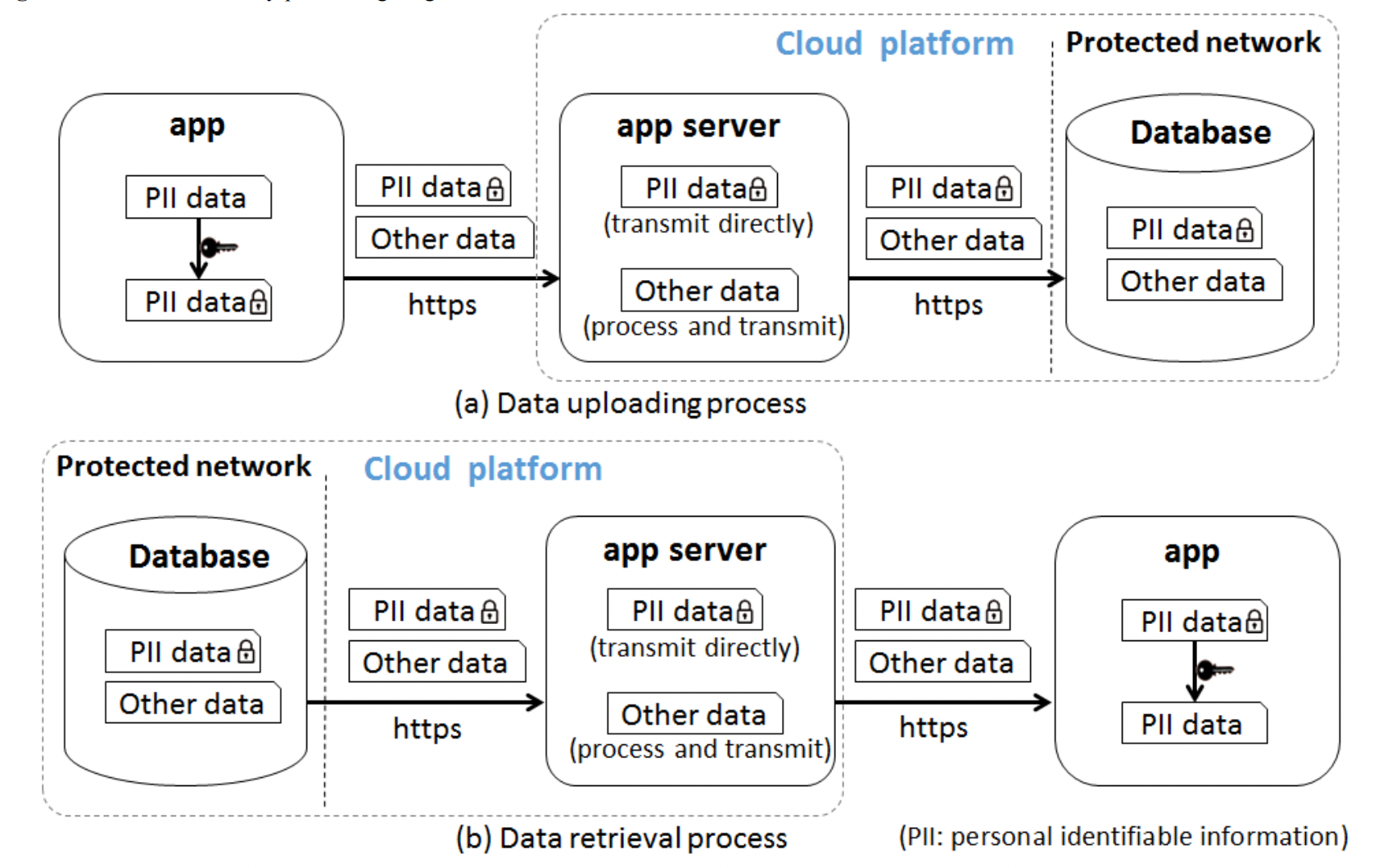


**Figure 3. Information safety processing diagram**

***User Interface and User Experience Design***

The user interface should be designed to be friendly and efficient, which is the key to improving users’ experience in utilizing and engaging with the app. Our team has designed a human-centered app by following the principle of a friendly and efficient user interface with thorough consideration of users’ needs and characteristics.^33^

First, the user interface and user experience design should reflect users’ mental models. As our target users are professionals, including doctors from village clinics and from township and county-level hospitals as well, the design of the interface took full account of their respective needs and expectations as well as limitations. In line with the results from the contextual research, we assessed the users’ proficiency in using smartphones by choosing an extensively used app as a benchmark. Considering that county and town hospital doctors are already familiar with Web-based health care information system, we adopted similar structure design and visual styles.

Second, the design should improve workflow efficiency, avoid information-entry errors, and provide smooth app access for users. Therefore, we designed simple and easily recognizable icons, such as contrasting colors for positive and negative information and distinctive graphical shapes to indicate different click status, to quickly draw users’ attention and help them understand the information effectively. In addition, we used uniform marks for navigation to make the transition and interaction of different interfaces clear and consistent. Designing with specific blocks to distinguish information contents and prefilling with defaults also improved the usability and reduced the chance of entry errors.

#### Step 4: Pilot Testing and Agile Development

Following the above studies, we then developed a fully functional app prototype, which was used for discussion in the multidisciplinary workshop as well as for demonstration to and interviews with the village doctors. On the basis of their feedback, we carried out the first round of iterative design and developed a testing version for the pilot study.

The pilot study was conducted in 4 rural villages of Nanhe County for 3 months. During the pilot study, each village doctor was provided with an Android phone with the app installed to implement the SINEMA intervention model. Each village doctor was asked to care for about 10 patients in his or her village. In-depth interviews with all the village doctors and township physicians were conducted after the pilot study to collect any feedback about the usability of the app and suggestions to optimize the app.

On the basis of the feedback from the end users, we conducted several rounds of revision of the app. During the process of development, we took advantage of the latest technologies to speed up the iteration, for example, the online tracking and positioning of bugs for rapid and efficient feedback and the hotfix technology for repairing and updating the app without releasing a new version of the app for village doctors.

#### Step 5: Main Trial and Users’ Follow-Up Survey

The finalized app system was utilized to support the implementation of the SINEMA model. **Figure 4** shows the screenshot of the key functions of the SINEMA App for three different types of stakeholders. A follow-up survey will be conducted to all users to understand their using experience of the SINEMA App.


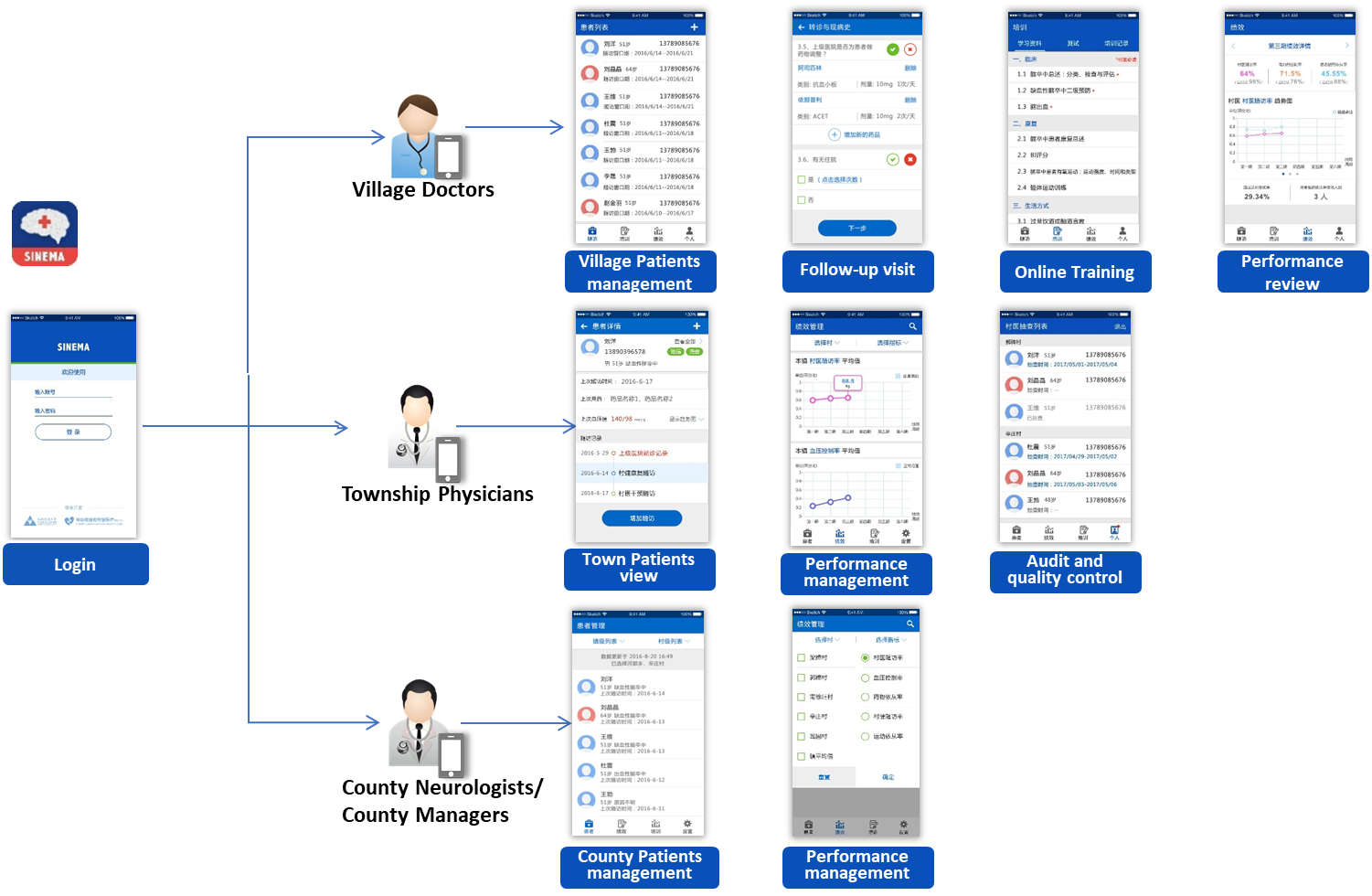


**Figure 4. Screenshot of Key Functions of the SINEMA App**

### 4.2.2 Development of the voice message dispatching system

We used multiple methodologies, including literature review, expert consultation, qualitative in-depth interviews, and field-based pilot study followed by surveys and interviews, in developing the messages to ensure that the message contents were built based on research evidence and in line with the clinical guidelines and were suitable for the local context. The development process for the mobile phone message banks consisted of the following 5 stages: (1) conducting a literature review on existing message banks targeting people with stroke, (2) interviewing stakeholders to identify the needs of stroke patients, (3) creating and designing the message contents and message sending algorithm, (4) conducting pilot testing of the messages among patients in 4 villages, and (5) refining and finalizing the message bank and sending algorithm based on the lessons learned from the pilot study. **Figure 5** outlines the design process.


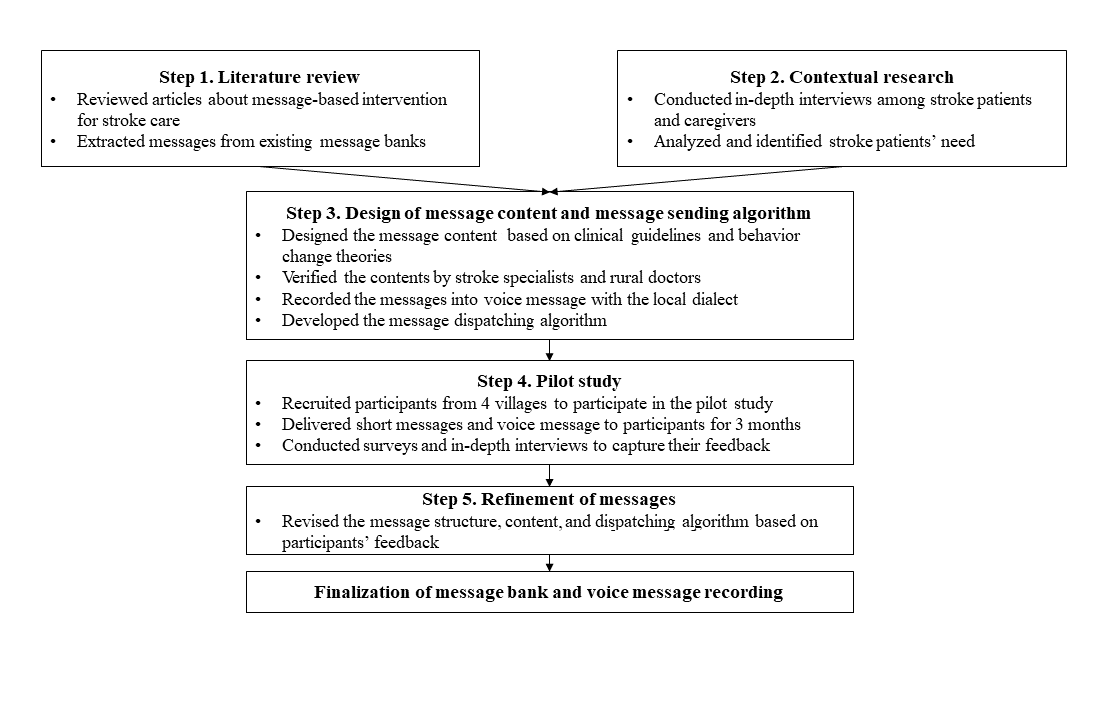


**Figure 5. Overview of key stages of message development.**

#### Stage 1. Literature Review on Existing Message Banks

To assist the design of the message bank, we conducted a literature review based on the PubMed database to search for existing text message banks published between October 1, 2011, and October 1, 2016. Our own design of the messages began in November 2016. Key search terms included stroke and text messages. A total of 2 reviewers screened the searched papers, and a snowball review by reviewing titles and abstracts of references of searched papers was also conducted to increase the scope of the review. Existing message banks were extracted and translated into Chinese as a reference for the design.

#### Stage 2. Contextual Research

To identify the needs of stroke patients, we visited the field 3 times over the period of 5 months from May to September 2016 and conducted in-depth interviews among stakeholders, including stroke patients and family caregivers. Semi-structured interview guides were developed, and questions in the interview included patients’ health status and needs for managing their conditions. Stroke patients were identified with the assistance of village doctors with the criteria that the participants were aged 18 years or older, had a history of stroke diagnosed at the county- or higher-level hospitals, were in clinically stable condition, had basic communication abilities, and were willing to participate in the study. Stroke patients’ family members who were at home while the research team visited and were willing to participate were also interviewed separately. The number of participants interviewed was determined based on the saturation theory, whereby participants were recruited until no further new information was acquired through the interview process.

#### Stage 3. Design of Message Content and Message Sending Algorithm

We designed the contents of messages on the basis of the existing message banks from the literature review, the identified needs of stroke patients from contextual research, and the behavior change theories such as the health belief model and the transtheoretical model.^34-38^ First, the structure and focus of the message bank were identified so that messages could tap into key dimensions based on patients’ needs. We then grouped the existing messages into 6 categories based on their contents, including management of metabolic risk factors, medication adherence, tobacco and alcohol control, dietary change, exercise and rehabilitation, and psychological support. The content of the messages was modified to be suitable for stroke patients in rural China. The messages were then verified by stroke specialists working in tertiary hospitals and recorded in the local dialect.

We also designed the message dispatching algorithm by setting the sending time and frequency based on the daily habits of stroke patients. This algorithm was linked with a digital health management system that was designed to support the delivery of the digital health components of SINEMA intervention.

#### Stage 4. Pilot Study

A pilot study was conducted in 4 villages to test the SINEMA intervention model, including the acceptance of the message-based intervention among stroke patients. Participants who were aged older than 18 years, had a history of stroke but in a clinically stable condition, and able to communicate via mobile phone were eligible to participate in the study. In each village, village doctors screened stroke patients in their villages and provided the list to the study team. Participants were invited by village doctors and recruited by the research team. Before the commencement of the pilot study, a structured questionnaire, including questions on participants’ demographic characteristics and disease history, designed based on previous studies,^39,40^ was administered by the research team through face-to-face interviews.

During the 3-month pilot study, participants and their caregivers received text messages at 3 pm every 2 days, and the participants also received voice messages with the same content at 7 pm on alternating days when they did not receive the text messages. At the end of the pilot study, participants completed a short questionnaire administered by their village doctors. Questions in the survey included whether they had read or listened to the messages, their understanding of the contents of the messages, and their perspectives on the helpfulness of these messages. In-depth interviews were also conducted by the research team among selected participants to seek detailed feedback.

#### Stage 5. Refinement of the Messages

After the pilot study, the research team summarized the feedback from participants and refined the message contents and message dispatching algorithm. To optimize the acceptance and understanding of the messages, the research team revised the language in each message based on participants’ feedback and preference. Village doctors and physicians from county hospitals were invited to verify the messages to ensure that local contexts were taken into full consideration, and the terms used in the messages were understandable by the target population.

## **4.3 Pilot study**

To evaluate and optimize the SINEMA model and its procedure before the main trial, a four-week pilot trial was conducted in four villages for three months.

### 4.3.1 Study population and baseline survey

The four villages were selected from two townships of the five villages for the main trial. The villages selected for the pilot trial will not be used for the main trial. In each pilot village, about 10-15 patients were recruited using the same criteria as planned for main trial. Participants also completed the baseline survey as a pilot for the survey.

### 4.3.2 Pilot study procedure

In each village, all participants recruited receive the SINEMA intervention. Village doctors who were involved in the pilot study received training and were provided with a testing version of the SINEMA App. Participants received the SINEMA intervention including monthly follow-up, daily text-messages and voice-messages and some of their family members also received text-messages. The intervention lasted for three months.

### 4.3.3 Evaluation and finalization

After the pilot intervention, four village doctors, eight patients who received intervention, two township-level managers and one county-level manager were invited to participate in an in-depth interview to provide feedback. Fifty-five participants completed a survey to provide feedback about the SINEMA intervention. The contents and delivery approach of the SINEMA intervention was optimized and finalized based on the pilot study findings before the trial.

After testing the feasibility of the SINEMA model in a 3-month pilot study conducted in 4 villages, we modified and finalized the SINEMA model to target blood pressure control, medication adherence, and physical activity. The SINEMA model (**Figure 6**), cognizant of health system’s organization around primary, secondary and tertiary healthcare levels in China, adopts the principles of cascade training with feedback and task-sharing, and relies on existing human resources available at the community level. It also proposes the use of innovative mobile technology as tools (in the form of an Android-based SINEMA APP for village doctors and cellphone voice messages for participants). The overarching aim is to strengthen the capacity of village doctors on delivering services for the secondary prevention of stroke and promoting medication adherence and physical activity among stroke survivors.


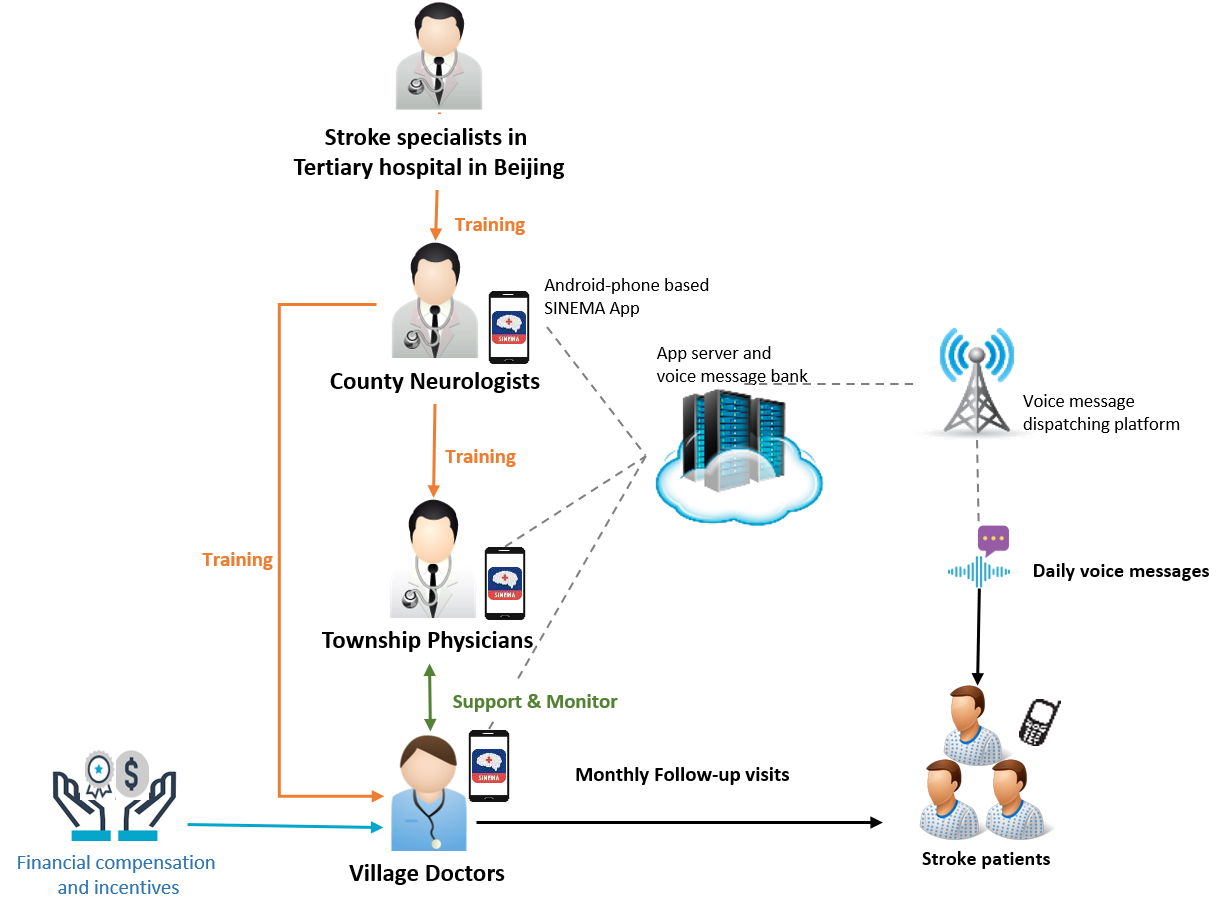


**Figure 6. Graphic figure to illustrate SINEMA intervention**

# **5. STUDY DESIGN OF THE SINEMA TRIAL**

The SINEMA trial is a cluster randomized controlled trial to evaluate the effectiveness of a system-integrated technology-enabled model of care to improve the secondary prevention of stroke in Nanhe County, a rural area of Hebei Province, China. Fifty villages from 5 townships are stratified randomized in a 1:1 ratio to either the intervention arm (implementing SINEMA model) or the control arm (usual care). The trial duration for each participant is 1 year. **Figure 7** shows the basic design of the trial flow chart.


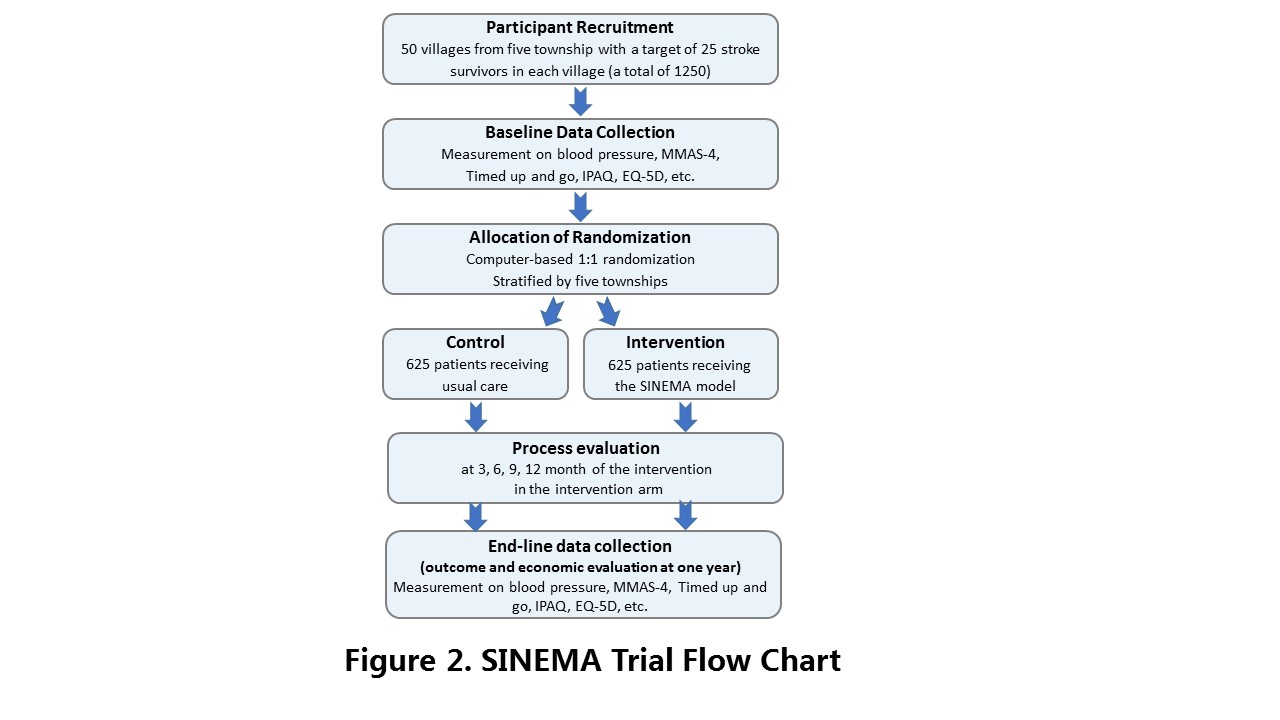


**Figure 7. The design of the SINEMA Trial**

## 5.1 Study site

This study is conducted in Nanhe County, a county in Hebei Province, China, with an intention to generate solutions that can be adapted in other resource-limited settings. Nanhe County is located on the stroke belt of China, an area marked with a comparatively high prevalence of stroke.^37^ It is a “provincial poverty county” with an annual disposable income per capita as 11,030 RMB (less than half of the average national annual disposable income per capita).^41^ In Nanhe County, there are 2 county-level hospitals, 8 township hospitals and 218 village clinics (one for each village). The capacity of these healthcare facilities is relatively limited, and the health system is fragmented across primary, secondary and tertiary healthcare facilities. Most stroke patients receive acute care in hospitals equivalent or above county level when they have urgent stroke events. Follow-up care after hospital discharge is minimal to none-existent. Access to the Internet, nevertheless, is widespread, and cell phone (including both feature phone and smartphone) ownership among adults is high (>90%).^42^ A survey conducted among rural residents in Hebei Province found that 94.7% participants owning a mobile phone and about half of them surf the internet through the mobile phone.^43^

## 5.2 Study population

### 5.2.1 Selection of township and villages

We will select 5 out of the 8 townships of Nanhe County based on their demographic and economic conditions, and township leaders’ willingness to participate in our study. The top 5 townships where there are the greatest number of the villages with a minimum population size of 1500 were selected to participate in the study. In each township, we will select 12 villages from each township as eligible villages where the number of population ranks at the top with a minimum population size of 1500. In each village, we will ask village doctors to screen the stroke survivors in their villages based on health records and their best knowledge and generate a list of potential participants. To reach the targeted sample size, we will recruited 10 villages from each township according to the number of stroke survivors screened and village doctors’ willing to participate in the study.

### 5.2.2 Selection of village doctors.

For each village, 1 village doctor will be selected who is an officially certified village doctor and can stay in the village for at least 4 days per week in the next 12 months, as well as knows how to use a smartphone and agrees to participate in the study. If there is more than 1 eligible village doctor in the village, the leader of the village clinics will be selected.

### 5.2.3 Recruitment of participants.

Stroke survivors meeting the following criteria will be recruited to form the study sample: those who are over 18 years old, have a history of stroke (including ischemic and hemorrhagic stroke) diagnosed at county hospital or higher-level facilities, are currently in a clinically stable condition and not receiving acute stroke treatment, will live in this village for at least 9 months during the next 12 months, have basic communication ability (i.e. can understand simple instructions) and give their informed consent. Exclusion criteria are: patients who are unable to get out of bed without maximum assistance, have serious life-threatening disease such as cancers or an expected life span of less than 6 months.

The recruitment will be conducted village by village. Eligible stroke patients will be invited to participate in the study by village doctors. If there are less than 30 screened stroke survivors in the village, all screened stroke survivors were invited. If there are more than 30 screened stroke survivors, 30 stroke survivors are randomly selected and invited by village doctors. Recruitment is conducted by trained research staffs in the village clinics. All patients will be informed and provided written consents before participating in the study.

## 5.3 Sample size

Based on a sample of 1,250 prevalent stroke patients in 50 villages (with 25 villages per arm and on average 25 patients per village), assuming loss of 2 clusters (villages) per arm and loss of 1 patient per village on average, an intra-cluster correlation coefficient (ICC) of 0.04 (a conservative assumption), and a standard deviation (SD) for SBP pre-post change of 20 mmHg, the study has 83% power (with 2-sided alpha = 0.05) to detect a 5 mmHg net difference in pre-post change of SBP between intervention and usual care arms. Previous studies have been demonstrated that a 5mmHg difference is a meaningful change for stroke recurrence in 3-5 years.^44^ The effect size of a 5mmHg difference corresponds to a standardized effect size of 0.25 (5/20) and therefore we will have more than 80% power to detect such standardized effect sizes for secondary outcomes of interest, assuming the same level of clustering of those outcomes.

## 5.4 Randomization

The purpose of using a cluster randomized design is to reduce the contamination and improve the practical feasibility for implementing the intervention protocol.^45,46^ The 50 villages will be randomized in a 1:1 allocation ratio to the intervention or control arm with stratification by the 5 townships. Randomization will be conducted by an independent biostatistician at Duke University who was not part of the study. Within each township (strata), a uniform random number between 0 and 1 was generated for each of the 10 villages, the numbers will be sorted in increasing order and then the first 5 and second 5 villages were labeled “A” and “B”, respectively. Finally, a virtual coin flip was performed to assign one of “A” and “B” to intervention and the other to usual care.

**
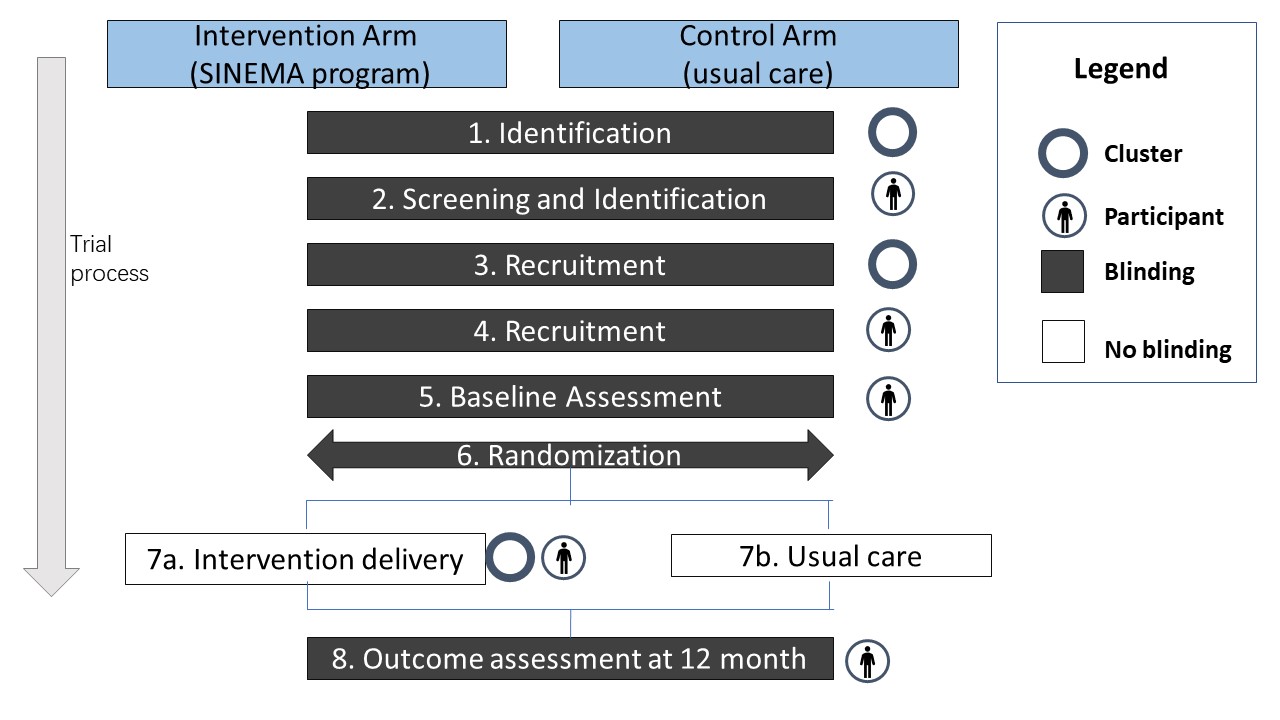
**The randomization will be conducted independently of the participants’ recruitment and baseline survey. Both research staffs and village doctors who will be involved in the recruitment and baseline survey will be blinded of the randomization. The allocation of the intervention and control arm will be released to village doctors and the implementation team in each township after all study participants in the township had been enrolled and completed the baseline survey. The **Figure 7** showed the Timeline cluster diagram^47^ of the trial.

**Figure 7. Timeline cluster diagram for the SINEMA cluster trial**

Notes:

Cluster identification: The study team identified five townships out of a total of eight in Nanhe County with the greatest number of villages with a minimum population size of 1500. Potential eligible villages (12 villages per township) were identified based on the village population size and village doctors’ willingness to participate. A total of 60 villages participated in the screening phase.

1. Participant identification: Village doctors screened stroke patients based on existing health records and door-to door visit to find out residents who have ever been diagnosed with stroke. Village doctors provided a list of potential participants to the research team.
2. Cluster recruitment: Our target cluster size is 50 villages. To reach to the sample size and get a balanced cluster size, the research team selected ten villages with the highest number of screened stroke survivors out of twelve eligible villages in each township. The research team visited selected villages expressing their intention to participate and provided an oral explanation of the study in detail. Written consent is obtained from village doctors.
3. Participant recruitment: If the total number of screened stroke survivors in the village was less than 30, the research team asked the village doctor to invite all patients on the list to participate in the study. If the total number of screened stroke survivors in the village was larger than 30, the research team randomly selected 30 participants to invite to take part in the study. The recruitment was conducted in the village clinics by trained research staff who were blinded of group allocation status.
4. Participant baseline assessment: Performed by the trained research staff who were blinded of the study design during the face-to-face interview after consent was obtained. Standard anthropometric indicators including blood pressure, height, weight, waist, timed-up and go test were measured by trained blinded study personnel.
5. Randomization: The randomization was performed by an independent statistician from Duke University with blinding to the identity of the cluster and village doctors. The 50 villages were randomized in a 1:1 allocation ratio to the intervention or control arm with stratification by the five townships. The results of the randomization were blinded among all research staffs who participate in the recruitment and baseline assessment. The allocation of the randomization in a township were informed by a message from the Principle Investigator to the village doctor and local project manager once participants in a township were all recruited and completed the baseline assessment.

7a. Intervention delivery: Village doctors and stroke survivors received the SINEMA intervention. No blinding for village doctors and patients was possible.

7b. Patients received usual care. No blinding for village doctors and patients was possible.

8. Participant outcome assessment at 12 months: Data were collected by independent blinded trained research staff (same group of people with the baseline assessment). Face-to-face interview and standard anthropometric measurements were performed.

## 5.5 Intervention and control

The intervention arm will implement the SINEMA model for 1 year, which consists of a provider-facing intervention aiming to strengthen the capacity of village doctors in delivering stroke secondary prevention, and a stroke survivor-facing intervention aiming to promote medication adherence and physical activity.

### 5.5.1 Provider-facing intervention

#### Systematic cascade training for village doctors:

Systematic training will be provided based on “train the trainer to train” model. All the training materials are drafted based on the clinical guidelines^9^ by experienced stroke specialists from tertiary hospitals with adjustments to suit the knowledge level and learning abilities of village doctors. Training consists of an initial 1-day training session on stroke clinical guidelines, essential medications, strategies for using Health Belief Model to promote medication adherence and physical activity, and the intervention protocol. Only village doctors who pass the test after the initial training can take part in the intervention. A 3-hour refresher training session will be provided at the 3 months after the intervention begins.

#### Monthly follow-up visits with the support of the SINEMA APP:

Village doctors in the intervention arm are provided with the SINEMA APP, designed for this study to standardize the flow of the monthly follow-up visit. With the assistance of SINEMA APP, during each monthly follow-up visit, village doctors will collect information about participants’ health conditions, medication use, blood pressure level, etc.; and provide health education to participants on medication adherence and physical activity.

#### Village doctor group activities:

Every week, a village doctor group activity will be organized through WeChat, an extremely popular smartphone-based messaging and social media APP in China. During the group activity, village doctors share their experience in follow-up visits and project implementation, report concerns to the research team, and seek support from the research team or their peers.

#### Performance feedback and incentives:

An appropriate amount of payment is offered to village doctors determined by the number of follow-up visits conducted. In addition, an incentivizing bonus for good performance and quality will be offered. The factors considered include the quality of data that village doctors collected through APP, number and quality of responses in WeChat, results of the on-site supervision by the research team, etc.

### 5.5.2 Patient-facing intervention

To improve the medication adherence and physical activity of stroke survivors, our stroke survivor-facing intervention program includes the following components:

#### Briefing session:

A briefing session was organized in each village in the intervention arm. During the session, the research team introduced the SINEMA project, provided a brief health education session on the importance of blood pressure control, medication adherence and physical activity.

#### Monthly follow-up visits and follow-up handout:

Every month, participants will be visited by their village doctors who are equipped with SINEMA APP with a standard flow of follow-up visit. During the follow-up visit, participants will be educated about their blood pressure level, the importance of medication adherence and physical activity. Participants will also be provided with a handout designed specifically for stroke survivors with low literacy showing their blood pressure level, their medication prescription and their goal of physical activity as a tool for them to adhere to doctors’ prescription.

#### Voice messages for health education:

Participants in the intervention arm will receive 1 voice message at 7 AM every day. The message contains information to remind them to take their prescribed medication and be physically active. A message bank of 365 messages was designed by research team, verified by stroke specialists and rural doctors based on the Health Belief Model, modified to suit the goals and context of stroke survivors in rural China.

### 5.5.3 Control arm

Villages in the control arm will continue their usual practice without the introduction of any of the SINEMA activities described above. For the villages in the control arm, there is no specific healthcare service focusing on stroke patients. Village doctors provide both clinical services (including but not limited to blood pressure tests and medicine prescription based on the needs of patients when patients walked in the clinic) and basic public health services (including 4 follow-up visits per year among people with hypertension and diabetes as required by the government).^48^

## 5.6 Data collection

We will invite participants in both the intervention and control arms to visit the village clinics to receive a comprehensive survey and standard anthropometric measures at baseline and the end of the study (12 months). The data collection will be conducted following a standard protocol by trained staff recruited from the Center of Disease Control and Prevention (CDC) of a nearby county, who are completely blinded of randomized allocation of villages and will not be involved in the implementation of the intervention. For patients, a brief interviewer-administered questionnaire (**Appendix A-3**) will be completed using Qualtrics, an online-survey platform, which is able to ensure the quality of data through setting the format and force response. The questionnaire will collect data on demographic information, disease history, medication use and adherence, lifestyle behaviors, and utilization of healthcare services including hospitalization. Patients will have a physical examination comprising of assessment of blood pressure, weight, height, waist circumference and the "Timed Up and Go" test.

Village doctors will be invited to complete a self-administered knowledge-attitude-practices questionnaire (**Appendix A-4)** including questions regarding their demographic characteristics and clinical practice experience. The survey consists of 6 questions regarding their knowledge on the secondary prevention of stroke guidelines and 17 questions regarding their attitude and practice in delivering the stroke secondary prevention to patients evaluated by using 5-Likert scale.

Data on hospitalization and recurrence of stroke will be collected through extracting the data from county medical insurance system and death record system. Data on hospitalization will be extracted every month from the medical insurance system, which includes the reason and duration of the hospitalization. Death data will be extracted through the death report from the local CDC and verified through a smartphone-based shortened version of verbal autopsy survey administered by county project officer to family members of dead participants in this study. The survey has been proved to be feasible and acceptable in rural north China.^49^ Respecting the culturally appropriate grieving period, the interview will be conducted at the sixth and twelfth month after initial of the intervention. **Table 2** shows the overall data collection procedures based on SPIRIT guideline.

**Table 2. The schedule of enrollment, intervention and assessments**

|  | **STUDY PERIOD** | | | | | | | | |
| --- | --- | --- | --- | --- | --- | --- | --- | --- | --- |
|  | **Enrollment** | **Baseline Assessment** | **Allocation of treatment arms** | **Post-allocation** | | | | | **Close-out**  **Assessment** |
| **\TIMEPOINT** | ***-3 mon*** | **-2 mon** | **0 mon** | ***0 mon*** | ***3 mon*** | ***6 mon*** | ***9mon*** | ***12mon*** | ***13 mon*** |
| **ENROLMENT:** |  |  |  |  |  |  |  |  |  |
| **Eligibility screen and recruitment** | X |  |  |  |  |  |  |  |  |
| **Informed consent** | X |  |  |  |  |  |  |  |  |
| **Randomization *** | X |  |  |  |  |  |  |  |  |
| **Allocation of treatment arms** |  |  | X |  |  |  |  |  |  |
| **INTERVENTIONS:** | | | | | | | | | |
| **Systematic Cascade training for village doctors** |  |  |  | X | X |  |  |  |  |
| **Village doctors’ Monthly follow-up visits to patients** |  |  |  |  |  |  |  |  |  |
| **Village doctor group activities** |  |  |  |  |  |  |  |  |  |
| **Performance feedback and incentives for village doctors** |  |  |  |  |  |  |  |  |  |
| **Briefing session for participants** |  |  |  | X |  |  |  |  |  |
| **Daily voice messages for participants** |  |  |  |  |  |  |  |  |  |
| **OUTCOME ASSESSMENTS:** | | | | | | | | | |
| ***Systolic blood pressure*** |  | X |  |  |  |  |  |  | X |
| ***Mobility*** |  | X |  |  |  |  |  |  | X |
| ***Medication adherence*** |  | X |  |  |  |  |  |  | X |
| ***Physical activity level*** |  | X |  |  |  |  |  |  | X |
| ***Quality of life*** |  | X |  |  |  |  |  |  | X |
| ***Stroke recurrence*** |  | X |  |  |  |  |  |  | X |
| ***Hospitalization*** |  | X |  |  |  |  |  |  | X |
| ***Morbidity and mortality*** |  |  |  |  |  | X |  |  | X |
| ***Village doctors’ knowledge, attitude and practice on the secondary prevention of stroke*** |  | X |  |  |  |  |  |  | X |
| **PROCESS AND ECONOMIC ASSESSMENTS:** | | | | | | | | | |
| **Process evaluation** |  |  |  |  | X | X | X |  | X |
| ***Cost of intervention*** |  |  |  |  |  |  |  |  | X |

## 5.7 Outcome evaluation

The primary outcome is systolic blood pressure (SBP) of participants. The effectiveness of the program will be evaluated by the SBP at 1-year follow-up between the intervention and the control arm. Blood pressure is measured on the right upper arm (or left arm if the right arm is disabled) with participant seated after 5 minutes of rest using an electronic blood pressure monitor (Omron HEM-7052). Two measurements are taken, and the mean value is calculated. If the differences between the 2 SBP is larger than 10 mmHg, a third measurement is conducted, and the mean value of the only or the last 2 reading is calculated.

Secondary outcomes include participants’ diastolic blood pressure, mobility, medication adherence, physical activity level and quality of life. Mobility is measured using the Timed Up and Go test, a simple and quick functional mobility test that requires the participants to stand up, walk 3 meters, turn, walk back, and sit down.^50,51^ Medication adherence is measured using the 4item Morisky Green Levine Scale with scores ranging from 0 to 4 with a higher score indicating lower medication adherence.^52^ Participants will respond to the 4 questions with yes or no for each type of medicines (antihypertension, aspirin and statin) if they were prescribed by the physicians before.^52^ Physical activity level is measured using the short version of the International Physical Activity Questionnaire (IPAQ).^53^ Participants were asked to recall the frequency and duration of rigorous activities, moderate activities and walking they performed in the past 7 days. Quality of life is measured using EuroQol- 5 Dimensions-5L (EQ5D).^54^

Exploratory outcomes include stroke recurrence, hospitalization, disability and mortality, which will be collected through questionnaire or medical insurance records. (See **Table 3**) In addition, based on the mortality data we collect, we will conduct a smartphone-based shortened version of verbal autopsy (VA) survey to family members of dead participants in this study, to improve the quality of cause of death data. This survey has been proved to be feasible and acceptable in rural north China by our study team 1.^49^ We will use the Population Health Metrics Consortium (PHMRC) Shortened Questionnaire adult module (see **Appendix A-5,** bilingual and adjusted for SINEMA study), which has been validated and elicits sufficient information to diagnose most probable cause of death of public health importance. Trained health workers will conduct the VA interview, which will take about 30 minutes. Respecting the culturally appropriate grieving period, the VA interview will be conducted within 4-12 months after the date of death. An appropriate respondent (usually the family member) must be identified, and informed consent (see **Appendix A-1**) must be obtained before the interview. The trained VA interviewer will conduct the interviews in the local language.

**Table 3. Outcome variables in baseline and follow-up survey**

| **Outcomes** | **Measures** | **Methods** |
| --- | --- | --- |
| **Outcomes for participants:** | |  |
| Primary outcome: | Systolic Blood Pressure | Electronic BP monitor* |
| Secondary outcome: | Diastolic Blood Pressure | Electronic BP monitor* |
|  | Mobility | Timed Up and Go test ^55,56^* |
|  | Medication adherence | Questionnaire: MMAS-4^57^* |
|  | Physical activity level | Questionnaire: IPAQ short form^58^* |
|  | Quality of life | Questionnaire: EQ5D ^54^* |
| Exploratory outcomes: | Stroke recurrence | Questionnaire and medical insurance records** |
|  | Stroke related morbidity | Questionnaire and medical insurance records** |
|  | Stroke related mortality | Questionnaire, death records and verbal autopsy *** |
| **Outcome for village doctors:** | Knowledge, attitude and practice of village doctors | Questionnaire self-administered by village doctors |

⁎ The outcomes for participants will be measured by trained staff recruited from Center of Disease Prevention and Control of a nearby county, who are complete blinded of randomized allocation of villages and will not be involved in the implementation of the intervention.

⁎⁎ Data will be collected through extracting information from medical insurance records.

⁎⁎⁎ Data on mortality will be collected through extracting information from death records and verified by a smartphone-based shortened version of verbal autopsy survey.

## 5.8 Process evaluation

Four waves of process evaluation will be conducted in the intervention arm at the 3rd, 6th, 9thmonth from the initial intervention and the last one after the 1-year intervention to document how the SINEMA intervention is implemented and to explore the essential components of the intervention. Face-to-face in-depth interviews with key stakeholders involved in the study including patients, village doctors, doctors at township level, study coordinators, county government officials will be conducted by independent investigators. A semi-structure interview guide will be developed (**Appendix A-6**), and all interviews will be recorded, transcribed and analyzed before the beginning of the follow-up survey, to avoid prejudice in interpretation. We will also try to reach village doctors if participants from their village discontinue from the intervention to understand the factors related to their lost to follow-up.

## 5.9 Economic evaluation

The economic evaluation will be conducted. We will consider costs from the perspective of health services instead of the societal perspective because the aim of the study focuses on how SINEMA model could solve healthcare related issues and stroke survivors are already being in the community with very few of them have official and stable work poststroke. Thus, pension costs and worker productivity are not as relevant to our study design. Data on costs of the intervention such as study design, APP development and incentives for the village doctors, will be collected from project financial reports. Data on the cost related to utilization of health care services and medications will be collected at the 1-year follow-up surveys. This information will be supplemented by insurance claims data focusing on both inpatient and outpatient costs. For the trial-based evaluation, cost effectiveness will mainly be assessed using 2 indicators: cost per unit reduction in systolic blood pressure and cost per unit increase in quality adjusted life year (QALY). If suitable, we will conduct analysis for long-term cost-effectiveness by considering the situation beyond the trial, a decision-analytic model will be developed to enable long-term outcomes to be simulated, which will draw on the literature and available databases. Sensitive analysis will be conducted to determine the robustness of the estimates.

# **6. DATA MANAGEMENT PLAN**

## 6.1 Data description

The following database will be generated and managed by the research team with supervision from the international steering committee.

#### Data from patients’ screening

During the patients’ screening, village doctors will collect basic information about all stroke patients in their village. Data includes name, gender, age, phone number and stroke history. These information will be mainly used for patients’ recruitment and no information will be used for data analysis.

#### Intervention-related data

Intervention related data will be collected through SINEMA APP by Village doctors and VPs. During each patients’ follow-up, Village doctors will collect data about patients’ blood pressure, recent hospital visits, and medication use. VPs will collect data about patients’ Bathel Index and physical activity. These information will be used mainly for project management and process evaluation. Some data could be used as a supplementary database for the evaluation.

#### Data for outcome evaluation

Evaluation-related data includes assessments of patients on the primary and secondary outcomes of the trial, including systolic blood pressure, stroke occurrence, disability and dependence, medication adherence, health status, activities of daily living, mobility, and risky lifestyles.

#### Data for process evaluation

Data collected from the process evaluation are audio recordings of the interviews and focus group analysis conducted with the patients, their caregivers, village doctors and health promoters. The topics of these interviews include the effectiveness of the intervention, and feedback and comments on the intervention and the use of the SINEMA APP.

#### Data for economic evaluation

Before the baseline data collection, personal ID number and social security number will be collected by project manager or county manager to support the economic evaluation. Data for the economic evaluation includes costs of the intervention, and patients’ medical cost and quality of life. This information will be supplemented by insurance claims data.

## 6.2 Format and data standards

Data will be collected and stored in formats generally accepted within the public health community. Intervention related data will be collected through the App and download from the server for analysis. Evaluation data will be collected through *Qualtrics*, an electronic data collection system. Qualitative data will be captured in MPEG-1 Audio Layer 3 (.mp3) format. Individual interviews and focus group interviews will be transcribed into text files using *MS* *Word* (docx, or .doc). This qualitative data, interview transcripts, and any field notes will be imported into and analyzed using *QSR Nvivo*.

## 6.3 Data storage, security and backup

All data associated with this project will be maintained in strict compliance with both international and local policies. All associated digital research data will be physically stored on an encrypted secure server maintained by the research team using standard file formats. No data will reside on portable or laptop devices. The digital files with identifiers and recordings will be stored on a password-protected hard drive that has been encrypted. Paper files and the password-protected hard drive will be kept in a locked storage cabinet and the key protected by the P.I., only giving authorized personnel access to the key.

The researchers in Duke Kunshan University are responsible for storage, maintenance and back‐up of the data based on the data security level. Network‐stored research data is backed up on a daily basis to a separate password‐protected secure server maintained by Duke Kunshan University.

#### Data management, curation and storage

Approaches include: (i) curation in SAS following a normalized relational database design; (ii) labelling, formatting, documenting, and deriving variables in a consistent manner over time and across samples; (iii) an established file management strategy (folder structure and file naming); (iv) secure storage, backup and access to the IT department of the Institute; (v) multiple layers redundancy, eg: raw data will be stored in duplicate on password-protected external hard drives kept in separate secure locations; and (vi) use of flexible formats to facilitate analyses in statistics packages.

#### Storage of personal data

Personal identifiable data (including name, ID and phone number of the participants) will only be used when essential for generating list of participants for follow-up, matching with the health information system and for supporting message dispatching as part of the intervention. This information will be stored separately from other collected data and only accessed by the study PI.

To protect the privacy and confidentiality of the identifiable information, we will separate the data collection of identifiable information from other data. After the data are collected, entered and digitized, all personal identifiable information including the ID will be removed from the final data file for data analysis. All devices used to access and process data include password-protected desktop computers. Data are stored on the secure institutional server protected by firewalls. No remote access is employed.

In addition, no personal data will be linked with the response at any time during the study. The data analysis and report writing will be conducted based on an aggregated format.

## 6.4 Access, sharing and dissemination

The P.I. will be responsible for ensuring that all project members are aware as to the ownership of data and who may access them and under what conditions. Online access to the data will be password protected. All research groups have been trained in human subject protection and only trained project staff operating under the IRB approval for the project will have access to the confidential individually identifiable data. After the data are collected, entered, and digitized, the identifiable information will be stored in a separate dataset that will be password protected. All data will be aggregated or de-identified for publication and baseline and follow-up surveys will be linked by study ID only. Paper files will be kept in a locked cabinet and access to the key will be limited to the P.I.

Findings from the data and the study will be disseminated through scientific presentations at national, regional or international conferences; or published in international scientific journals. Summaries may also be disseminated to the collaborators involved in the study.

# **7. STATISTICAL ANALYSIS PLAN**

## 7.1 General analysis principles

### 7.1.1 Participant population.

Patient-level outcome data will be used, and stratification and clustering will be taken into account in all analyses. The analysis population will consist of all enrolled participants who completed the baseline assessment. Missing follow-up measurements due to (1) death, (2) moving away from the study area, or (3) refusing to participate in the follow-up assessment will be accounted for in the analyses.

The analysis will use the intention-to-treat principle whereby all participant-level outcome data will be analyzed according to the arm to which the patient was allocated. Given the challenges of defining protocol violation in the context of a complex multi-component intervention like SINEMA, we will not perform per-protocol analyses but will instead summarize adherence to intervention components among intervention-arm participants and will summarize the frequency of visits to the village clinic in the control arm.

### 7.1.2 Presentation and data handling rules.

Continuous variables will be reported as mean and standard deviation, if symmetric, or as median with 25th and 75th percentile, if not. Counts and proportions will be reported for categorical variables. Outliers will be identified through boxplots and histograms and will be queried at the data checking stage if an error is suspected. Ninety-five percent confidence intervals will be presented, and significance of the primary outcome will be assessed based on a test at the two-sided 5% significance level.

## 7.2 Primary effectiveness analysis

The mixed effects framework^59,60^ will be used to model the primary outcome of short-term and long-term change in SBP with a random intercept for cluster (to account for the cluster randomized design) and fixed effects for township (to account for the stratified randomization procedure).^61^ More specifically, the primary outcome will be regressed on an indicator for treatment arm, baseline SBP, gender and age—in order to gain power—and indicator variables for township. Restricted maximum likelihood will be used together with the between–within method to calculate test degrees of freedom.^59^ Both methods have demonstrated good performance for binary outcomes and this good performance is expected for continuous outcomes, particularly when there are fewer than 40 clusters. For completeness, between-arm differences will also be presented based on the same model but with no adjustment for age and gender.

Baseline characteristics will be cross tabulated according to the randomized arm to visually check for appropriate balance and to provide an overview of the study population. If substantial baseline imbalance between the two arms is identified and the relevant variable is beyond the list of pre-specified variables for adjustment, additional sensitivity analyses adjusted for these imbalanced variables will be performed to ensure the robustness of the analysis.

## 7.3 Secondary effectiveness analyses

As indicated in **Table 4**, we plan to analyze both binary and continuous secondary outcome variables. Here we describe key elements of the analytical approaches and of any anticipated challenges to modelling these outcomes.

**Table 4. Definitions and Specifications of Outcome Variables**

| **Outcome variables** | **Definitions** | **Specification for analysis** |
| --- | --- | --- |
| **Primary outcome** | | |
| Systolic blood pressure | Blood pressure is measured using a standardized electronic monitor (Omron HEM-7052) with individual in a seated position. Two reading are taken. If the differences between the two SBP readings is larger than 10mmHg, a third is taken. The mean of the only two SBP, or of the last two readings, will be used as the final measurement. | Continuous |
| **Secondary outcomes** | | |
| Diastolic blood pressure | As for SBP, the DBP outcome will be the mean of the only two, or of the last two readings of DBP. | Continuous |
| Mobility | Mobility is measured using the “Timed Up and Go” test, a functional mobility test that requires the participants to stand up, walk 3 meters, turn, walk back and sit down. The mobility outcome will be specifically measured by the total time taken to complete the test, for which faster times (i.e. lower values) represent better functional mobility status. For participants who cannot complete the test (e.g. who cannot stand up), we will use a range of strategies (i.e. set to missing, set at maximum observed time or at some larger maximal tie) to deal with this issue and will evaluate how sensitive the estimated intervention effect is to those assumptions. The variable will also be dichotomized at a cut-off of 14s based on previous studies. | Continuous as total time  to complete the test; and  binary using cutoff of 14s |
| Physical activity | Physical activity is measured with the short version of the International Physical Activity Questionnaire. Total physical activity score (MET-minutes/week) and activity classification (inactive, minimally active and health enhancing physical activity-HEPA) are derived according to the IPAQ scoring guideline. | Continuous as total  physical activity score;  and binary using cutoff of  HEPA active vs. HEPA  inactive. |
| Quality of life | Quality of life is measured with EuroQol-5 Dimensions-5L (EQ5D), which consist of five dimensions and each with five levels of perceived problems. These dimensions are combined to give a five-digit code and a single index-based numerical value (ranged from 0 to 1) will be derived based on the value set for Chinese population. | Continuous as utility |
| Medication use | Medication use is self-reported for each of three dinstinct medication types: anti-hypertensive medicine, aspirin and statin. | Binary yes/no for each of  three medication types. |
| Medication adherence | Self-reported medical adherence is measured by Morisky Green Levine Scale separately for anti-hypertensive medicine, aspirin and statins if the participate was taking the medicine. A score ranged from 0 to 4 are calculated by summing all coded answers where lower scores correspond to better medication adherence. | The variable will be dichotomized as high adherence (0) vs. low to medium adherence (1-4). |
| **Exploratory outcomes** | | |
| Stroke recurrence | Participants self-reported whether they had experienced stroke events during the 12-months follow-up period. | Binary as experienced a stroke event vs. not experienced a stroke event |
| Degree of Disability  (Modified Rankin Scale) | Participants’ degree of disability evaluated based on assessors’ observation and participants’ self-reported symptoms or disability. A score ranged from 0 (no symptoms) to 5 (severe disability). A score of ≤2 indicates functional independence. | Binary as score 0-1 (no symptoms at all or no significant disability despite symptom) vs (moderate to severe disability) |
| Stroke-related hospitalization | Participants self-reported whether they were admitted in the hospital due to stroke event. | Binary as hospitalized due to stroke vs. not hospitalized due to stroke |
| Mortality | Report on whether the participant died during the 12-months follow-up period | Binary as diseased vs alive |

### 7.3.1 Secondary effectiveness analyses of continuous outcomes

Secondary outcome variables that are continuous (i.e. DBP, mobility, physical activity and quality of life) will be analyzed using the same mixed effects modeling approach used for the primary outcome of change in SBP because all of the variables are measured at both baseline and one-year follow-up. The three secondary variables of mobility, physical activity and quality of life have lower bounds of 0 and are anticipated to be right-skewed, so that analysis of those variables may not satisfy assumptions of the mixed effects modeling approach. Fortunately, because we plan to model changes in these variables, we anticipate that assumptions on normality of model residuals will be satisfied. Nevertheless, it is possible that after careful evaluation of those assumptions, they are not adequately satisfied. In that case, we will use bootstrapping to obtain valid confidence intervals for intervention effect estimates. Alternative strategies will be either log-transforming the outcome variable or, for the Timed Up and Go test, adopting a time-to-event analysis using, for example, Cox proportional hazards models which would also enable us to better accommodate the censored times for individuals who could not complete the test.

### 7.3.2 Secondary effectiveness analyses of binary outcomes

Medication use, adherence, and physical activity levels are pre-specified binary outcomes. We will analyze these variables using the generalized estimating equations (GEE) approach to obtain population-averaged intervention effects. As with the primary analysis approach, strata will be included as fixed effects in the model. In contrast to using random intercepts in the linear mixed models used for the primary and continuous secondary outcomes, a working correlation matrix will be used to account for clustering.^61^ More specifically, the modified Poisson GEE approach (with log-link) will be used to obtain probability ratios (often referred to as risk ratios).^62^ Although we expect to see correlation of outcomes within clusters, an independent working correlation matrix will be used rather than exchangeable as it may provide greater stability with the anticipated variability in cluster size. Robust standard errors will be used to account for potential model misspecification. Importantly, because adherence can only be measured in those who are taking medications, it will be important to determine the validity of a between-arm comparison. To this end, we will compare the predictors of medication use in each arm, for each of the three medications, thereby evaluating whether there is good internal validity of the comparison. In the case that we identify differences between arms in characteristics of those taking medications, we will adjust for such characteristics in the regression model.

### 7.3.2 Exploratory effectiveness analyses

The exploratory outcomes of mortality, disability, stroke-related hospitalization, and stroke recurrence are binary variables. The modified Poisson GEE approach (with log-link) will be used to obtain risk ratios for binary outcomes, as described above.

## 7.4 Subgroup analyses

To explore the factors related to the effectiveness of the intervention, we will perform subgroup analyses for the following baseline variables: gender (male/female), age group (<65 vs. ≥65), education level (schooling vs. no schooling), years since last stroke events (less than 3 years vs. more than 3 years). These subgroup analyses will be performed by adding to the model both the variable and its interaction term with treatment arm. We caution that these analyses may not be powered.

## 7.5 Strategies to handle missing data in effectiveness analyses

The primary analysis will not use imputation for missing data because a priori, there is no reason to assume that missing data are not missing at random. However, we will identify if there are any baseline covariates related to loss to follow-up by summarizing baseline characteristics and treatment arm by loss to follow-up status.^63,64^ If we identify baseline covariates that are predictive of loss to follow-up, we will adjust for these covariates in the regression models. If loss to follow-up is not negligible, we will also consider implementing the pattern mixture approach to test for sensitivity to missing data assumptions.^65^

## 7.6 Reporting

We will report the study results in a manner consistent with the adaptation of the CONSORT extension reporting guidelines^66^ for cluster-randomized controlled trials and web-based and mobile health interventions.

#

# **8. QUALITY CONTROL AND PROJECT MANAGEMENT**

This section will introduce the quality control plan and project management plan.

## 8.1 Project management

The purpose of project management is to conduct interventions and evaluations strictly according to the protocol and the plan. Different levels of managers will be included in the project to take charge of project management at their levels.

One **Duke Kunshan Project Manager**

- monitor the work of county project officer
- coordinate baseline and evaluation surveys
- communicate with PI, County Project Officer and township managers, and village doctors and village health promoters if necessary
- communicate with county hospital and township hospitals regularly
- deal with emergencies when necessary

One **County Project Officer**

- monitor the recruitment of village doctors, village health promoters and patients in each village
- coordinate baseline and evaluation surveys
- with the support of APP, monitor the work of village doctors and village health promoters in intervention villages from five towns
- communicate with villages, township hospitals, county hospital and Duke Kunshan managers
- deal with emergencies when necessary

Five **township managers**

- support the recruitment of village doctors, village health promoters and patients
- coordinate baseline and evaluation surveys
- with the support of APP, monitor the work of five village doctors and five village health promoters in intervention villages of his/her town
- communicate with villages and higher-level managers

In addition, we have International Steering Committee, Policy Advisory Group and Technology Advisory Group which are composed of researchers from different universities and institutes, specialists from hospitals and government officers. Besides, a Community Advisory Group composed of local stakeholders will be established. They will support and give feedbacks on the project management.

##

## 8.2 Quality control for intervention

The purpose of quality control is to ensure the quality of project implementation throughout the trial; to objectively collect relevant data which will help with data analysis and interpretation; and to provide in-time feedbacks for the optimization of the research design. The quality control will mainly focus on validity, integrity and protocol compliance. Several key measures will be taken:

**Weekly monitoring via the SINEMA APP**

The SINEMA APP is not only a tool of intervention, but also a tool of quality control. Township managers will be required to monitor the work of village doctors and village health promoters in the intervention group via a build-in module in the APP and submit weekly reports to the county project officer.

**Phone-based audit**

Every month, the county project officer will call five randomly selected patients in the intervention group. They will be asked about performances of their village doctors and village health promoters, their medication and rehabilitation. The county project officer is required to send monthly reports to the Duke Kunshan project manager, summarizing the results of the audit.

**On-site supervision and audit**

Township managers and county project officer will periodically visit intervention villages during the intervention. The Duke Kunshan project manager or other members of the research will visit the study site every two months to inspect the quality of each steps. For important time points such as recruitment, baseline survey, training, intervention, follow-up surveys, we will visit the site more frequently and stay there for longer time. Records will be made after each site visiting.

## 8.3 Quality control for assessment

Quality control for outcome assessment will be conducted by following strategies. 1) All data collectors will be recruited from the center of disease prevention and control from a nearby county. Data collectors will be binded with the intervention allocation and will receive training for data collections. 2) All data collection will be conducted following same strategy in all villages and to all participants. 3) The research team will perform the quality control with onsite monitoring for all the data collection procedure.

## 8.4 Quality control for the entire study

The study will be supervised and audited by the international steering committee with at least one meeting per year to ensure the quality of the study.

# **9. APPENDIX**

## 9.1 Appendix A-1. Informed consent forms

### SINEMA project consent form for stroke patients

We sincerely invite you to take part in the SINEMA study (Nanhe Stroke Project). This project aims to help rural stroke patients to reduce risk of recurrence and promote health. It is conducted by Duke Kunshan University in collaborations with partners from Nanhe County.

**Research overview and procedure**

Your villages and other 49 villages has been selected to participate in this project. We plan to recruit about 25 people with stroke history in each village to participate in the project. The project will be last for 12 months.

During this project, your village have half chance to be recruited in the intervention group. In the intervention group, we will train village doctors to improve their capacity in managing chronic conditions among stroke patients that is within their normal scope of medical practices. We will give them an Android-based phone with an App designed for this project, as a tool to assist in patient information collection. We will also send text and voice messages to you and your family members about medication adherence and healthy lifestyle modification.

If you agree to participate, no matter which group your village will be assigned, you will need to do the following:

1. To complete a baseline survey (30-40 mins) conducted by trained surveyors before the pilot intervention starts. The baseline survey consists of a series of structured questionnaires to collect information including your demographic information, body measurements, blood pressure, stroke (and other diseases) history, disability and dependence, medication adherence, health status, activities of daily living, and mobility, etc.;
2. To complete an evaluation survey (30-40mins) conducted by trained surveyors at the 3-month and 12-month after the project initiation. The survey is same with the baseline survey.
3. After getting your approval, we will collect data from the local authorities about your medical expenditure through the medical insurance system, which will help us to evaluate your medical cost during the project period.

If your village is assigned as control group, you will get usual care as what you have received now. If your village is assigned as intervention group, you will need to do the following:

1. To receive service provided by our project, including regular follow-up by trained village doctors (10-15 mins each time, once per three weeks);
2. To receive daily text or voice messages of stroke-related information during one-year;
3. To receive short phone call survey (about 3 mins) if you are selected. We will randomly select one out of five patients every three weeks. If you are selected, we will ask you questions about your medical seeking behaviors in the past three weeks.
4. To receive interview (20-30mins) and provide comments and feedbacks on the project during or after the project if you are selected. We will randomly select 5 patients per village during the one-year period. The interview will be audio-recorded.

**Risk and Benefits**

Your participation in the research will lead to minimum risks, known and anticipated, regardless whether your village were assigned in intervention group or control group. In addition, the existing medical services you are receiving will not be affected by your participation in this research. You will receive voice and text messages regarding managing your chronic conditions, you should consider whether it is suitable for younger children in the family to read them and any potential influences the messages may have on them.

You will not get any direct benefit by participating this study. However, you will be aware of your blood pressure level and other health related measurements (weight, height, etc.) through the survey process.

**Payment**

You will not be paid for your participation in this research. Rather, we will give a gift equivalent to 10-20 RMB at the time of your enrollment.

**Confidentiality**

Your responses to the baseline survey, as well as the information collected during each visit to/by the village doctor, will be treated in a confidential manner. No one outside our research team including your family members, neighbors and the local health workers will have access to your personal information. All the data will be stored securely on password protected computers and servers. Your name and other identifying information will not be linked with the responses. These data records will be completely deleted three years after the end of the project. Any reportage or presentation of research results will be in the aggregate, and your information will not be identifiable.

**Withdrawal**

Participation in this pilot study is voluntary. You have the right to withdraw from it at any time without any fear of penalty or consequences.

**Questions**

If you have any questions, please feel free to ask at any time. We will try our best to answer them.

**STATEMENT OF CONSENT**

"The purpose of this study, procedures to be followed, risks and benefits have been explained to me. I have been allowed to ask questions, and my questions have been answered to my satisfaction. I have been told whom to contact if I have questions, to discuss problems, concerns, or suggestions related to the research, or to obtain information or offer input about the research. I have read this consent form and agree to be in this study, with the understanding that I may withdraw at any time. I have been told that I will be given a signed and dated copy of this consent form."

Please check the box if you agree to:

□accept text and voice message on my phone

□accept text and voice message on my family member’s phone

□agree to get your ID number and collect your medical expenditure data from insurance system by authorized staff working in the insurance related department.

________________________________________ ___________ ___________

Signature of Subject Date Time

_________________________________________ ___________ ___________

Signature of Person Obtaining Consent Date Time

***(Optional):***

**___________________________________ ____________ ___________**

Signature of Principal Investigator Date Time

**If applicable, add the following:**

_________________________________________ ___________ ___________

Signature of Legal Representative Date Time

__________________________________________

Relationship to Subject

Study PI：Lijing Yan

[lijing.yan@duke.edu](mailto:lijing.yan@duke.edu)

(+86) 0512 36657057

昆山杜克大学

Local Contractor: Mobai Hou

Nanhe County Department of health and family planning

Tele: 17732968998

Beijing Taintan Hospital Medical Ethics Committee:

Tele: (+86)010-67098555

### SINEMA project consent form for village doctors in 50 villages for survey

We sincerely invite you to take part in the SINEMA project (Nanhe Stroke Project). This project aims to help rural stroke patients to reduce risk of recurrence and promote health. It is conducted by Duke Kunshan University in collaborations with partners from Nanhe County.

**Research overview and procedure**

Your villages and other 49 villages has been selected to participate in this project. We plan to recruit 1 village doctors per village to participate in the project. We would like to invite you to take part in 2 surveys. The survey contains some of your personal information regarding your medical education and practice background and your understanding on the secondary prevention of stroke. The survey will be administered by yourself. Your response will not be linked with you at any time and none of your personal information will be used in publications or other dissemination of the results of this study

If you agree to take part in this project, you need to do the following:

1. Complete village doctor survey in recent days. The survey contains some of your personal information regarding your medical education and practice background and your understanding on the secondary prevention of stroke.
2. Complete a survey in 12-month from now. The survey is similar to the previous one without your personal information.

Each survey lasting about 30-45 minutes long.

**Risk and Benefits**

Risk: We expected no to minimum risk for you to participate in this study. Your response will not be shared to any authorities and will not affect your career development in anyway.

Benefits: Taking part in this project will not bring you any direct benefits. But the result of this study may help us to understand the capacity of village doctors in providing the secondary prevention of stroke in resource-limited settings.

**Payment**

We will not pay anything to you. But you will be provided with a gift about 10 yuan as a compensation for your time and efforts.

**Personal information security**

No data regarding your personal information (Name, phone number, ID number, etc.) will be collected during the survey. No one else except from the key research person will access to these data.

**Withdrawal**

Participation in this study is voluntary. You have the right to withdraw from it at any time without any fear of penalty or consequences.

**Questions**

If you have any questions, please feel free to ask at any time. We will try our best to answer them.

**STATEMENT OF CONSENT**

"The purpose of this study, procedures to be followed, risks and benefits have been explained to me. I have been allowed to ask questions, and my questions have been answered to my satisfaction. I have been told whom to contact if I have questions, to discuss problems, concerns, or suggestions related to the research, or to obtain information or offer input about the research. I have read this consent form and agree to be in this study, with the understanding that I may withdraw at any time. I have been told that I will be given a signed and dated copy of this consent form."

_________________________________________ ___________ ___________

Signature of Subject Date Time

_________________________________________ ___________ ___________

Signature of Person Obtaining Consent Date Time

***(Optional):***

**___________________________________ ____________ ___________**

Signature of Principal Investigator Date Time

**If applicable, add the following:**

_________________________________________ ___________ ___________

Signature of Legal Representative Date Time

__________________________________________

Relationship to Subject

Study PI：Lijing Yan

[lijing.yan@duke.edu](mailto:lijing.yan@duke.edu)

(+86) 0512 36657057

昆山杜克大学

Local Contractor: Mobai Hou

Nanhe County Department of health and family planning

Tele: 17732968998

Beijing Taintan Hospital Medical Ethics Committee:

Tele: (+86)010-67098555

### SINEMA pilot research consent form for stakeholder interview

Thank you for participating the SINEMA project. We want to have an interview with you to seek feedbacks from you about your experience in taking part in the SINEMA project.

You are invited to participate in the interview. If you agree to provide us with your feedbacks, you will be interviewed for about 30-40 minutes. We will ask you questions about the following four aspects, and the interview will be audio-recorded:

1. Your experience in taking part in the SINMEA project;
2. Challenges and difficulties you faced during the project;
3. Whether and how this project made some changes to your life;
4. Other feedbacks in implementing this project.

**Risk and Benefits**

Risk: Your participation in the research will lead to none to minimum risks. We will not share your response to any authorities and your response will not influence your personal right (such as career path for healthcare providers/ health seeking behaviors for patients).

Benefit: You will not get a direct benefit by participating in the interview. But your response will help us to better evaluate the SINMEA project. If the project is effective, you and other stroke patients could be benefit from it by implementing SINEMA model in a larger scale.

**Payment**

You will not be paid for your participation in this interview. Rather, we will give a gift equivalent to 10-20 RMB at the time of your enrollment.

**Confidentiality**

We will not collect any personal identifiable information from you during the interview. Your responses will be treated in a confidential manner. No one outside our research team will have access to your personal information. All the information will be stored securely on password protected computers and servers. Your name and other identifying information will not be linked with the data. Any reportage or presentation of research results will be in the aggregate, and your information will not be identifiable.

**Withdrawal**

Participation in this pilot study is voluntary. You have the right to withdraw from it at any time without any fear of penalty or consequences.

**Questions**

If you have any questions, please feel free to ask at any time. We will try our best to answer them.

**Authorization**

I have read (or someone explained) the information above and have had the opportunity to ask questions and receive answers.

**STATEMENT OF CONSENT**

"The purpose of this study, procedures to be followed, risks and benefits have been explained to me. I have been allowed to ask questions, and my questions have been answered to my satisfaction. I have been told whom to contact if I have questions, to discuss problems, concerns, or suggestions related to the research, or to obtain information or offer input about the research. I have read this consent form and agree to be in this study, with the understanding that I may withdraw at any time. I have been told that I will be given a signed and dated copy of this consent form."

_________________________________________ ___________ ___________

Signature of Subject Date Time

_________________________________________ ___________ ___________

Signature of Person Obtaining Consent Date Time

***(Optional):***

**___________________________________ ____________ ___________**

Signature of Principal Investigator Date Time

**If applicable, add the following:**

__________________________________________ ___________ __________

Signature of Legal Representative Date Time

__________________________________________

Relationship to Subject

Study PI：Lijing Yan

[lijing.yan@duke.edu](mailto:lijing.yan@duke.edu)

(+86) 0512 36657057

昆山杜克大学

Local Contractor: Mobai Hou

Nanhe County Department of health and family planning

Tele: 17732968998

Beijing Taintan Hospital Medical Ethics Committee:

Tele: (+86)010-67098555

### SINEMA project consent form for verbal autopsy

Thank you and your family member for participating the SINEMA project. We want to have an interview with you to collect information on cause of death of your family member.

I am very sorry to hear that a member of your household has passed away. Please accept my sympathies. For the purpose of assessing impacts of stroke on health outcome, we would like to collect further information on all deaths of participants of this project. I would like to invite you to participate in an interview as a main caretaker of [the deceased’s name].

If you agree to participate, we will interview you about the past health of the person in your family who recently died. The interview will last no more than 30 minutes and may be much shorter. We will be using a tablet to record all information.

**Risks and Benefits**

Risk: Your participation in the research will lead to none to minimum risks. We will not share your response to any authorities.

Benefit: You will not get a direct benefit by participating in the interview.

**Payment**

You will not be paid for your participation in this interview.

**Confidentiality**

We will not link your name to your answers, so the information we learn will not be connected to you. Your responses will be treated in a confidential manner. No one outside our research team will have access to your personal information. All the information will be stored securely on password protected computers and servers. Any reportage or presentation of research results will be in the aggregate, and your information will not be identifiable.

**Withdrawal**

Participation in this interview is voluntary. You have the right to withdraw from it at any time without any fear of penalty or consequences. You are free to decide not to participate in the interview. We understand that you might be sad about your family member’s death and you may not want to talk about the person. Even if you agree at first to take part, you are free to change your mind at any time and to quit the interview. If you want to stop, you can tell the person asking you questions that you want to stop the interview. You will not suffer any penalty.

**Questions**

If you have any questions, please feel free to ask at any time. We will try our best to answer them.

**Authorization**

I have read (or someone explained) the information above and have had the opportunity to ask questions and receive answers.

**STATEMENT OF CONSENT**

"The purpose of this study, procedures to be followed, risks and benefits have been explained to me. I have been allowed to ask questions, and my questions have been answered to my satisfaction. I have been told whom to contact if I have questions, to discuss problems, concerns, or suggestions related to the research, or to obtain information or offer input about the research. I have read this consent form and agree to be in this study, with the understanding that I may withdraw at any time. I have been told that I will be given a signed and dated copy of this consent form."

______________________________ ___________ ___________

Signature of Subject Date Time

______________________________ ___________ ___________

Signature of Person Obtaining Consent Date Time

## 9.2 Appendix A-2: Introduction about the development of SINEMA App

We collaborated with an IT team from China Mobile Research Institute on developing an application (the SINEMA APP) for this research. Duke Kunshan OIT and Duke University OIT were involved in the designing process, especially on the data security plan.

**Functions of SINEMA APP**

The SINEMA APP is a portable and efficient record keeping system used by village doctors (VDs) during patients’ visits. The primary function of the APP is to digitally record patients’ health-related status (such as blood pressure and medication adherence) and their current prescriptions. Other functions include: to review previous status and prescriptions of a patient; to remind VDs of patients who are about to miss a routine follow-up; to provide training materials and test on safe practice; to link with the gateway of message sending system. To clarify, no medical decision or medical advice will be provided through the SINEMA APP to VDs. **Figure 1** shows the structure of the SINEMA APP.


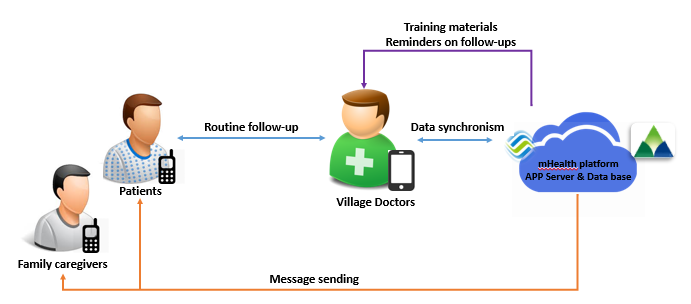


**Figure 1. The SINEMA APP and text-message system**

**Structure of SINEMA App System and server system**

The SINEMA App is composed of the front-end application and the back-end service. The APP is developed in the *Java* programming language on a local PC where an APK file is generated. The APK will be used to install the APP on an Android phone. The back-end service is also developed in the *Java* programming language with the development framework of SpringMVC and iBatis. The service will be installed in the APP server under the environment of Windows Server and Tomcat 7.0+. A MySQL database will be installed under Duke Kunshan Protected Network to host the encrypted data. Between the APP and APP server, data will be transmitted under the Hyper Text Transfer Protocol over Secure Socket Layer (HTTPS) to ensure data security. **Figure 2** shows the system structure of the SINEMA APP.


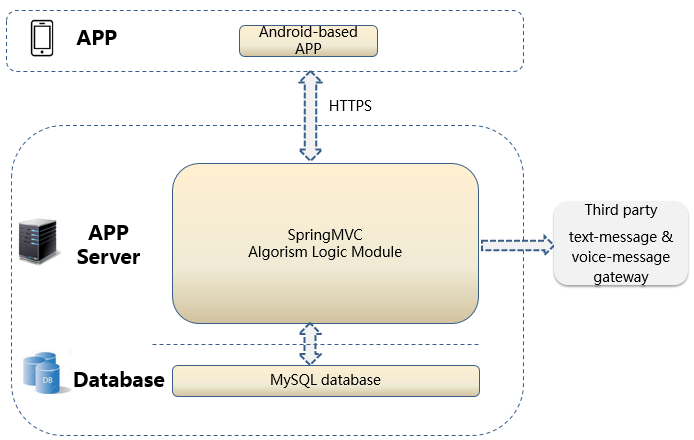


**Figure 2. The system structure of the SINEMA APP**

The server environment and system were established by IT team at Duke Kunshan University supervised by the IT team at Duke University. The SINEMA server and database server are all physically located in the data center at Duke Kunshan University.

The data security plan has been extensively reviewed by the IT team from China Mobile Research Institute and both Duke University and Duke Kunshan University OIT. A series of approaches were taken when designing the encryption process to minimize the potential risks and protect the data security at the highest level. **Figure 3** shows the data encryption process.


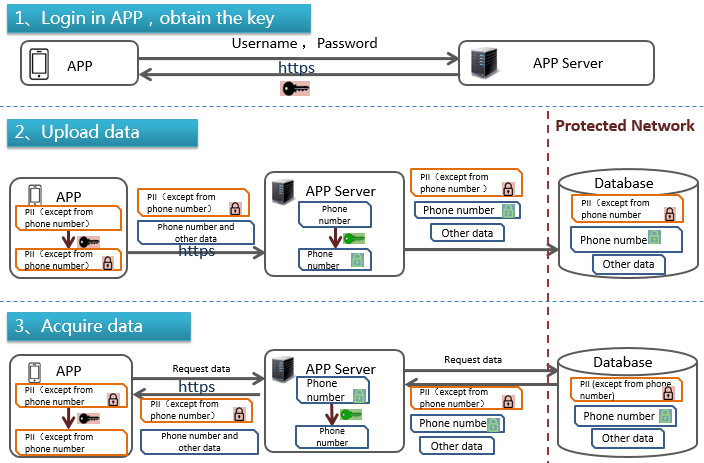


**Figure 3. The procedure for data encryption and protection**

- On the APP side, to prevent information leak because of phone lost, the APP users need to log in with their unique usernames and passwords each time they launch the APP. In addition, none of the information will be stored in the mobile devices.
- After the data entry, all data will be transmitted through Hyper Text Transfer Protocol over Secure Socket Layer (HTTPS), and stored in the database server to minimize the possibility of data breaches. To further protect the personal identifiable information (i.e. name), the data will be encrypted in the APP and then transferred through HTTPS and encrypted stored in the database. To be noticed, because cellphone numbers will be used to send patents text/voice messages, they will not be encrypted the same way as other personal identifiable information. Rather, the cellphone numbers will be transmitted under HTTPS and stored in the database.

**Access of SINEMA App**

The SINEMA App will be pre-installed on the smart-phone provided by the SINEMA project. Only research related staffs (village doctors in the intervention group, project officers at township and county level, research team in DKU) will have access to the SINEMA App, while other people outside of the study scope will not have access to the App in any way. The App will not be upload in any App store.

**Regulation and pilot study**

The SINEMA App is developed under the related regulation in China on data encryption and storage and will be implemented only in China under the related regulation. The App will only be used to support data collection during the VDs’ visit and guide VDs’ to practice based on the clinical guidelines. In line with the Chinese regulation on Mobile Health, the App cannot provide clinical decision in any way and there is no requirement from government or any kinds of authority to get the approval before using it.

A pilot study has been approved by Ethical Committee in Duke Kunshan University and the pilot study has shown that the SINEMA App is feasible and usable by VDs to fulfill the needs of this project.

**Follow-up Record for village Doctors**

**SINEMA Follow-up Record for village Doctors**

Date of follow-up visit 20YY/MM/DD

| **S1 Follow-up visit of the patients** |
| --- |
| S1.1 Whether the follow-up visit can be completed：☐ _1_ Yes (skip to 1) ☐ _2_ No (skip to S1.2)  S1.2 Reasons of this follow-up visit failing to complete:  ☐ 1 Patient is hospitalized due to illness ☐ 2 Patient are out of town ☐3 Patient has been deceased ☐4 Patient has quitted from the study |
| 1. **Referral** |
| 1.1 Do patients present with any of the following symptoms or conditions that may need immediate referral:  ☐ _1_ Symptoms of stroke during acute period: limb (with or without facial) weakness or numbness on one side; face numbness or mouth askew on one side; unclear speech or aphasis; both eyes gazing at one side; loss of vision of one or both eyes or being blurred; vertigo with vomiting; severe headache, vomiting rarely seen previously; disturbance of consciousness or convulsions; comprehensive cognitive impairment, such as reduction or loss of memory;  ☐ _2_ Other acute symptoms: chest pain, palpitation, shortness of breath, failing to supine, decreased urine volume (less than 400 ml within 24 hours)  ☐ _3_ Others: Any other symptoms that the village doctors cannot handle, for example: pregnant women or any other acute or severe symptoms  1.2 Patient's blood pressure: systolic pressure____ mmHg /diastolic pressure ______mmHg  End the follow-up and refer the patients to upper level hospitals if needed. |
| 1.3 From the last follow-up till now, whether the patients visited to other healthcare facilities because of cardiovascular and cerebrovascular diseases and related diseases (including stroke, heart disease, hypertension, diabetes, etc.)?  ☐ _1_ Yes, _______times ☐ _2_ No (skip to 2.1)  1.3a. Is the patient referred as a result of village doctors’ follow-up?☐ _1_ Yes ☐ _2_ No  1.3b Date of the visit: 20YY/MM/DD  1.3c Place of the visit: ☐ _1_ Xingtai municipal hospital ☐ _2_ Nanhe County People Hospital  ☐ _3_ Township hospital ☐ _4_ Other village clinic ☐ _5_ Others, _________________  1.3d Whether the patient was newly diagnosed with following diseases?  ☐ 1 ischemic stroke ☐ 2 hemorrhagic ☐ 3 coronary heart disease ☐ 4 diabetes  ☐ 5 hypertension ☐ 6 dyslipidemia ☐ 7 acute pulmonary embolism, asthma, severe pulmonary impairment and other pulmonary disorders ☐ 8 tumor or cancer ☐ 9 infection with high fever, unexplained dizziness, diabetes mellitus with infection ☐ 10 any other diseases that are not suitable for strenuous exercises) _______ ☐ 11 any other illness (please specify)  1.3f Does the upper level hospital make the medicine adjustment for the patient?☐ _1_ Yes ☐ _2_ No 1.3g. Hospitalized or not ☐ _1_ Yes, please fill in the length of stay: _____day ☐ _2_ No |
| 1. **Medication adherence** |
| 2.1 Medication adherence:  2.1a Did the patient forget to take medicine? ☐ _1_ Yes ☐ _0_ No  2.1b Have the patient paid less attention to taking medicine? ☐ _1_ Yes ☐ _0_ No  2.1c When the subjective symptom improves, whether the patients stopped medication? ☐ _1_ Yes ☐ _0_ No  2.1d When the subjective symptom deteriorates, whether the patients stopped medication?☐ _1_ Yes ☐ _0_ No  2.1e Fill in the following according to the patient medication record: the total number of days patients adhere to take medicine from the last visit to this follow-up _______days  2.1f Please upload the medication record chart of the patient [picture]  2.1g Main reasons leading to not adhering to take medication:  ☐ _1_ Doctor's suggestions ☐ _2_ Too expensive ☐ _3_ Forget to take medication ☐ _4_ Not effective  ☐ _5_ side effect ☐_6_ Other |
| 2.2 Does the patient have the following symptoms and side effect due to medication taking?  2.2.1a Side effect related to anti-hypertensive drugs:  2.2.1a1 Calcium antagonist ☐ 1 headache ☐ 2 edema  2.2.1a2 Angiotensin converting enzyme inhibitor (ACEI) ☐ 1 Cough ☐ 2 Angioneurotic edema ☐ 3 Renal dysfunction  2.2.1a3 Angiotensin receptor antagonist (ARB) ☐ 1 angioneurotic edema ☐ 2 renal impairment  2.2.1a4 Diuretics ☐ 1 hypokalemia ☐ 2 electrolyte imbalance  2.2.1a5 β blockers ☐ 1 heart function inhibition ☐ 2 bronchospasm  2.2.1a6 Compound preparation ☐ 1 impair renal function  2.2.1b Adverse effects associated with antiplatelet drugs: ☐ 1 Gastrointestinal symptoms (eg, stomach pain) ☐ 2 Skin and mucous membrane purpura ☐ 3 Gout ☐ 4 Gum bleeding  2.2.1c Adverse reactions associated with statins: ☐ 1 Muscle pain, rhabdomyolysis ☐ 2 Liver dysfunction symptoms ☐ 3 Gastrointestinal reactions (constipation, bloating, dyspepsia, and abdominal pain) ☐ 4 Insomnia ☐ 5 Others, please specify: ________ |
| 2.3 Does the patient need to adjust medication due to other reasons?  ☐ _1_ Yes, blood pressure is unqualified, and lack of drug efficacy ☐ _2_ Yes, patient's condition change ☐ _3_ Yes, other need of medication adjustment________☐ _4_ No (skip to 3) |
| 2.4 Please carefully adjust the medication according to clinical guidelines and patients’ condition. If unable to handle, please refer the patient to upper-level healthcare facility.  ☐ _1_ Modify the medicine prescription ☐ _2_  Referral is suggested (Please refer patients to the upper level healthcare facilities; please pay attention to whether the patient received treatment and continue monthly follow-up after patient's returning to the village [End of follow-up]) ☐ 3 Referral is suggested  2.5 Please record your prescription:  Drug classification, drug name 1, dosage, times of daily administration |
| **3. Overall Recommendations** |
| 3.1 Medication adherence:  If compliant, please encourage patients to continue to adhere to medication  If non-compliant, please explain to the patient about the importance of medication again, and encourage the patients to adhere to medication  3.2 Patient blood pressure:  Meet standard: Systolic blood pressure less than 140 and diastolic blood pressure less than 90  Please encourage the patients to continue on medication and keep a good lifestyle  Not meet the standard: systolic blood pressure greater than or equal to 140 or diastolic blood pressure greater than or equal to 90  Please explain to the patient about the importance of blood pressure control again, encourage patients to adhere to medication, and adjust the way of life  3.3 If the answer of 2.4 is ☐ 1 Modify the treatment regimen, please specify to the patients about the new medicines  3.4 Please give lifestyle recommendations to the patients one by one, and check the box after completion:  **Please advise patients:**  ☐ To reduce salt intake, including pickles, soy sauce and other high-salt foods  ☐ To quit smoking or to maintain non-smoking  ☐ To drink less alcohol, with a daily intake of no more than one bottle to two bottles of beer or 50 g to 75 g liquor for male; no more than half a bottle to one bottle of beer, or 25 g to 50 g liquor for female.  ☐ To reduce high-oil, high-fat food intake  ☐ To do some simple rehabilitation or exercises  ☐ To maintain a positive and healthy attitude. |

**Screenshots of SINEMA APP**


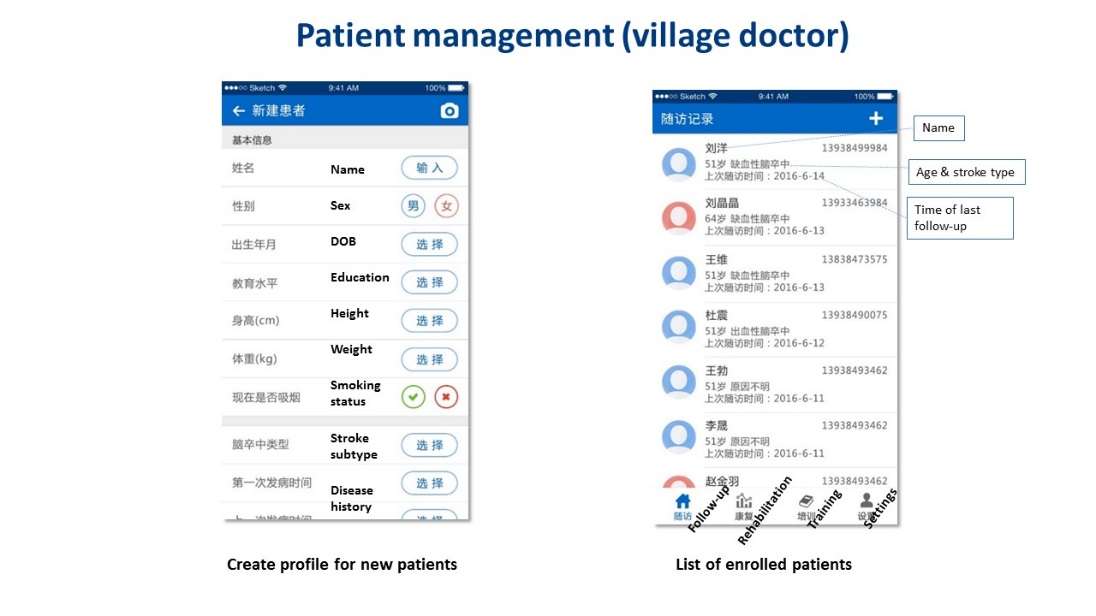

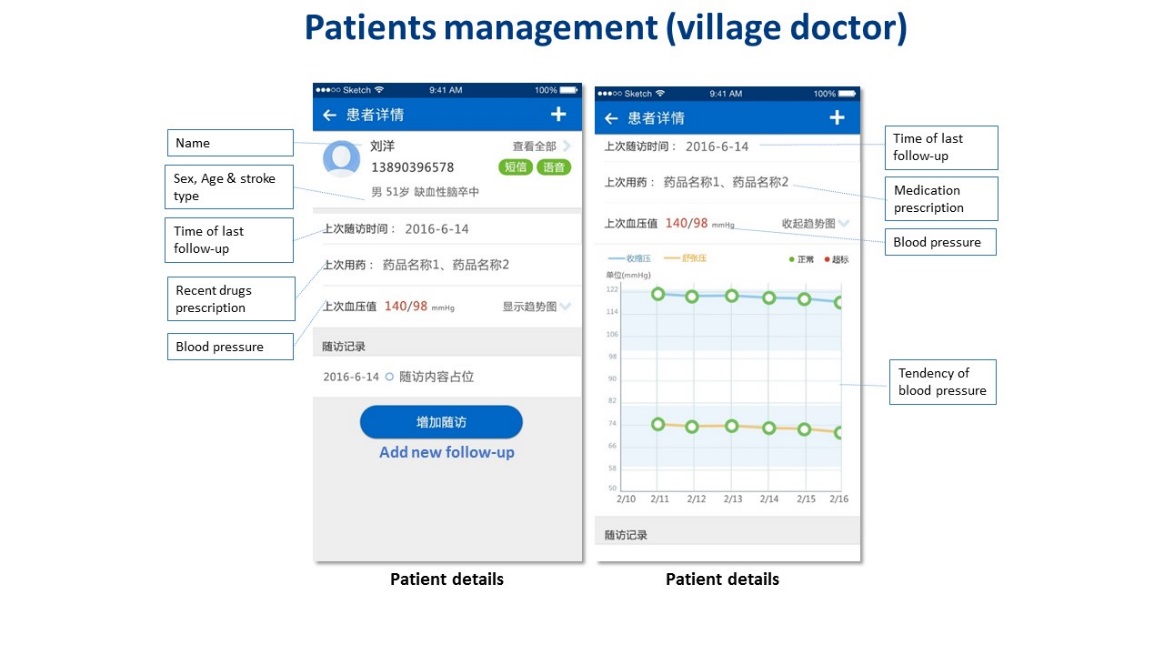

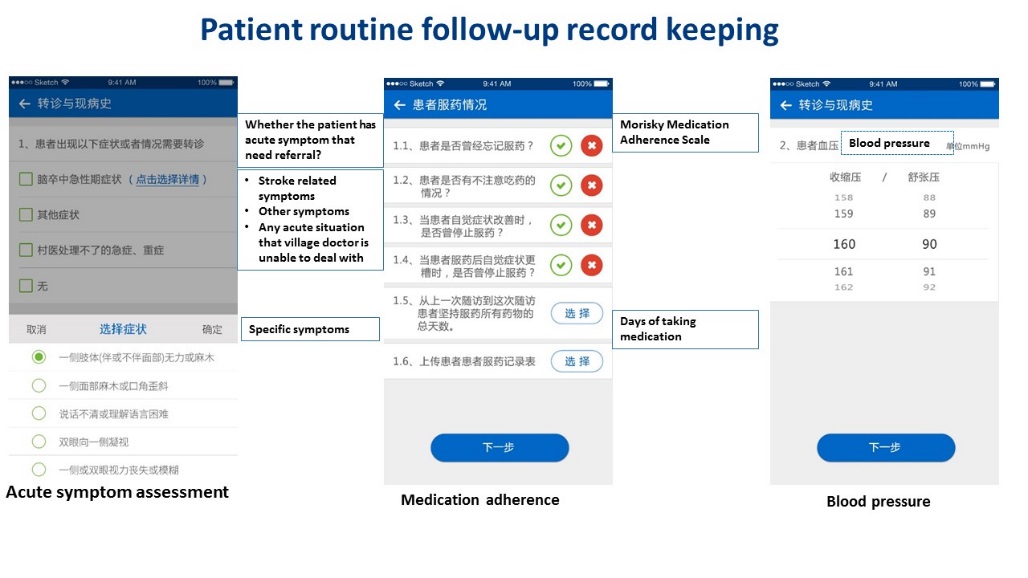

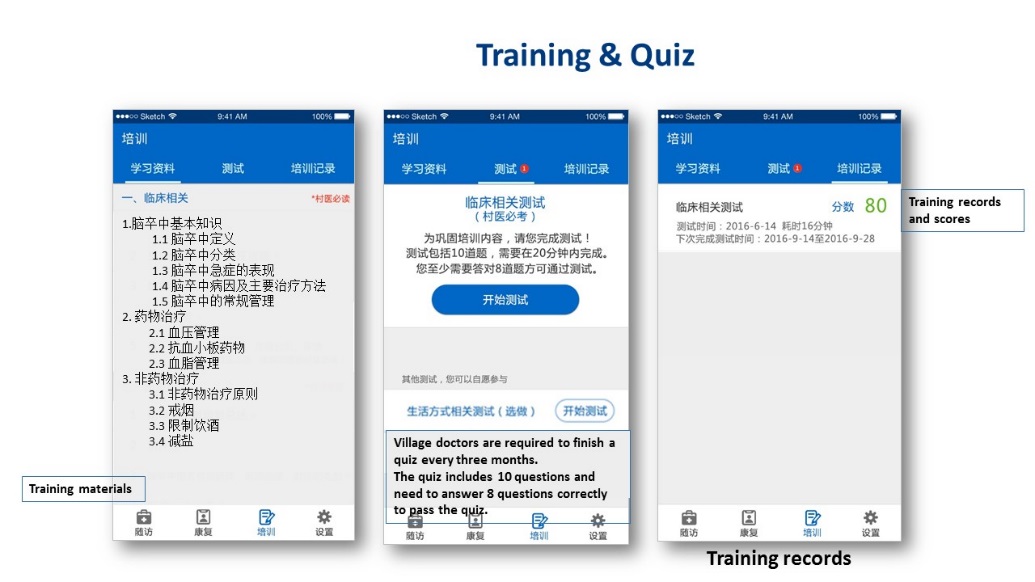


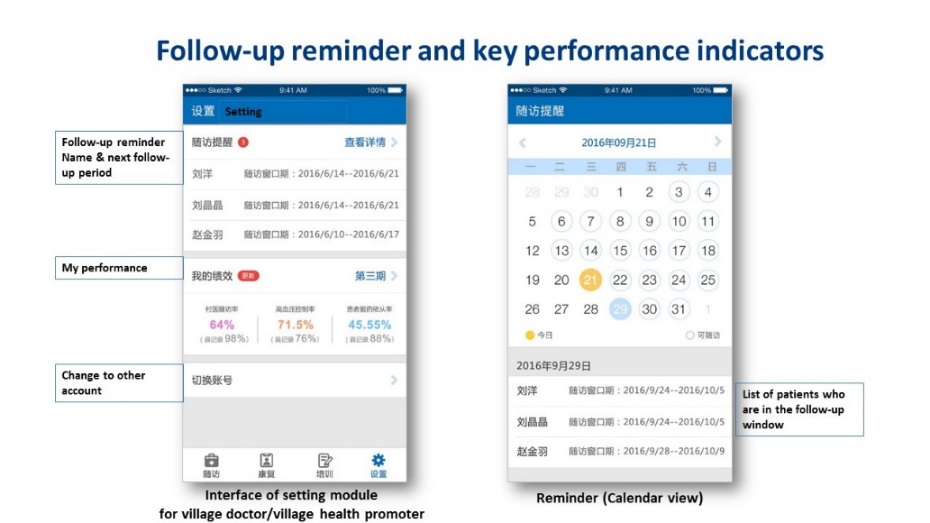


## 9.3 Appendix A-3. Questionnaire for participants’ survey at baseline and follow-up

***Note:***

Since we will use Qualtrics (electronic form) to collect data, the formatting of this paper version is not as consistent or as clear as it can be.

**BASIC INFORMATION**

Date of the interview: / /

Participant initial: ______ Participant Project ID: ______

Town: ______ Village: ______

Sex: ______ Year of birth: ______

Birth zodiacal animal: ______ Ethnicity: ______

*Marital status: ______ *Education: ______

*Occupation (before retirement): ______ *Occupation (after retirement): ______

Health insurance:

□Xingtai urban and rural resident basic health insurance

□Special non-communicable disease reimbursement

□other______

□Do not have any health insurance

**HOUSEHOLD/FAMILY INFORMATION**

Household size (how many people living in the household) and relationship: ______

Family income (yearly total):

<10K <20K <30K <40K 50K 60K 70K 80K 90K >100K >150K

Does your family own a TV? If so, when purchases? Price then in RMB? ______

Does your family own an automobile (not agricultural vehicle)? ______ If yes, make model? ______

Do you have a phone? □basic phone □smart phone □no phone

If you have a phone:

Do you know how to send a text message? □Yes □No

Do you know how to read a text message? □Yes □No

Do you know how to use any APP such as Wechat? □Yes □No

Do you share a phone with your family member? □Yes □No

**Main family health caregiver**

Who is your main family health caregiver? ______

Do you need his/her care? □Yes □No

How often does he/she remind you to take medicines? □Never □Seldom □Sometimes □Often □Always

Can his/her healthcare meet your needs?

Please enter the participant ID again: ______

**Stroke-related information**

When were you first diagnosed with stroke (only those diagnosed by county-level hospitals and or above)?

Year: ______ Month:_____ Type: ______ Diagnosed by/at: ______

Have you had recurrent stroke since then (only including episodes requiring a visit to or hospitalization at county-level hospitals or above)? How many times?

**Comorbidity**

Have you been diagnosed by county or higher level hospitals the following: Yes, No, don’t know

Heart disease ______

Hypertension ______

Diabetes ______

Cancer (any type) ______

Respiratory disease (any type) ______

Renal disease ______

Any other serious diseases? ______

**Hospitalization**

| During the past 12 months, have you been hospitalized? ________  If yes, how many times have you been discharged by the hospital? For each admission, please list the days spent in the hospital and for what conditions and total costs: | | | |
| --- | --- | --- | --- |
| ____________times | | | |
| Admission no. | Days spent in hospital | Diagnosis (conditions for which admitted) | Total costs (RMB) |
| 1 |  |  |  |
| 2 |  |  |  |
| 3 (add more if needed) |  |  |  |

**Village clinic**

How often did you visit the village clinic? □once a week or more □2~3 times per month □once per month □once per two months □less than once per two months

What are your main reasons of visiting the clinic? □measure blood pressure □but medicine □counseling

On average, how many minutes did the village doctor spend with you each time? _______

How many times did the village doctor measure your blood pressure in the past one month? _______

**Blood Pressure**

Please measure the participant’s blood pressure.

Reading #1: ______ / ______/Heart rate _______

Did you take any antihypertensive drugs today? □Yes □No

If yes:

When did you take the medicine?

Did you take the medicine for 5 or more days in the past one week? □Yes □No

Compare with the medicine you took in the past week:

The amount of the medicine you took today are □more □same □less

The type of the medicine took today are □same □different

**MEDICATION USE:**

Ask the following set of questions for aspirin, anti-hypertensive medicine, and lipid-lowering medicines for the past 12 months:

Have you been prescribed (been told by a doctor that you should take) aspirin during the past 12 months? ______ If yes, answer the following questions:

How many months have you taken aspirin tablets during the past 1 year?

Less than 2 months 2-8 months 9-11 months Every month

How many days usually in a month did you take it as prescribed?

≥25 days 20-24 days 15-19 days ≤14 days

Who prescribed the aspirin?

Self-prescribed Zang doctor Village doctor Specialist

Where did you buy the aspirin?

Village clinics Township center County hospital

If not taking it more than 25 days a month, also ask:

Reason you did not take it as prescribed?

Expensive Forget No effect Side effects Others, please specify____________

| *Instruct patients to bring ALL medicines they are currently taking in their original bottle/case/form.*    Have you taken any medication for your stroke or hypertension problems(s) during the past 1 month? If yes (or not sure what the med is for), fill in the form below: | | |
| --- | --- | --- |
| Medicine (brand name) | Use daily?  (>25days in the last 30 days) | Prescribed by village doctor? |
| 1. | □Yes □No | □Yes □No |
| 2. | □Yes □No | □Yes □No |
| 3. | □Yes □No | □Yes □No |
| 4. | □Yes □No | □Yes □No |
| 5. | □Yes □No | □Yes □No |

**Medication adherence**

**Morisky Medication Adherence Scale (MMAS-8)**

1. Do you sometimes forget to take your pills?
2. People sometimes miss taking their medications for reasons other than forgetting. Thinking over the past two weeks, were there any days when you did not take your medicine?
3. Have you ever cut back or stopped taking your medicine without telling your doctor because you felt worse when you took it?
4. When you travel or leave home, do you sometimes forget to bring along your medicine?
5. Did you take all your medicine yesterday?
6. When you feel like your symptoms are under control, do you sometimes stop taking your medicine?
7. Taking medicine every day is a real inconvenience for some people. Do you ever feel hassled about sticking to your treatment plan?
8. How often do you have difficulty remembering to take all your medicine?

**Disability and dependence**

**Modified Rankin Scale**

- No symptoms at all
- No significant disability despite symptoms; able to carry out all usual duties and activities
- Slight disability; unable to carry out all previous activities, but able to look after own affairs
- without assistance
- Moderate disability; requiring some help, but able to walk without assistance
- Moderately severe disability; unable to walk without assistance and unable to attend to own bodily
- needs without assistance
- Severe disability; bedridden, incontinent and requiring constant nursing care and attention

**HEALTH-RELATED QUALITY OF LIFE**

**EuroQOL Five Dimensions Questionnaire (EQ-5D)**

By placing a tick in one box in each group below, please indicate which statements best describe your own health state today.

MOBILITY

I have no problems in walking about  

I have slight problems in walking about  

I have moderate problems in walking about  

I have severe problems in walking about  

I am unable to walk about  

SELF-CARE

I have no problems washing or dressing myself  

I have slight problems washing or dressing myself  

I have moderate problems washing or dressing myself  

I have severe problems washing or dressing myself  

I am unable to wash or dress myself  

USUAL ACTIVITIES (e.g. work, study, housework, family or leisure activities)

I have no problems doing my usual activities  

I have slight problems doing my usual activities  

I have moderate problems doing my usual activities  

I have severe problems doing my usual activities  

I am unable to do my usual activities  

PAIN / DISCOMFORT

I have no pain or discomfort  

I have slight pain or discomfort  

I have moderate pain or discomfort  

I have severe pain or discomfort  

I have extreme pain or discomfort  

ANXIETY / DEPRESSION

I am not anxious or depressed  

I am slightly anxious or depressed  

I am moderately anxious or depressed  

I am severely anxious or depressed  

I am extremely anxious or depressed  

Visual Analogue Scale:

Rating health on a scale of 0-100 (best) for Today.

**Blood Pressure**

Please measure the participant’s blood pressure.

Reading #2: ______ / ______/Heart rate _______

**Smoking**

Do you smoke? Yes Smoked before but quit now Never

If yes:

How many cigarettes did you smoke every day?
 How many years have you been smoked?

Does your family members smoke? yes no

**Alcohol**

**Alcohol consumption, during the past 3 months.**

How often do you drink? ______

What kind of liquor do you often drink? ______

How much alcohol do you drink in one sitting? ______

**EXERCISE**

**1** During the last 7 days, on how many days did you perform **VIGOROUS** physical activities, e.g., carry water, jog, run, farm, climb or fast bicycling? Think about only those physical activities that you did for at least 10 minutes at a time.

________days a week □ (Go to 2) No vigorous physical activities □ (Go to 3)

**2** How much time did you usually spend doing **vigorous** physical activities on one of those days?

| ________hours per day  _______minutes per day Don’t know/Not sure□ |
| --- |

**3** During the last 7 days, on how many days did you do **moderate** physical activities like carrying light loads, bicycling at a regular pace, or doubles tennis?

________days a week □(Go to 4) No moderate phlysical activities □ (Go to 5)

**4** How much time did you usually spend doing **moderate** physical activities on one of those days? ________hours per day

_______minutes per day Don’t know/Not sure□

**5** During the last 7 days, on how many days did you walk at least 10 minutes at a time? This includes walking at work and at home, walking to travel from one place to another, doing housework and any other walking that you did solely for recreation, sports, exercise or leisure.

________days per week □ No walking □

**6** How much time did you spend **walking** on one of those days?

________hours per day

_______minutes per day Don’t know/Not sure□

**7** The last question is about the time you spent sitting on weekdays during the last 7 days. Include time spent at work, at home, while doing course work and during leisure time. This may include time spent sitting at a desk, visiting friends, reading, or sitting or lying down to watch television.

During the last 7 days, how much time in total did you usually spend sitting on one of those days?

________hours per day Don’t know □

_______minutes per day

**Diet**

1. During the last 12 months, have you attempted (paid attention) to reduce your intake in salt, sugar, oil, animal fat (ask 4 separately questions)?

□No □Yes □I do not eat it too much

1. How often do you eat fruits and vegetables (ask 2 separately questions)?

□every day □2~5 times per week □once a week □less than once a week □never

**knowledge**

Please judge the following statements. Whether it is right or wrong? □right □wrong □don’t know

1. Eating salty food will aggravate hypertension. □right □wrong □don’t know

2. Hypertension may lead to stroke. □right □wrong □don’t know

3. People get stroke because their blood vessel are blocked. □right □wrong □don’t know

4. Diabetes patients cannot eat any sweet food. □right □wrong □don’t know

5. Stroke patients cannot do any physical activities. □right □wrong □don’t know

6. The blood pressure of stroke patients should be controlled under 140/90mmHg. □right □wrong □don’t know

**Blood Pressure**

Please measure the participant’s blood pressure.

Reading #3: ______ / ______/Heart rate _______

**phq-9**

| **Over the past 2 weeks, how often have you been bothered by any of the following problems?** | **Not at all** | **Several days** | **More than half the days** | **Nearly every day** |
| --- | --- | --- | --- | --- |
| Little interest or pleasure in doing things |  |  |  |  |
| Feeling down, depressed or hopeless |  |  |  |  |
| Trouble falling asleep, staying sleep, or sleep too much |  |  |  |  |
| Feeling tired or have little energy |  |  |  |  |
| Poor appetite or overeating |  |  |  |  |
| Feeling bad about yourself-or that you are a failure or have let yourself or your family down |  |  |  |  |
| Trouble concentrating on things, such as reading the newspaper or watching TV |  |  |  |  |
| Moving or speaking so slowly that other people could have noticed. Or, the opposite-being so fidgety or restless that you have been moving around a lot more than usual |  |  |  |  |
| Thought that you would be better off dead or of hurting yourself in some way |  |  |  |  |

**Mobility-Timed Up and Go (TUG)**

On the word GO the patient will stand up, walk to the line on the floor (3 meters away from the chair) at his/her regular place, turn around and walk back to the chair and sit down.

Time (in seconds) needed to complete the task: ______

**body index measure**

Weight: _______kg Height: _______cm Waistline: _______cm

**BY INTERVIEWER**

| Interviewer comments/impressions:  □Interviewee understands questions well  □Interviewee understands questions normally  □Interviewee understands questions badly |
| --- |
| Interviewer double check and sign  Interviewer signature：_____________________________ |

## 9.4 Appendix A-4. Questionnaire for Village doctors’ survey

**Village doctor Survey**

Project Number:     Town: ______ Village: ______

1. **Basic Information**

A1. Gender: _1_ Male _2_ Female

A2. Birth date: / /

A3. Nationality: _1_ Han _2_ Other (Please specify) ________

A4. Educational Background: _1_Master _2_ Bachelor _3_ Junior college _4_ High school _5_ Vocational high school (Technical secondary school etc.) _6_ Junior high school or below

A5. Are you living in the village where you work? _1_ Yes _2_ No (Please specify)

A6. How many days can you work in the village clinic in the following year: __days per week

A7. Besides you, are there any of your other family members working in the village clinic? _1_ Yes _2_ No

If yes, please fill in the following information:

A7a. Relationship with you: _1_ Spouse _2_ Son _3_ Daughter _4_Father _5_ Mother _6_ Other（Please specify:_____）

A7b. His/Her position: _1_ Officially registered country doctors _2_ Officially registered physician's assistant_3_Pharmacist _4_ Unofficially registered physician’s assistant _5_  Other（Please specify:_____）

A8. Do you have a phone? _1_Smart phone _2_ Basic phone _3_ No phone

If yes, will you use it for the following things?

A8a.Sending and reading SMS? _1_Yes _2_ No

A8b.Reading and sending messages via Wechat? _1_Yes _2_ No

A8c. Surfing the internet? _1_Yes _2_ No

A8d. Have you ever used any medical related applications? _1_Yes (Please specify the name:____) _2_ No

A9. Do you have access to internet at your village clinic? _1_ Wired network _2_  Cable inner network (can connect to certain systems, but cannot connect to the outside world) _3_ Wi-Fi

1. **Medicine-Related Training**

B1. Total years of medicine-related training: Years

B2. Diploma of medicine-related major _1_Master _2_ Bachelor _3_ Junior college _4_ Vocational high school _5_ No/ Junior high school or below _6_ Other

If your answer is 1-4 or 6, please fill in the following information:

B2a. Graduation year:

B2b. Graduate school:______

B3. Have you attended any medical continuing education training after graduation? _1_Yes _2_ No

If yes, please fill in the following information:

B3a. Training institution:______

B3b. Training length month day

B3c. Finishing (Obtaining certificate) year:

**C. Medicine-related practical experience**

C1. Have you obtained any state-certified certificates of village doctors? _1_Yes _2_ No

If yes, please fill in the following information:

C1a. Which year did you obtain the certificate?

C2a. Which type of certificate did you obtain? _1_  Village doctor practicing certificate _2_ Physician’s assistant certificate

C2. In which year did you start to be a village doctor here?

C3. Before working here, have you ever worked in other places as a village doctor? _1_Yes _2_ No

If yes, please fill in the following information:

C3a. The name of the institution you have worked as a village doctor:________

C3b. Total years of working in other places as a village doctor:   Year

C4. During the past 12 months, on average, how many patients came to the village clinic per week? _____people/week

C5. During the past 12 months, on average, how many hypertension patients came to the village clinic per week? ______people/week

(Only if you have measured blood pressure and provided medication guidance; blood pressure measuring without any medical guidance does not count)

C6. For all the hypertension patients you have served in this village, your average follow-up visit frequency is: ____time(s)/month

C7. How do you think of your workload?

_1_ Very heavy _2_ heavy _3_ medium _4_ light _5_ Very light

C8. What is your salary range per month?

_1_ 6000 or above _2_ 5000-5999 _3_ 4000-4999_4_ 3000-3999 _5_ 2000-2999 _5_ 1000-1999 _6_ 999 or below _7_ not knowing

**D. Stroke secondary prevention knowledge**

D1. Please write down as many as risk factors that may predispose to stroke (open-ended question)

D2. Which conditions or life style habits may predispose to stroke? (multiple choice)

Hypertension Gastric ulcer Dyslipidemia Colitis Diabetes Constipation Obesity Prostatic hypertrophy SmokingAsthmaSedentary life-styleHeavy alcohol consumeBronchitis Previous strokeBiliary lithiasis Kidney lithiasis Familiar history of stroke Life pressure Heart disease

D3. Please write down as many as the signs of symptoms of acute stroke? (open-ended question)

D4. Within the following signs and symptoms, which are present in acute stroke? (multiple choice)

Speech impairmentCough Hemiparesis Dyspnea Facial palsy Abdominal ache Dizziness/Vertigo Chest pain Acute headacheFeverVisual alterationsDysuriaConfusion

D5. Control standards for the following groups:

D5a. For hypertensive patients, the goal is to lower BP to < ____/____ mmHg

D5b. The target level of glycosylated hemoglobin control for diabetes is: _1_ <5.5% _2_ <6% _3_ <6.5% _4_ <7%

D5c. What is the upper limit of liver enzyme for “statins” to be reduced or stopped?

D5d. What is the upper limit of creatase for “statins” to be reduced or stopped?

D5e. Which of the followings are the contraindications of aspirin? (multiple choice)

_1_ Cerebral hemorrhage _2_ Active bleeding _3_ Allergy to aspirin _4_ Asthma

**E.** **Attitude towards secondary prevention of stroke**

| Do you agree with the following statements? | Strongly  Disagree | Disagree | Neutral | Agree | Strongly  Agree |
| --- | --- | --- | --- | --- | --- |
| 1. I am very familiar with every stroke patient in my village. |  |  |  |  |  |
| 2. Primary care physicians are capable to provide stroke prevention-related services. |  |  |  |  |  |
| 3. Primary care physicians are capable to provide stroke treatment-related services. |  |  |  |  |  |
| 4. For people who have no stroke earlier, advise him to control risk factors as early as possible, rather than take precaution after stroke occurance. |  |  |  |  |  |
| 5. It is hard for people with stroke to return to manual work. |  |  |  |  |  |
| 6. The best option for stroke is treatment rather than prevention. |  |  |  |  |  |
| 7. Physical rehabilitation is as important as medicine treatment for stroke patients. |  |  |  |  |  |
| 8. Effective prevention can prevent the occurrence or reappearance of stroke. |  |  |  |  |  |

F. **Actions for secondary prevention of stroke**

| How often do you do the following actions for stroke patients in your village? | Rarely | Occasionally | Sometimes | Often | Always |
| --- | --- | --- | --- | --- | --- |
| Ask patients if they have adverse drug reaction before giving prescriptions. |  |  |  |  |  |
| Transfer patients to town hospitals or county hospitals if their systolic pressures exceed 180mmHg. |  |  |  |  |  |
| Encourage patients to change their unhealthy lifestyles. |  |  |  |  |  |
| Give your patients some physical rehabilitation treatments. |  |  |  |  |  |

## 9.5 Appendix A-5. Population health metrics research consortium shortened verbal autopsy questionnaire

**INTERVIEW BEGINS 开始访谈**

*Instructions to interviewer: Introduce yourself and explain the purpose of your visit. Ask to speak to the person who was the deceased’s main caretaker during the illness that led to death. If this is not possible, arrange a time to revisit the household when the caretaker will be home. (see example below).*

访谈员规范：请先介绍你自己并解释此次访谈的目的。请主动要求与在逝者病重期间承担照料工作的家属进行访谈。如果相关人员不在，可以另行预约时间。（见以下示例）

“My name is [your name]. I am an interviewer of the SINEMA study. I have been informed that a death has occurred in your household. I am very sorry to hear that a member of your household has passed away. Please accept my sympathies. For the purpose of assessing impacts of stroke on health outcome, we would like to collect further information on all deaths of participants of this project. I would like to talk to the main caretaker of [the deceased’s name] and ask some questions about the events and any symptoms that [the deceased’s name] had during her/his illness before death.”

“我的名字叫（自己的名字）。我是南和县脑卒中项目的一名访谈员。我知道您家中有人刚刚去世，我很理解您现在的感受，也深表同情。为了能进一步评估脑卒中带来的影响，我们正在收集这个项目中逝者的相关信息。正在本地收集近期逝者的相关信息。我想与逝者‘逝者姓名’病重期间承担照料工作的家属聊一聊‘逝者姓名’病重期间的一些情况。”

**SECTION 2 QUESTIONS ON THE DECEASED 第二部分 与逝者相关问题**

| gen_2_4 | Address of/directions to household  受访者家庭住址 |  |
| --- | --- | --- |

**SECTION 1 CONSENT** **第一部分 知情同意**

|  | ***INTERVIEWER: Read the consent form to the respondent. Ask the respondent if he or she has any questions. Once any questions are answered, ask the respondent if he or she is willing to take part in the study.***  访谈员：请为受访者读知情同意书。询问受访者是否有任何疑问。回答受访者疑问后询问其是否愿意参与调查 | | |
| --- | --- | --- | --- |
| gen_3_1 | Did respondent give consent?  受访者是否同意参与？ | Yes是  No 否 |    |
|  | ***If answer is “Yes” proceed to gen_5_0.***  “是”——跳至gen_5_0  ***If answer is “No” then thank respondent for their time and end the interview.***  “否”——对受访者表示感谢并结束访谈。 | | |

**SECTION 5 QUESTIONS ON THE DECEASED 第五部分 与逝者相关问题**

| gen_5_0 | What was the name of the deceased? 死者姓名？ | [_____________] |  |
| --- | --- | --- | --- |
| gen_5_1a | What year was the deceased born?  死者在哪一年出生？ | [__]__]__]__]  *Enter 9999 if unknown*  如果不知道录入“9999”  Year must be between 1880 and this year. 年份必须在1880和现在之间 |  |
| gen_5_1b | What month was the deceased born?  死者在哪个月出生？ | [__]__]  *Enter 99 if unknown*  如果不知道录入“99”  Month must be between 1 and 12.  录入的月份需要在1-12之间 |  |
| gen_5_1c | What day of the month was the deceased born?  死者在几号出生？ | [__]__]  *Enter 99 if unknown*  如果不知道录入“99”  *Day must be between 1 and 31.*  录入的日期需要在1-31之间 |  |
| gen_5_2 | What was the sex of the deceased?  死者的性别？ | 1. Male 男  2. Female 女 |  |
| gen_5_3a | What year did the deceased die?  死者在哪一年去世？ | [__]__]__]__]  *Enter 9999 if unknown*  如果不知道录入“9999”  Year must be between 1880 and this year. 录入的年份需要在1880至本年度之间 |  |
| gen_5_3b | What month did the deceased die?  死者在哪个月去世？ | [__]__]  Enter 99 if unknown  如果不知道录入“99”  Month must be between 1 and 12.  录入的月份需要在1-12之间 |  |
| gen_5_3c | What day of the month did the deceased die?  死者在几号去世？ | [__]__]  *Enter 99 if unknown*如果不知道录入“99”  *Day must be between 1 and 31.*  录入的日期需要在1-31之间 |  |
| gen_5_4 | What was the last known age of the deceased?  死者去世时的年龄多大？  (Less than 24 hours = 00 days. Enter age in days up to 27 days. Enter 28 days as 1 month. From 1-11 months enter age in months. Enter 12 months as 1 year. From 1 year enter age in years.)  （不足24小时=0天。27天及以下以天为单位填写年龄。28天以上填写为1个月。1-11个月以月为单位填写年龄。12个月填写成1年。1年及以上以年为单位填写年龄） | 1. __ __ days天  *Enter 99 if unknown*如果不知道录入“99”  2. __ __ months月  *Enter 99 if unknown*如果不知道录入“99”  3. __ __ years年  *Enter 99 if unknown*如果不知道录入“99”  8. Refused to answer 拒绝回答  9. Don’t know 不知道 |    |
|  | *If “Refused to answer” or “Don’t know”, go to gen_5_4d*  “拒绝回答”或“不知道”——跳至*gen_5_4d* | |  |
| gen_5_4d | What age group does the deceased's last known age fall into?  若死者年龄不详，死者属于以下哪个年龄段 | 1. Less than 28 days 不足28天  2. 28 days – 11 years 28天-11岁  3. Older than 12 years 大于等于12岁  8. Refused to answer拒绝回答  9. Don’t Know不知道 |          |
|  | *If age and age group are both “Don’t know” or “Refused to answer”, thank respondent for their time and end the interview.* 如果对逝者年龄及年龄段的回复均为“拒绝回答”或“不知道”，对受访者表示感谢并结束访谈。 | | |
| gen_5_5 | Where did the deceased die?  死亡地点？ | Hospital医院  Other health facility其他医疗机构  On route to hospital or other health facility  去医院或其他医疗机构途中  Home 家中  Other (specify _________)  其他（请详述_________）  8. Refused to answer 拒绝回答  9. Don’t know 不知道 |              |

**SECTION 6 QUESTIONS ON THE NOTIFICATION OF DEATH 第六部分 死亡登记相关问题**

| gen_6_1 | Has this death been registered?  死亡进行过登记么？  *(refer to local registration process, e.g. with the civil registry office)*参考当地登记流程 | 1. Yes 是  2. No 否  8. Refused to answer 拒绝回答  9. Don’t know 不知道 | ****  ****  ****  **** |
| --- | --- | --- | --- |
|  | *If “No”, “Refused to answer” or “Don’t know”, go to gen_6_4*  “否”或“拒绝回答”或“不知道”——跳至*gen_6_4* | | |
| gen_6_2 | Record the date of registration  记录登记日期  *Enter 9999 if unknown*  如果不知道录入“9999” | _ _/_ _/_ _ _ _  dd mm yyyy  天 月 年 |  |
| gen_6_3 | Record the registration number:  记录登记号 |  |  |
| gen_6_4 | *Has this death been notified?*  开具死亡单了么？  *(question for the interviewer)*  访谈员回答 | 1. Yes 是  2. No否  9. Don’t know 不知道 | ****  ****  **** |
|  | *If “No”, “Refused to answer” or “Don’t know”, go to gen_6_6*  “否”或“拒绝回答”或“不知道”——跳至*gen_6_6* | | |
| gen_6_5 | Record notification number:  记录死亡单号 |  |  |
| gen_6_6 | Did the decedent have a National ID number?  逝者有身份证号码么 | 1. Yes 是  2. No否  3. Too young for a National ID number年龄小没有身份证号  4. Not relevant 不相关  8. Refused to answer拒绝回答  9. Don’t know 不知道 | ****  ****  ****  ****  ****  **** |
|  | *If “Yes”, go to gen_6_7*  *If “Too young for a National ID number”, go to gen_6_8*  *If “No”, “Not Relevant”, “Refused to answer” or “Don’t know”, go to specific age group VA module*  “是”——跳至gen_6_7  “年龄小没有身份证号”——跳至gen_6_8  “否”或“不相关”或“拒绝回答”或“不知道”——跳至VA中的相应年龄组模块 | | |
| gen_6_7 | Record the National ID number:  记录身份证号码 |  |  |
|  | *Go to specific age group VA module*  跳至*VA*中的相应年龄组模块 | | |
| gen_6_8 | Does one of the parents have a National ID number?  父母中的一位是否有身份证号码？ | 1. Yes 是  2. No否  8. Refused to answer拒绝回答  9. Don’t know 不知道 | ****  **** |
|  | *If “No”, “Refused to answer” or “Don’t know”, go to specific age group VA module*  “否”或“拒绝回答”或“不知道”——跳至*VA*中的相应年龄组模块 | | |
| gen_6_9 | Which parent will we record?  记录父亲还是母亲？ | 1. Mother母亲  2. Father父亲 | ****  **** |
| gen_6_10 | Record the parent’s National ID number:  其身份证号码为： |  |  |

If deceased was 12 years or older, begin Adult VA module.

If deceased was 28 days or older and younger than 12 years, begin the Child VA module.

If deceased was less than 28 days old, go to the Neonate VA module.

如果逝者大于等于12岁，使用VA成人模块。如果逝者大于等于28天小于12岁，使用VA儿童模块。如果逝者小于28天，使用VA新生儿模块。

**All our participants are older than 18 years old, so we use the adult module.**

**POPULATION HEALTH METRICS RESEARCH CONSORTIUM 人群健康测量联合研究**

**SHORTENED VERBAL AUTOPSY INSTRUMENT 死因推断（短版）工具**

**SECTION 5 INJURIES AND ACCIDENT 第五部分 伤害和意外**

| adult_5_1  成人_5_1 | Did ________ suffer from an injury or accident that led to his/her death?  ______是否曾遭受过致其死亡的伤害或意外事故？ | 1. Yes 是  2. No否  8. Refused to answer拒绝回答  9. Don’t know 不知道 | **** |
| --- | --- | --- | --- |
|  | *If “No”, refused to answer or don’t know is checked, go to Section 1.*  *如果回答“否”、“拒绝回答”或“不知道，转至第一部分”* | |  |
| adult_5_2  成人_5_2 | What kind of injury or accident did ____________suffer from?  Ask respondent each in sequence and mark all to which the respondent indicated “Yes.”  ______曾遭受过以下哪种伤害或意外事故？（请按顺序询问受访者每个问题，并勾选受访者回答“是”的所有选项） | 1. Road traffic crash/injury 道路交通碰撞  2. Fall 坠落  3. Drowning 溺水  4. Poisoning 中毒  5. Bite or sting by venomous anima 被有毒动物咬伤或叮咬l  6. Burn/fire 烧伤、火灾  7. Violence (suicide, homicide, abuse) 暴力（自杀、杀人、滥用）  11. Other injury, specify________其他伤害，特指________  8. Refused to answer 拒绝回答  9. Don’t know 不知道 | ****  ****  ****  ****  ****  ****  ****  **** |
| adult_5_3  成人_5_3 | Was the injury or accident self-inflicted? 这起伤害或意外事故是否是其本人造成的？ | 1. Yes 是  2. No否  8. Refused to answer拒绝回答  9. Don’t know 不知道 |  |
| adult_5_4  成人_5_4 | Was the injury or accident intentionally inflicted by someone else? 这起伤害或意外事故是否是他人故意造成的？ | 1. Yes 是  2. No否  8. Refused to answer拒绝回答  9. Don’t know 不知道 | ****  **** |
|  | ***Go to Section 6: HEALTH RECORDS 进入第六部分：健康档案*** | |  |

**SECTION 1 HISTORY OF CHRONIC CONDITIONS OF THE DECEASED 第一部分 死者病史及慢性病情况**

| adult_1_1  成人_1_1 | Was ______ ever told by a health professional that he or she ever suffered from one of the following?  医疗工作者是否曾告知您________患有下列任何疾病？ | | |
| --- | --- | --- | --- |
| adult_1_1a  成人_1_1a | Asthma  哮喘 | 1. Yes 是  2. No否  8. Refused to answer拒绝回答  9. Don’t know 不知道 | ****  **** |
| adult_1_1c | Cancer  癌症 | 1. Yes 是  2. No否  8. Refused to answer拒绝回答  9. Don’t know 不知道 | ****  **** |
| adult_1_1m | COPD (Chronic Obstructive Pulmonary Disease)  慢性阻塞性肺病 | 1. Yes 是  2. No否  8. Refused to answer拒绝回答  9. Don’t know 不知道 | ****  **** |
| adult_1_1g | Diabetes  糖尿病 | 1. Yes 是  2. No否  8. Refused to answer拒绝回答  9. Don’t know 不知道 | ****  **** |
| adult_1_1h | Epilepsy  癫痫 | 1. Yes 是  2. No否  8. Refused to answer拒绝回答  9. Don’t know 不知道 | ****  **** |
| adult_1_1i | Heart Disease  心脏病 | 1. Yes 是  2. No否  8. Refused to answer拒绝回答  9. Don’t know 不知道 | ****  **** |
| adult_1_1d | Tuberculosis  结核病 | 1. Yes 是  2. No否  8. Refused to answer拒绝回答  9. Don’t know 不知道 | ****  **** |
| adult_1_1l | Stroke  中风 | 1. Yes 是  2. No否  8. Refused to answer拒绝回答  9. Don’t know 不知道 | ****  **** |
| adult_1_1n | AIDS  艾滋病 | 1. Yes 是  2. No否  8. Refused to answer拒绝回答  9. Don’t know 不知道 | ****  **** |

**SECTION 2 SYMPTOM CHECKLIST 第二部分 症状检查清单**

| adult_2_2  成人_2_2 | Did _____ have a fever?  ______是否有发烧症状？ | 1. Yes 是  2. No否  8. Refused to answer拒绝回答  9. Don’t know 不知道 | ****  **** |
| --- | --- | --- | --- |
|  | *If “No” or “Don’t know” or “Refused to answer” go to adult_2_7* 如果回答“否”、“不知道”、“拒绝回答”请转至“成人_2_7” | | |
| adult_2_4  成人_2_4 | How severe was the fever?发烧严重程度 | 1. Mild轻度  2. Moderate中等程度  3. Severe严重  8. Refused to answer拒绝回答  9. Don’t know 不知道 | ****  ****  **** |
| adult_2_5  成人_2_5 | What was the pattern of the fever?发烧类型是什么？ | 1. Continuous持续型  2. On and off间断型  3. Only at night 仅仅晚上  8. Refused to answer拒绝回答  9. Don’t know 不知道 | ****  ****  **** |
| adult_2_7  成人_2_7 | Did _____ have a rash?  ______是否有出疹症状？ | 1. Yes 是  2. No否  8. Refused to answer拒绝回答  9. Don’t know 不知道 | **** |
|  | *If “No” or “Don’t know” or “Refused to answer” go to adult_2_10* 如果回答“否”、“不知道”、“拒绝回答”请转至“成人_2_10” | | |
| adult_2_9  成人_2_9 | Where was the rash located?  哪个部位出疹？ | 1. Face 脸  2. Trunk躯干  3. Extremities 四肢  4. Everywhere到处  8. Refused to answer拒绝回答  9. Don’t know 不知道 | ****  ****  ****  **** |
| adult_2_10  成人_2_10 | Did ____ have sores?  ______是否有生疮症状？ | 1. Yes 是  2. No 否  8. Refused to answer拒绝回答  9. Don’t know 不知道 | ****  **** |
|  | *If “No” or “Don’t know” or “Refused to answer” go to adult_2_13*  如果回答“否”、“不知道”、“拒绝回答”请转至*“成人_2_13”* | |  |
| adult_2_11  成人_2_11 | Did the sores have clear fluid or pus?  生疮部位是否流透明液体或脓液？ | 1. Yes 是  2. No否  8. Refused to answer拒绝回答  9. Don’t know 不知道 | ****  ****  **** |
| adult_2_13  成人_2_13 | Did ______ have an ulcer (pit) on the foot?  ______是否有足部溃疡（点状）症状？ | 1. Yes 是  2. No否  8. Refused to answer拒绝回答  9. Don’t know 不知道 | ****  ****  **** |
|  | *If “No” or “Don’t know” or “Refused to answer” go to adult_2_21*  如果回答“否”、“不知道”、“拒绝回答”请转至“成人*_2_21”* | | |
| adult_2_14  成人_2_14 | Did the ulcer ooze pus?  溃疡部位是否有脓液渗出？ | 1. Yes 是  2. No否  8. Refused to answer拒绝回答  9. Don’t know 不知道 | ****  ****  **** |
|  | *If “No” or “Don’t know” or “Refused to answer” go to adult_2_21*  如果回答“否”、“不知道”、“拒绝回答”请转至“成人*_2_21”* | |  |
| adult_2_15  成人_2_15 | For how many days did the ulcer ooze pus?  溃疡部位有脓液渗出的情况持续了几天？ | 1. __ __ days 天 *Enter 99 if unknown如果不知道输入99*  8. Refused to answer 拒绝回答  9. Don’t know不知道 |  |
| adult_2_21  成人_2_21 | Did _____ have yellow discoloration of the eyes?  ______是否有双眼泛黄症状？ | 1. Yes 是  2. No否  8. Refused to answer拒绝回答  9. Don’t know 不知道 | ****  **** |
|  | *If “No” or “Don’t know” or “Refused to answer” go to adult_2_25*  *如果回答“否”、“不知道”、“拒绝回答”请转至“成人_2_25”* | | |
| adult_2_22  成人_2_22 | For how long did ______ have the yellow discoloration?  ______的双眼泛黄症状持续了多久？ | 1. __ __ days 天 *Enter 99 if unknown*如果不知道输入99  2. __ __ month 月 *Enter 99 if unknown*如果不知道输入99  8. Refused to answer拒绝回答  9. Don’t know 不知道 |  |
| adult_2_25  成人_2_25 | Did _____ have puffiness of the face?  _____是否有脸部浮肿症状？ | 1. Yes 是  2. No否  8. Refused to answer拒绝回答  9. Don’t know 不知道 | ****  ****  **** |
|  | *If “No” or “Don’t know” or “Refused to answer” go to adult_2_27*  如果回答“否”、“不知道”、“拒绝回答”请转至“成人*_2_27”* | |  |
| adult_2_26  成人_2_26 | For how long did ______ have puffiness of the face?  _____的脸部浮肿症状持续了多久？ | 1. __ __ days 天 *Enter 99 if unknown*如果不知道输入99  2. __ __ month 月 *Enter 99 if unknown*如果不知道输入99  8. Refused to answer拒绝回答  9. Don’t know 不知道 |  |
|  | | | |
| adult_2_27  成人_2_27 | Did _______ have general puffiness all over his/her body?  _____是否有全身浮肿症状？ | 1. Yes 是  2. No否  8. Refused to answer拒绝回答  9. Don’t know 不知道 | ****  **** |
| adult_2_29  成人_2_29 | Did _____ have a lump in the neck?  _____的颈部是否有肿块？ | 1. Yes 是  2. No否  8. Refused to answer拒绝回答  9. Don’t know 不知道 | ****  **** |
| adult_2_30  成人_2_30 | Did _____ have a lump in the armpit?  _____的腋下是否有肿块？ | 1. Yes 是  2. No否  8. Refused to answer拒绝回答  9. Don’t know 不知道 | ****  **** |
| adult_2_31  成人_2_31 | Did _____ have a lump in the groin?  _____的腹股沟处是否有肿块？ | 1. Yes 是  2. No否  8. Refused to answer拒绝回答  9. Don’t know 不知道 | ****  **** |
| adult_2_32  成人_2_32 | Did _____ have a cough?  _____是否有咳嗽症状？ | 1. Yes 是  2. No否  8. Refused to answer拒绝回答  9. Don’t know 不知道 | ****  **** |
|  | *If “No” or “Don’t know” or “Refused to answer” go to adult_2_36*  如果回答“否”、“不知道”、“拒绝回答”请转至“成人*_2_36”* | |  |
| adult_2_34  成人_2_34 | Did the cough produce sputum?  咳嗽时是否咳痰？ | 1. Yes 是  2. No否  8. Refused to answer拒绝回答  9. Don’t know 不知道 | ****  **** |
| adult_2_35  成人_2_35 | Did _____ cough blood?  _____咳嗽时是否咳血？ | 1. Yes 是  2. No否  8. Refused to answer拒绝回答  9. Don’t know 不知道 | ****  ****  **** |
| adult_2_36  成人_2_36 | Did _____ have difficulty breathing?  _____是否有呼吸困难症状？ | 1. Yes 是  2. No否  8. Refused to answer拒绝回答  9. Don’t know 不知道 | ****  ****  **** |
| adult_2_43  成人_2_43 | Did _____ experience pain in the chest in the month preceding death?  _____在去世之前的当月内，是否有胸痛症状？ | 1. Yes 是  2. No否  8. Refused to answer拒绝回答  9. Don’t know 不知道 | ****  ****  **** |
|  | *If “No” or “Don’t know” or “Refused to answer” go to adult_2_47*  *如果回答“否”、“不知道”、“拒绝回答”请转至“成人_2_47”* | |  |
| adult_2_44  成人_2_44 | How long did the pain last?  胸痛症状持续了多久？ | 1. Less than 30 minutes 少于30分钟  2. 30 minutes to 24 hours 30分钟至24 小时  3. More than 24 hours 多于24小时  8. Refused to answer拒绝回答  9. Don’t know 不知道 | ****  ****  ****  **** |
|  | | | |
| adult_2_47  成人_2_47 | Did _____ have more frequent loose or liquid stools than usual?  _____排粥样便的频率是否胜于以往？ | 1. Yes 是  2. No否  8. Refused to answer拒绝回答  9. Don’t know 不知道 | ****  ****  **** |
| adult_2_50  成人_2_50 | Was there blood in the stool?  便中是否有带血的情形？ | 1. Yes 是  2. No否  8. Refused to answer拒绝回答  9. Don’t know 不知道 | ****  ****  **** |
|  | *If “No” or “Don’t know” or “Refused to answer” go to adult_2_52*  如果回答“否”、“不知道”、“拒绝回答”请转至“成人*_2_52”* | | |
| adult_2_51  成人_2_51 | Was there blood in the stool up until death?  便中带血的情况是否持续直至去世？ | 1. Yes 是  2. No否  8. Refused to answer拒绝回答  9. Don’t know 不知道 | ****  ****  **** |
| adult_2_52  成人_2_52 | Did _________ stop urinating?  _____是否停止排尿？ | 1. Yes 是  2. No否  8. Refused to answer拒绝回答  9. Don’t know 不知道 | ****  ****  **** |
| adult_2_53  成人_2_53 | Did _____ vomit in the week preceding the death?  _____在去世之前的当周内，是否有呕吐症状？ | 1. Yes 是  2. No否  8. Refused to answer拒绝回答  9. Don’t know 不知道 | ****  ****  **** |
|  | *If “No” or “Don’t know” or “Refused to answer” go to adult_2_57*  如果回答“否”、“不知道”、“拒绝回答”请转至“成人*_2_57”* | |  |
| adult_2_55  成人_2_55 | Was there blood in the vomit?  _____在去世之前，呕吐物中是否带血？ | 1. Yes 是  2. No否  8. Refused to answer拒绝回答  9. Don’t know 不知道 | ****  ****  **** |
| adult_2_56  成人_2_56 | Was the vomit black?  呕吐物是否呈黑褐色？ | 1. Yes 是  2. No否  8. Refused to answer拒绝回答  9. Don’t know 不知道 | ****  ****  **** |
| adult_2_57  成人_2_57 | Did _____ have difficulty swallowing?  _____是否有吞咽困难的症状？ | 1. Yes 是  2. No否  8. Refused to answer拒绝回答  9. Don’t know 不知道 | ****  ****  **** |
|  | *If “No” or “Don’t know” or “Refused to answer” go to adult_2_60*  *如果回答“否”、“不知道”、“拒绝回答”请转至“成人_2_60”* | |  |
| adult_2_58  成人_2_58 | For how long before death did ______ have difficulty swallowing?  _____在去世之前，吞咽困难的症状持续了多久？ | 1. __ __ days 天 *Enter 99 if unknown*如果不知道输入99  2. __ __ month 月 *Enter 99 if unknown*如果不知道输入99  8. Refused to answer拒绝回答  9. Don’t know 不知道 |  |
|  | | | |
| adult_2_59  成人_2_59 | Was the difficulty with swallowing with solids, liquids, or both?  吞咽困难是指吞咽固体食物，还是液体食物困难，或二者都有？ | 1. Solids 固体  2. Liquids 液体  3. Both 二者都有  8. Refused to answer拒绝回答  9. Don’t know 不知道 | ****  ****  ****  **** |
| adult_2_60  成人_2_60 | Did ______ have pain upon swallowing?  _____吞咽时是否伴有疼痛？ | 1. Yes 是  2. No否  8. Refused to answer拒绝回答  9. Don’t know 不知道 | ****  ****  **** |
| adult_2_61  成人_2_61 | Did _____ have belly pain?  _____是否有腹痛症状？ | 1. Yes 是  2. No否  8. Refused to answer拒绝回答  9. Don’t know 不知道 | ****  ****  **** |
|  | *If “No” or “Don’t know” or “Refused to answer” go to adult_2_64*  如果回答“否”、“不知道”、“拒绝回答”请转至“成人*_2_64”* | |  |
| adult_2_62  成人_2_62 | For how long before death did ______ have belly pain?  _____在去世之前，腹痛症状持续了多久？ | 1. __ __ hours 小时*Enter 99 if unknown*如果不知道输入99  2. __ __ days 天 *Enter 99 if unknown*如果不知道输入99  3. ___ __ month 月 *Enter 99 if unknown*如果不知道输入99  8. Refused to answer 拒绝回答  9. Don’t know不知道 |  |
| adult_2_63  成人_2_63 | Was the pain in the upper or lower belly?  疼痛部位在上腹或是下腹？ | 1. Upper belly 上腹部  2. Lower belly 下腹部  8. Refused to answer拒绝回答  9. Don’t know 不知道 | ****  ****  **** |
| adult_2_64  成人_2_64 | Did _____ have a more than usual protruding belly?  _____的腹部是否较以往凸出？ | 1. Yes 是  2. No否  8. Refused to answer拒绝回答  9. Don’t know 不知道 | ****  ****  **** |
|  | *If “No” or “Don’t know” or “Refused to answer” go to adult_2_67*  如果回答“否”、“不知道”、“拒绝回答”请转至“成人*_2_67”* | |  |
| adult_2_65  成人_2_65 | For how long before death did _____ have a protruding belly?  死前多长时间有_____腹部突出？ | 1. __ __ days 天 *Enter 99 if unknown*如果不知道输入99  2. __ __ month 月 *Enter 99 if unknown*如果不知道输入99  8. Refused to answer拒绝回答  9. Don’t know 不知道 |    |
| adult_2_66  成人_2_66 | How rapidly did _______ develop the protruding belly?  _____腹部凸出的速度如何？ | 1. Rapidly很快  2. Slowly很慢  8. Refused to answer拒绝回答  9. Don’t know 不知道 | ****  ****  **** |
| adult_2_67  成人_2_67 | Did _____ have any mass in the belly?  _____的腹内是否有任何肿块？ | 1. Yes 是  2. No否  8. Refused to answer拒绝回答  9. Don’t know 不知道 | ****  ****  **** |
|  | *If “No” or “Refused to answer” or “Don’t know”, go to question adult_2_72*  如果回答“否”、“不知道”、“拒绝回答”请转至“成人*_2_72”* | | |
| adult_2_68  成人_2_68 | For how long before death did ______ have a mass in the belly?  _____在去世之前，腹内肿块症状持续了多久？ | 1. __ __ days 天 *Enter 99 if unknown*如果不知道输入99  2. __ __ month 月 *Enter 99 if unknown*如果不知道输入99  8. Refused to answer拒绝回答  9. Don’t know 不知道 |  |
| adult_2_72  成人_2_72 | Did _____ have a stiff neck?  _____是否有颈部僵硬症状？ | 1. Yes 是  2. No否  8. Refused to answer拒绝回答  9. Don’t know 不知道 | ****  ****  **** |
|  | *If “No” or “Refused to answer” or “Don’t know” go to question adult_2_74*  如果回答“否”、“不知道”、“拒绝回答”请转至“成人*_2_74”* | | |
| adult_2_73  成人_2_73 | For how long before death did ______ have stiff neck  _____在去世之前，颈部僵硬症状持续了多久？ | 1. __ __ days 天 *Enter 99 if unknown*如果不知道输入99  2. __ __ month 月 *Enter 99 if unknown*如果不知道输入99  8. Refused to answer拒绝回答  9. Don’t know 不知道 |    |
| adult_2_74  成人_2_74 | Did ______ experience a period of loss of consciousness?  _____是否昏迷？ | 1. Yes 是  2. No否  8. Refused to answer拒绝回答  9. Don’t know 不知道 | ****  ****  ****  **** |
|  | *If “No” or “Refused to answer” or “Don’t know” go to question adult_2_82*  如果回答“否”、“不知道”、“拒绝回答”请转至“成人*_2_82”* | | |
| adult_2_75  成人_2_75 | Did the period of loss of consciousness start suddenly or slowly?  昏迷是突然发生还是缓慢发生？ | 1. Suddenly突然地  2. Slowly 很慢的  8. Refused to answer拒绝回答  9. Don’t know 不知道 |  |
| adult_2_77  成人_2_77 | Did it continue until death?  这种情况是否持续直至去世？ | 1. Yes 是  2. No否  8. Refused to answer拒绝回答  9. Don’t know 不知道 | ****  ****  **** |
| adult_2_82  成人_2_82 | Did _____ have convulsions?  (Demonstrate)  _____是否有抽搐症状？ | 1. Yes 是  2. No否  8. Refused to answer拒绝回答  9. Don’t know 不知道 | **** |
|  | *If “No” or “Refused to answer” or “Don’t know” go to question adult_2_85*  如果回答“否”、“不知道”、“拒绝回答”请转至“成人*_2_85”* | | |
| adult_2_83  成人_2_83 | For how long before death did the convulsions last?  在去世之前，抽搐症状持续了多久？ | 1. __ __ minutes 分钟 *Enter 99 if unknown*如果不知道输入99  2. __ __ hours 小时 *Enter 99 if unknown*如果不知道输入99  8. Refused to answer拒绝回答  9. Don’t know 不知道 |  |
| adult_2_84  成人_2_84 | Did the person become unconscious immediately after the convulsions?  ______是否在抽搐后随即陷入昏迷？ | 1. Yes 是  2. No否  8. Refused to answer拒绝回答  9. Don’t know 不知道 | ****  ****  **** |
| adult_2_85  成人_2_85 | Was _______ in any way paralyzed?  _____是否有任何瘫痪情况？ | 1. Yes 是  2. No否  8. Refused to answer拒绝回答  9. Don’t know 不知道 | ****  ****  ****  **** |
|  | *If “No” or “Refused to answer” or “Don’t know” go to section 3.*  如果回答“否”、“不知道”、“拒绝回答”请转至“第三部分” | | |
| adult_2_87  成人_2_87 | Which were the limbs or body parts paralyzed?  Read through the list in sequence and MARK ALL THAT APPLY  哪个肢体或身体部位瘫痪？（请按顺序阅读列表，并勾选所有适用项。） | 1. Right side (arm and leg)右侧（手臂和腿）  2. Left side (arm and leg) 左侧（手臂和腿）  3. Lower part of the body 身体下部  4. Upper part of the body 身体上部  5. One leg only 仅一条腿  6. One arm only 仅一只手臂  7. Whole body 整个身体  11. Other (specify ____________) 其他（特指____________）  8. Refused to answer拒绝回答  9. Don’t know 不知道 |  |

**If the deceased was female, then continue to Section 3: Questions for Women.**

**如果死者是女性，请继续转至第三部分：女性问题**

**If the deceased was male, then go to Section 4: Tobacco Use**

**如果死者是男性，请继续转至第四部分：烟草使用**

**SECTION 3 QUESTIONS FOR WOMEN 第三部分 女性问题**

| adult_3_1  成人_3_1 | Did ________ have any swelling or lump in the breast?  _____的乳房是否肿胀或有肿块？ | 1. Yes 是  2. No否  8. Refused to answer拒绝回答  9. Don’t know 不知道 | ****  ****  ****  **** |
| --- | --- | --- | --- |
| adult_3_2  成人_3_2 | Did ________ have any ulcers (pits) in the breast? Show photo  _____的乳房是否有任何溃疡（点状）症状？ | 1. Yes 是  2. No否  8. Refused to answer拒绝回答  9. Don’t know 不知道 | ****  ****  ****  **** |
|  | *Refer to gen_5_4: last known age of the deceased*适用于常规问题_5_4:死者最后知晓的年纪  *If the decedent is under 18 years old go to question adult_3_3a*如果死者小于18岁，转至成人_3_3a  *If the decedent is 18-39 years old go to question adult_3_5*如果死者年龄在18-39岁之间，转至adult_3_5  *If the decedent is over 40 years old go to question adult_3_3*如果死者年龄在大于40岁，转至adult_3_3 | | |
| adult_3_3a  成人_3_3a | Did ______ ever have a period or menstruate?  _____是否有过月经来潮？ | 1. Yes 是  2. No否  8. Refused to answer拒绝回答  9. Don’t know 不知道 | ****  ****  ****  **** |
|  | *If “Yes”, “Don’t know” or “Refused to answer” skip to adult_3_5*如果回答“是”、“不知道”、“拒绝回答”请转至“成人_3_5”  *If “No” skip to Section 4: Tobacco Use*如果回答“否”，转至第四部分：烟草使用 | | |
| adult_3_3  成人_3_3 | Had ________’s periods stopped naturally because of menopause?  _____是否因为更年期而自然绝经？ | 1. Yes 是  2. No否  8. Refused to answer拒绝回答  9. Don’t know 不知道 | ****  ****  **** |
|  | *If “No” skip to adult_3_5*  如果回答“否”，转至adult_3_5 | | |
| adult_3_4  成人_3_4 | Did _______ have vaginal bleeding after cessation of menstruation? (post-menopausal) _____在绝经后，是否有阴道出血症状？（绝经后） | 1. Yes 是  2. No否  8. Refused to answer拒绝回答  9. Don’t know 不知道 | ****  ****  ****  **** |
|  | *Skip to Section 4: Tobacco Use* |  |  |
| adult_3_5  成人_3_5 | Did ______ have vaginal bleeding other than her period? (intermenstrual) _____在月经结束后，是否有阴道出血症状？（经间期出血） | 1. Yes 是  2. No否  8. Refused to answer拒绝回答  9. Don’t know 不知道 | ****  ****  **** |
| adult_3_6  成人_3_6 | Was there excessive vaginal bleeding in the week prior to death?  在去世之前的当周内，阴道是否大量出血？ | 1. Yes 是  2. No否  8. Refused to answer拒绝回答  9. Don’t know 不知道 | ****  ****  **** |
|  | | | |
| adult_3_7  成人_3_7 | At the time of death was her period overdue?  在她去世之时，月经是否逾期没来？ | 1. Yes 是  2. No否  8. Refused to answer拒绝回答  9. Don’t know 不知道 | ****  ****  **** |
|  | *If “No” or “Refused to answer” or “Don’t know” go to question adult_3_10*  *如果回答“否”、“拒绝回答”或“不知道”，转至adult_3_10* | | |
| adult_3_8  成人_3_8 | For how many weeks was her period overdue?  她的月经逾期几周？ | 1. __ __ weeks 周 *Enter 99 if unknown*如果不知道输入99  8. Refused to answer 拒绝回答  9. Don’t know不知道 |  |
| adult_3_9  成人_3_9 | Did she have a sharp pain in the belly shortly before death? ______在去世之前不久，腹部是否有剧烈疼痛症状？ | 1. Yes 是  2. No否  8. Refused to answer拒绝回答  9. Don’t know 不知道 | ****  **** |
| adult_3_10  成人_3_10 | Was she pregnant at the time of death?  她在去世时，是否怀有身孕？ | 1. Yes 是  2. No否  8. Refused to answer拒绝回答  9. Don’t know 不知道 | ****  ****  ****  **** |
|  | *If “No” or “Refused to answer” or “Don’t know”, question adult_3_17*  *如果回答“否”、“拒绝回答”或“不知道”，转至adult_3_17* | | |
| adult_3_11  成人_3_10 | For how many months was she pregnant?  ______怀孕几个月？ | 1. __ __ months 月 *Enter 99 if unknown如果不知道输入99*  8. Refused to answer 拒绝回答  9. Don’t know不知道 |  |
| adult_3_12  成人_3_12 | Did ______ die during an abortion?  _____是否在流产期间去世？ | 1. Yes 是  2. No否  8. Refused to answer拒绝回答  9. Don’t know 不知道 | ****  ****  **** |
|  | *If “Yes”, skip to adult_3_19*  *如果回答“是”，转至成人_3_19* | | |
| adult_3_13  成人_3_13 | Did bleeding occur while she was pregnant?  在她怀孕期间，是否有流血症状？ | 1. Yes 是  2. No否  8. Refused to answer拒绝回答  9. Don’t know 不知道 | ****  ****  ****  **** |
| adult_3_14  成人_3_14 | Did she have excessive bleeding during labour or delivery?  在她临产或分娩期间，是否曾大出血？ | 1. Yes 是  2. No否  8. Refused to answer拒绝回答  9. Don’t know 不知道 | ****  **** |
| adult_3_15  成人_3_15 | Did she die during labor or delivery?  (“Labor” is the period of time by which contractions are less than 10 minutes apart.) 她是否在临产或分娩期间去世？ | 1. Yes 是  2. No否  8. Refused to answer拒绝回答  9. Don’t know 不知道 | ****  ****  ****  **** |
| adult_3_16  成人_3_16 | For how long was she in labor?  她的临产持续了多久？ | 1. __ __ hours 小时 *Enter 99 if unknown*如果不知道输入99  8. Refused to answer 拒绝回答  9. Don’t know不知道 |  |
|  | *If answer to adult_3_15 is “Yes”, skip to Section 4: Tobacco Use*  如果成人_3_15回答“是”，转至第四部分：烟草使用 | | |
| adult_3_17  成人_3_17 | Did she die within 6 weeks of having an abortion?  她是否在流产后 6 周内去世？ | 1. Yes 是  2. No否  8. Refused to answer拒绝回答  9. Don’t know 不知道 | ****  ****  **** |
|  | *If “Yes”, skip to adult_3_19* | | |
| adult_3_18  成人_3_18 | Did she die within 6 weeks of childbirth?  她是否在分娩后 6 周内去世？ | 1. Yes 是  2. No否  8. Refused to answer拒绝回答  9. Don’t know 不知道 | ****  ****  ****  **** |
|  | *If “No” or “Refused to answer” or “Don’t know”, skip to Section 4: Tobacco Use*  如果回答“否”、“拒绝回答”或“不知道”，转至第四部分：烟草使用 | | |
| adult_3_19  成人_3_19 | Did she have excessive bleeding after delivery or abortion?  在她分娩或流产后，是否曾大出血？ | 1. Yes 是  2. No否  8. Refused to answer拒绝回答  9. Don’t know 不知道 | ****  ****  **** |

**SECTION 4 TOBACCO USE 第四部分 烟草使用**

| adult_4_1  成人_4_1 | Did _________ use tobacco?  _____是否吸烟？ | 1. Yes 是  2. No否  8. Refused to answer拒绝回答  9. Don’t know 不知道 | ****  ****  ****  **** |
| --- | --- | --- | --- |
|  | *If “No” or “Refused to answer” or “Don’t know” go to Section 6: Health Records*  如果回答“否”、“拒绝回答”或“不知道”，转至第六部分：健康档案 | | |
| adult_4_2  成人_4_2 | What kind of tobacco did _____ use?  _____吸哪种类型的烟？ | \| 1. Cigarettes 香烟  2. Pipe 烟斗  3. Chewing Tobacco 咀嚼式烟草  4. Local form of Tobacco 当地的烟草形式  5. Other (specify ___________)其他（特指_____）  8.Refused to answer拒绝回答  9. Don’t know 不知道 \| \| --- \| |  |
|  | *If “Yes” to cigarettes, continue to adult_4_4. If “No” to cigarettes, go to Section 6: Health Records* 如果香烟问题回答“是”，继续进入成人_4_4。如果香烟问题回答“否”，进入第六部分：健康档案 | | |
| adult_4_4  成人_4_4 | How many cigarettes did _____ smoke daily?  _____每日吸多少支香烟？ | 1. Number ___ ___数量___ ___  8. Refused to answer拒绝回答  9. Don’t know不知道 |  |

**SECTION 6 HEALTH RECORDS 第六部分 健康档案**

| adult_6_1  成人_6_1 | Was care sought outside the home while the deceased had this illness?  _____在患此病时，是否曾寻求家庭以外的护理？ | 1. Yes 是  2. No否  8. Refused to answer拒绝回答  9. Don’t know 不知道 |        |
| --- | --- | --- | --- |
|  | *If “No” or “Don’t know” or “Refused to answer” go to adult_6_3a*  如果回答“否”、“不知道”或“拒绝回答”，转至成人_6_3a | | |
| adult_6_2  成人_6_2 | Where or from whom did you seek care?  (CHECK ALL THAT APPLY)  您从何处寻求护理或接受谁的护理？（勾选所有适用项） | Traditional Healer传统治疗  Homeopath顺势疗法  Religious leader宗教领袖  Government Hospital 政府医院  Governmental health center or clinic 政府的卫生中心或诊所  Private Hospital 私立医院  Community-based practitioner associated with health system 社区卫生服务机构  Trained birth attendant 受过训练的助产士  Private physician 私人医生  Pharmacy, drug seller, store, market 药房、医药商店或市场  Other provider 其他提供者  Relative, friend (outside household) 相关的，朋友）  88. Refused to answer 拒绝回答  99. Don’t know 不知道 |                            |
| adult_6_3  成人_6_3 | *Record the name and address of the hospital, health center or clinic where the care was sought. :*  记录患者寻求护理之任何医院、卫生中心或诊所的名称和地址： |  |  |
| adult_6_3a  成人_6_3a | Did a health care worker tell you the cause of death?  您是否由卫生保健工作者告知过死者的死因？ | 1. Yes 是  2. No否  8. Refused to answer拒绝回答  9. Don’t know 不知道 |        |
|  | *If “No” or “Don’t know” or “Refused to answer” go to adult_6_4*  如果回答“否”、“不知道”或“拒绝回答”，转至成人_6_4 | | |
| adult_6_3b  成人_6_3b | What did the health care worker say?  该工作人员对您是如何叙述的？ |  |  |
| adult_6_4  成人_6_4 | Do you have any health records that belonged to the deceased?  您是否持有属于死者的任何健康记录？（例如，病历本或处方单） | 1. Yes 是  2. No否  8. Refused to answer拒绝回答  9. Don’t know 不知道 |        |
|  | *If “No” or “Don’t know” or “Refused to answer” go to adult_6_9*  如果回答“否”、“不知道”或“拒绝回答”，转至成人_6_9 | | |
| adult_6_5  成人_6_4 | Can I see the health records?  我能否查看这些健康记录？（例如，病历本或处方单） | 1. Yes 是  2. No否  8. Refused to answer拒绝回答  9. Don’t know 不知道 |        |
|  | *If “No” or “Don’t know” or “Refused to answer” go to adult_6_9. If “Yes”, and respondent allows you to see the records, transcribe all the entries*  如果回答“否”、“不知道”或“拒绝回答”，转至成人_6_9.如果回答“是”，并且允许你看档案，抄录所有填写的答案 | |  |
| adult_6_6  成人_6_6 | *Record the dates of the two most recent visits from the health record*  *If not listed, mark 9999*  是否可以获知最近两次看诊和最后一次病例填写的日期？（勾选所有适用项） | _ _/_ _/_ _ _ _  dd mm yyyy 天 月 年    _ _/_ _/_ _ _ _  dd mm yyyy 天 月 年 |  |
| adult_6_7  成人_6_7 | Record the date of the last note  Enter 9999 if unknown  输入最后一次病例填写日期 | _ _/_ _/_ _ _ _  dd mm yyyy 天 月 年 |  |
| adult_6_8  成人_6_8 | *Transcribe the note:*  抄录病例内容： |  |  |
| adult_6_9  成人_6_9 | Was a death certificate issued?  是否签发了死亡证明？ | 1. Yes  2. No  8. Refused to answer  9. Don’t know |        |
|  | *If “No” or “Don’t know” or “Refused to answer” go to Section 7: Open Ended Response*  如果回答“否”、“不知道”或“拒绝回答”，转至第七部分：开放式回答 | | |
| adult_6_10  成人_6_10 | Can I see the death certificate?  我能否查看死亡证明？ | 1. Yes 是  2. No否  8. Refused to answer拒绝回答 |    |
|  | *If “No” go to Section 7: Open Ended Response*  如果回答“否”，转至第七部分：开放式回答 | | |
| adult_6_11  成人_6_11 | *Record the immediate cause of death from the certificate.* 记录死亡证明中说明的直接死因 |  |  |
| adult_6_12  成人_6_12 | *Record the first underlying cause of death from the certificate.* 记录死亡证明中说明的第一根本死因。 |  |  |
| adult_6_13  成人_6_13 | *Record the second underlying cause of death from the certificate.* 记录死亡证明中说明的第二根本死因。 |  |  |
| adult_6_14  成人_6_14 | *Record the third underlying cause of death from the certificate.* 记录死亡证明中说明的第三根本死因。 |  |  |
| adult_6_15  成人_6_15 | *Record the contributing cause(s) of death from the certificate.* 记录死亡证明中说明的辅助死因。 |  |  |
|  | **END OF HEALTH RECORDS SECTION 健康档案部分结束**  **GO TO SECTION 7: OPEN ENDED RESPONSE AND INTERVIEWER COMMENTS/OBSERVATIONS**  **进入第七部分：开放式回答及访问者建议或观察** | | |

**Section 7 Open Ended Response and Interviewer Comments/Observations Section**

**第七部分 开放式回答及访问者建议或观察**

*Instructions to the interviewer: Say to the respondent: "Thank you for the patient responses to this exhaustive set of questions. Could you please summarize, or tell us in your own words, any additional information about the illness and/or death of your loved one?"*

访问者指南：告诉回答者“谢谢你对这么多的问题进行回答付出的耐心。你能总结一下或者用你自己的语言告诉我们，关于你挚爱的去世的家人的一些关于疾病其他的信息吗？”

*To the interviewer: Listen to what the respondent tells you in his/her own words. Do not prompt except for asking whether there was anything else after the respondent finishes. If the respondent mentions any of the following words, mark "mentioned". Tell the respondent to stop and start again if they mention a word of interest, so you have time to mark it down.*

针对访问者：仔细聆听回答者告知你的关于她/他的情况。不要诱导除了询问对方是否已经说完。如果受访者提及以下词语，请标记“提到”。当出现关键词语时可要求受访者暂停叙述以便你有时间将该词语记录下来。

**Adult Checklist成人清查清单**

| adult_7_1  成人_7_1 | **Key words关键词** | **Mentioned 提及** |
| --- | --- | --- |
|  | Chronic Kidney Disease 慢性肾病 |  |
|  | Dialysis 透析 |  |
|  | Fever 发烧 |  |
|  | Heart Attack (AMI) 心脏病发作（急性心肌梗死） |  |
|  | Heart Problems 心脏问题 |  |
|  | Jaundice (yellow skin or eyes) 黄疸（皮肤或眼部发黄） |  |
|  | Liver Failure 肝功能衰竭 |  |
|  | Malaria 疟疾 |  |
|  | Pneumonia 肺炎 |  |
|  | Renal (Kidney) Failure 肾功能衰竭 |  |
|  | Suicide 自杀 |  |

| adult_7_99  成人_7_1 | Confirm that no words of interest were used during the open response.  确认在受访者叙述过程中没有出现以上关键词 | 1. No word was mentioned 没有提及词语  9. Don’t know 不知道 |  |
| --- | --- | --- | --- |

**END OF INTERVIEW 访问结束**

**THANK RESPONDENT FOR PARTICIPATION谢谢受访者参与**

## 9.6 Appendix A-6. Interview guide for process evaluation

**Interview guide for patients**

Thank you for participating the SINEMA study in the past months. The purpose of this interview is to seek your feedback and suggestions on SINEMA intervention model that you have been involved in the past months. Your comments are appreciated and will help us improve the SINMEA intervention model to benefit more stroke patients in rural China.

The interview will last for about 30 minutes and will be recorded if consent is provided.

**Step 1**: Casually chat with the participant to establish rapport

**Step 2**: Ask the questions below:

Question 1: basic information

- When is the onset of the stroke? Any recurrence?
- What is your main problem or concern after getting the stroke?
- Who are the main person who can give you support (healthcare, mental support, etc.)?

Question 2: follow-up visit by village doctors

- How often did you visit your village doctor before the study?
- What did your village doctor do when you visit him/her (measure your blood pressure, ask questions about medication use, prescribe new medicine, etc.)?
- Compared to previous doctor visit, did you spend more time to talk to your doctor than before?
- Do you think the APP helped the village doctor? Do you have any suggestions to them?
- Is it necessary that the village doctors follow-up you monthly and last for a whole year?

Question 3: take medicines

- Which medicines are you taking? How many time do you take every day?
- Did you take medicines everyday according to the medicine prescription by your village doctors? Any trouble in taking medicines? (forgot, feel better and stop, etc.)
- Did you use the calendar to record the date that you take the medicines? Any better ways?

Question 4: Text and voice messages

- Do you share phone with your family member? Do you use the phone everyday?
- Did you receive text messages (how many, when)?
- Do you have difficulties in reading them? What information did you learn from the text messages? Did you try to follow the suggestions from the text messages to change your lifestyles (eg. stop smoking, healthy eating, adhere to medication use, doing physical activities, etc)?
- Did you receive voice messages (how many, when)?
- Do you have difficulties in listening them? What information did you learn from the text messages? Did you try to follow the suggestions from the text messages to change your lifestyles?
- In general, which way do you prefer? Do you think these messages could help you to change your lifestyle behaviour?

Question 5: Other comments and suggestions?

**Step 3**: Thank the interviewee for their time and hand over small gift.

**Interview guide for Village doctors, township and county managers**

Thank you for participating the SINEMA study in the past months. The purpose of this interview is to seek your feedback and suggestions on SINEMA intervention model that you have been involved in the past months and on SINEMA application that you have used in the past months. Your comments are appreciated and will help us improve the SINMEA intervention model and the SINEMA application to benefit more stroke patients in rural China.

The interview will last for about 30 minutes and will be recorded if consent is provided.

**Step 1**: Casually chat with the participant to establish rapport

**Step 2**: Ask the questions below:

Questions 1: stroke management in China

- What are the main difficulties of prevention and management of stroke in primary settings?
- Can the national basic public health service meet the needs of stroke secondary prevention?
- Compared with the current national public health service system (paper + online system), what are the advantages and disadvantages of the APP?

Question 2: Training

- Do you think your medical knowledge has been improved after the training? In what way?
- Which part of the training do you like best? Which part of the training is unnecessary and should be deleted?
- Is there any information you think is important but has not been covered in our training? Is there any information that you think is too easy for you and do not need to be trained?
- Is the training on APP use enough? Will you use the training section on the APP?
- Any other suggestions?

Question 3: Follow-up visit

- How did you follow-up the patients involved in this study in your village? In the clinics or at patients’ home?
- How did you usually your work and our follow-ups? Did you follow-up them all on the same day or on different days?
- How do you think of the workload (high, medium, low)? Can you keep conducting the one-year intervention on 25 patients?
- On average, how long did you use in following up one patient? Did you find any difficulties in following up patients?
- Do you think your patients followed your suggestions on medication use? How did you evaluate their medication adherence? Did they record their medication use every day?
- How did you measure their blood pressure? How many times?

Question 4: SINEMA APP

- Is it difficult to learn to use the APP?
- Does the APP help you better manage your patients or you think the APP burdened you heavily?
- Did you use your own SIM card or WIFI?
- Any suggestions on improving the design of the APP?

Question 5: Key performance indicators, compensations and management

- Any better key performance indicators?
- Do you think the salary we give to you is equivalent to your expectation?
- How do you evaluate the manger in township hospital? Do you think they provide enough support for you?
- Do you this the Wechat group is useful?

Question 6: Other comments and suggestions?

**Step 3**: Thank the interviewee for their time and hand over small gift.

**Part B: Protocol for the long-term observational follow-up of the SINEMA trial**

**Version Date: August 10th, 2022**

# **1. AIMS**

To investigate the long-term effectiveness of the one-year SINEMA intervention on major outcomes:

- To conduct follow-up assessment of surviving participants and investigate the long-term effects of the SINEMA intervention on primary outcomes (blood pressure) and secondary outcomes (medication use and adherence, stroke recurrence, hospitalization, disability, and mortality).
- To collect detailed information about vital status of deceased participants and analyze the effect of the SINEMA intervention on mortality.

# **2. ETHICS REVIEW AND HUMAN SUBJECT PROTECTION**

## 2.1 Ethical review

The study was approved by the ethical boards of Chinese Academy of Medical Sciences & Peking Union Medical College (CAMS & PUMC-IEC-2022-062) and the Duke University (Pro00082130-AMD-9.1). All participants will provide written informed consent.

## 2.2 Participant informed consent

All participants will provide written informed consent.

#### Written informed consent from stroke patients participating the study

A written informed consent will be obtained from patients (**Appendix B-1**). Before the recruitment, village doctors will introduce the study to all prospective participants and invite them to participate in the study. On the day of recruitment, the trained research staffs will present the information to all prospective participants. Then, a one-on-one meeting will be organized between prospective participants and research staffs in a private space. Research staffs will address questions and concerns that prospective participants have and then ask their decision. The overall consent discussion will take about 10-15 minutes. Patients will have a copy of the form with contact information.

#### Written informed consent for village doctors participating the study

A written informed consent will be obtained from village doctors (VDs) before the interview (**Appendix B-1**). The trained research staffs will conduct the consent process at a private place. The research staffs will present the information on the consent document and answer questions from the interviewees. The overall consent discussion may take about 10-15 minutes. Interviewees will have a copy of the informed consent form with the contact information.

#### Written informed consent from the family member for participating the verbal autopsy interview

A written informed consent will be also obtained from the family member before the verbal autopsy interview (**Appendix B-1**). The trained research staffs will contact the interviewees in advance and conduct the consent process at a private place. The overall consent discussion may take about 20-30 minutes. Interviewees will have a copy of the informed consent form with the contact information.

# **3. STUDY DESIGN**

## 3.1 Study design, setting, and participants

This study is a 4.5-year post-trial follow-up of the SINEMA trial, a community-based, open-label, two arm, cluster-randomized controlled trial conducted in fifty villages in rural Hebei, China. The detailed trial design has been described previously.^67^ Briefly, eligible participants were community-dwelling adults living in the recruited villages with a history of stroke diagnosed at the county- or higher-level hospitals and in a clinically stable condition with at least basic communication abilities. Individuals were excluded if they were bedridden, had severe life-threatening diseases with an expected life span shorter than 6 months, and declined consent. Recruitment took place between June and July 2017.

Randomization, performed by a biostatistician unaware of the clusters by using a computer-generated random numbering system, allocated the fifty villages in a 1:1 ratio with stratification by township to either the SINEMA intervention or control arm. A follow-up assessment was conducted by blinded assessors in July 2018, where 1266 surviving participants completed the assessment.

The active phase of the intervention spanned from July 2017 to July 2018, post which the intervention concluded, though village doctors could optionally continue offering these services without financial incentive or further project support.

## 3.2 Procedures of follow-up assessment among participants

All surviving participants, regardless of their follow-up status at 12 months, of the SINEMA trial will be invited to participate in a 5-year follow-up.

Information on the vital status of the participants will be provided by village doctors, which enabled the generation of a list of survival participants who are eligible to participate in the survey and a list of deceased participants whose vital status, date and cause of deaths will be further collected.

### 3.2.1 Follow-up assessment for survived participants

For all survived participants, they will be contacted by corresponding VDs to inform them about the study. They will be invited to come to the corresponding village clinics to complete the post-trial assessment. The post-trial assessment for survivors will be performed by blinded outcome assessors, who are staff members from Centers for Disease Control (CDC) in a nearby county and received training for assessment. These assessors will follow the standard protocol to measure outcomes uniformly across all villages and participants, consistent with previous assessment at baseline and 12 months. The assessment consists of surveys will be administered through face-to-face interviews (**Appendix B-2**) with responses will be recorded in an online survey platform (Qualtrics, Provo, UT), and standard anthropometric measures.

### 3.2.2 Data collection for deceased participants

For deceased participants, we will employ a multifaceted approach to collect information on the date and cause of death from three major sources to gain the most reliable information. Firstly, we will utilize participants' unique ID numbers to link them to the local Center for Disease Control and integrate this data with the local death registration system. Secondly, to account for potential delays in the death records' updates, we will also independently collect vital status information through a standardized Excel form, including death dates and causes, from village doctors for all 50 villages. This information will supplement the CDC data. Thirdly, when there is key information missing or major inconsistency between two available sources, we will conduct a smartphone-based shortened version of verbal autopsy (VA) survey to informative family members of some selected dead participants by using Population Health Metrics Consortium (PHMRC) Shortened Questionnaire adult module. Trained health workers will conduct the VA interview. The procedure for the verbal autopsy survey will remain consistent with the one-year follow-up, with trained data collector administering a shortened version of the survey via smartphone to family members of deceased participants.

### 3.2.3 Strategies to improve follow-up rate

To improve the follow-up rate, the following strategies will be performed: Participants whose village doctors informed that they have moved outside of the village, the last known residence will be contacted to provide the address and contact information of the participants if possible.

1. For participants who have relocated to other villages within the Nanhe county and in villages where the survey is planned, participants will be invited to participate in the survey of the corresponding villages.
2. For participants who have relocated within the county but in the area that were not involved in the SINEMA study, a team of researcher will contact them and schedule a door-to-door survey for follow-up assessment.
3. For participants who have relocated outside of the county, the research team and the data collection team will discuss the feasibility of performing data collection outside of the county.
4. Participants will be considered as lost to follow-up when known alive, but the participants were not able to complete the assessment following the previous mentioned procedure or refuse to participate in the study.

For patients with mental health issues or other severe diseases that may present difficulties for them to complete the survey, the data collection will be conducted with accompany of family member if possible. Family members may provide proxy responses to certain questions and the data collectors will label proxy responses where appropriate.

## 3.3 Outcomes evaluation and measurement

The outcomes for follow-up assessment will be in the same way of the baseline and 12-month follow-up assessment. Table B-1 illustrate all the key aspects of measurement by comparing with baseline and 12-month follow-up assessment.

### 3.3.1 Primary outcomes

#### Systolic blood pressure

Systolic BP, the primary outcome of the SINEMA trial, is also the primary outcome of interests for this post-trial follow-up assessment. Systolic BP, will be analyzed as the differences between arms in the change of BP from baseline to 4-year post-trial. In line with previous assessments,^8^ BP will be measured on the right upper arm while the participants are seated, following 5 minutes of quiet rest, using an electronic BP monitor (Omron HEM7052). Two measurements will be obtained, with the mean value calculated. If the difference between the first two systolic BP readings exceeds 10 mmHg, a third measurement will be taken and the last two readings will be used for calculation.

#### Mortality

Mortality, an exploratory outcome of the original trial, will be considered as a key primary outcome of interests for the follow-up assessment considering the relatively long follow-up period. Date and cause of deaths will be collected from multiple approaches as mentioned above.

### 3.3.2 Secondary outcomes

The secondary outcomes of interests will be in line with the original protocol, including diastolic blood pressure, medication adherence (measured by using 4-item Morisky Green Levine Scale), physical activity level (measured by using IPAQ-short form), mobility (measured by using Timed-up and go test), health-related quality of life (measured by using EQ-5D-5L), disability (measured by using modified Rankin Scale), stroke recurrence (measured by self-reported experience of stroke recurrence) and stroke-related hospitalization (measured by self-reported history of hospitalization).

### 3.3.3 Additional measurement in follow-up assessment

The follow-up assessment will add a few measurements that were not included in the original trial, but could be important for assessing the health status of individuals living with stroke.

1. *Cognitive function*

It has been confirmed that stroke could result in the cognitive impairment. Many stroke patients suffer cognitive decline after stroke. We will measure the cognitive function by using The Community Screening Instrument for Dementia (CSI-D).^68^ The Chinese version of CSI-D has been validated to be a reliable and educationally independent screening instrument for dementia sensitive to detect early stage of the disease.^69^

1. *Functional dependence*

Many stroke patients suffer functional dependence post stroke. Functional independence is a key factor in stroke rehabilitation. We will measure the functional dependence using activities of daily living (ADL) and instrumental activities of daily living (IADL) scales. ADL and IADL are considered a primary functional status measure in stroke rehabilitation because of their relative objectivity, simplicity, and relevance to patients.^70^

1. Grip strength test

We also performed grip strength tests for both hands among participants. The measurement was taken by using grip strength tools (YuejianTM WL-1000, Nantong, China), which has been used in other studies previously as a standardized tool for grip strength assessment.

**Table B-1. Schedule of assessments of SINEMA post-trial study**

| **Aspect of data collection** | **Baseline** | **1-year follow-up** | **Post-trial follow-up** |
| --- | --- | --- | --- |
| **Questionnaire** |  |  |  |
| Demographics | √ | √ | √ |
| Family information | √ | √ | √ |
| Lifestyle risk factors | √ | √ | √ |
| Disease history | √ | √ | √ |
| Cognitive function (CSI-D) |  |  | √ |
| Disability (modified Rankin scale) | √ | √ | √ |
| Functional dependence (ADL) |  |  | √ |
| Health-related Quality of life (EQ-5D-5L) | √ | √ | √ |
| Depression (PHQ-2 and PHQ-9) | √ | √ | √ |
| Medication adherence (4 item4-item Morisky Green Levine Scale) | √ | √ | √ |
| **Physical measurement** |  |  |  |
| Time up and go | √ | √ | √ |
| Height | √ | √ | √ |
| Weight | √ | √ | √ |
| Waist circumference | √ | √ | √ |
| Neck circumference |  |  | √ |
| Grip strength |  |  | √ |
| Blood pressure | √ | √ | √ |

## 3.4 Statistical analysis

### 3.4.1 General analysis

The analysis for major outcomes will be in the same way in line with the 12-month follow-up assessment, according to the published statistical analysis plan. The intention-to-treat principle will be used for all analyses. Descriptive analysis will be performed with means and standard deviations (SDs) or medians and quartiles for continuous variables, and counts and percentages for binary variables. The analysis of all continuous or binary outcomes, apart from mortality, remains consistent with the one-year follow-up statistical analysis plan. Specifically, for continuous variables, we will use mixed-effects linear regression to estimate differences between two arms in the outcomes from baseline to 12 months and 5.5-year post-baseline, with random intercept for village (cluster) and fixed effect for township. The main model will adjust for age, sex, and baseline outcome, and removed outliers based on an a priori decision to exclude values more than two interquartile ranges above the third quartile or below the first quartile of the distribution.^71^ In instances where the mixed effects model did not converge, we will employ the generalized estimating equations method by fitting a Gaussian model with an identity link. For binary outcomes, generalized estimating equations approach will be adopted to obtain population-average treatment effect and similar adjustment on township, age, sex, and baseline outcome was made. We will use a Poisson model with log link and robust standard errors to obtain risk ratios and with identity link to obtain risk differences.^62,72^ If models did not converge, we will use a marginal standardization approach by fitting a binomial model with a logit link to obtain risk differences and risk ratios.^72^

### 3.4.2 Analysis of mortality

The follow-up period for mortality is from date of baseline to date of death, or the date of follow-up assessment, whichever came first. For missing information on date of death, we will use the following strategies: (1) For participants who died during the baseline and 12-month follow-up, we impute the date using the median survival time of deceased participants at 12-months; (2) For participants who died during the 12-month follow-up and post-trial follow-up, we will impute the date using the median survival time of deceased participants who died during the post-trial period.

The cause of deaths will be classified by adopting the classification for cardiovascular mortality and stroke-related mortality used by the Global Burden of Diseases, Injuries, and Risk Factors Study. For missing information on cause of death, deaths will classified as other causes of deaths.

Mortality rate for each mortality outcome will be estimated as the number of events divided by the total person-months since baseline. We will assess cumulative incidence of all mortality outcomes with the Kaplan-Meier curves, while accounting for village as clustering. Cox proportional hazards models using the generalized estimating equation (GEE) approach with an independence working correlation and robust sandwich variance will be used to compute hazard ratios (HRs) and 95% confidence interval (95% CIs) for the analysis with events and date of death as dependent variable. Age, sex, and township will be adjusted when estimating HRs. We will test adherence to proportional hazards assumption by plotting smoothed Schoenfeld residuals.

### 3.4.3 Subgroup analyses

The subgroup analysis will be conducted by following the published statistical analysis plan to include subgroup analysis on gender (male/female), age group (<65 vs. >=65), education level (schooling vs. no schooling), type of stroke (hemorrhage vs. ischemic stroke), years since last stroke events (less than 3 years vs. more than 3 years), and having benefit package for Chronic Diseases Outpatients (have benefit package vs. does not have the benefit package), Patient Health Questionnaire (PHQ-2) score (0-2 vs. 3-6). We will also conduct additional subgroups by marital status (married vs. widowed, divorced, or not married), annual household income (<5,000 vs. ≥5,000), years since the first stroke events (less than 5 years vs. more than 5 years), disability status (moderate to severe disability vs. no to slight disability). These subgroup analyses will be performed by adding to the model both the variable and its interaction term with treatment arm. We caution that these analyses may be not powered.

For mortality analysis, subgroup analyses will be conducted following the subgroups prespecified for the one-year outcome analysis. Additional subgroup analyses by considering the baseline status of annual income (<5,000 vs. ≥5,000), marital status (married vs. widowed, divorced, or not married), systolic blood pressure (≥ 140 mm Hg vs. < 140 mm Hg), and disability status (moderate to severe disability vs. non-disability to slight disability) will also be conducted. Subgroup variable and its interaction term with the intervention were added to the minimally adjusted model used in the primary analysis as fixed effects in the subgroup analysis.

### 3.4.4 Sensitivity analyses

Following the previous statistical analysis plan^71^, sensitivity analyses will be performed involved additional adjustment for covariates that were statistically imbalanced between arms at baseline, associated with the status of loss to follow-up, or both, as well as include all outliers.

3.5 Data collection among village doctors

In addition to patient surveys, we will collect some basic information on all VDs in 50 villages who were part of the study. The survey will be designed on the basis of the survey from baseline and 12-month follow-up and a link of the survey will be sent to all VDs. The survey includes questions about their demographic information, history of medical training or education, current occupation, etc. They will also be asked about their knowledge, attitude and behaviors regarding the secondary prevention of stroke. This present follow-up for VDs is consistent with the procedure in previous protocol.

### 3.5 Qualitative assessment

Semi-structured in-depth interviews will be conducted for post-trial (**Appendix B-3**). The interviews would focus on how the SINEMA intervention effects lasted for long-term, and the potential enablers and barriers that may influence the sustainability. And we would explore the intervention components which got maintenance and may explain the mechanism of the sustainability of SINEMA intervention. The interviews will be conducted by the research teams. We will invite participants and stakeholders (including patients who suffered stroke, village doctors, physicians at township hospitals, and a county coordinator) to participate in the interviews.

We will use purposive sampling to ensure the diversity of the participants and the coverage across villages and townships. The research team will identify the villages to ensure that at least one village from each township would be selected. All intervention villages will be covered. Within each village, the research team will interview the village doctor and selected one or two participants based on their availability, willingness, ability to communicate, and demographic characteristics to ensure representatives of the participants. Interviews will be conducted either in the village clinics or at the homes of the participants. The physicians at township hospitals and the county coordinator who involved in the study will also be interviewed at their working places. All interviews will last between 20 and 40 min and will be audio-recorded with verbatim transcripts for data analysis.

# **4.** **QUALITY CONTROL AND PROJECT MANAGEMENT**

## 4.1 Pilot study

To test the feasibility, reliability, and validity of the updated questionnaire and its procedure before the main trial, we will choose about 10 participants from one village to conduct the pilot testing of the questionnaire and procedure of data collection.

## 4.2 Project Management

The follow-up assessment was conducted under the management of the teams at Duke Kunshan University and Peking Union Medical College. The research teams will have weekly meetings to ensure the project will be ongoing as planned. The data collection will be administered with collaboration of Center of Disease Prevention and Control at Nanhe County and Ren County. To ensure the quality of assessment, a project coordinator will work with the data collector team.

The international steering committee and data management committee will continue their role in supervise and auditing the project.

# **5. APPENDIX**

## 5.1 Appendix B-1. Informed Consent Forms

### SINEMA long-term follow-up consent form for stroke patients

**Project Concise Summary**

**(Stroke Patients)**

The purpose of this research study is to determine the long-term effectiveness of the previous SINEMA trial through a new round of follow-up survey. Participation in research studies is voluntary. In this study, participants will complete the structured questionnaires (30-40 minutes) and receive the well-designed physical measurement (5-10 minutes). Data on stroke recurrence, hospitalization, and medical costs will be collected from county hospital inpatient medical records and CDC registration system.

Your participation in the research will lead to minimum risks, known and anticipated. In addition, the existing medical services you are receiving will not be affected by your participation in this research. You will not get any direct benefit by participating this study. However, you will be aware of your blood pressure level and other health related measurements (weight, height, etc.) through the survey process.

If you are interested in learning more about this study, please continue reading below.

You are being asked to take part in this research study because you have (***finished the SINEMA 1-year intervention***). Research studies are voluntary and include only people who choose to take part. Please read this consent form carefully and take your time making your decision. As your study staff discusses this consent form with you, please ask him/her to explain any words or information that you do not clearly understand. We encourage you to talk with your family and friends before you decide to take part in this research study. The nature of the study, risks, inconveniences, discomforts, and other important information about the study are listed below. Please tell the study doctor or study staff if you are taking part in another research study.

**Why is this study being done?**

Stroke is the leading cause of death and disability among adults in rural China. The recurrent rate of stroke is relatively high in China, while secondary prevention and management for stroke can effectively prevent disease recurrence. However, the secondary prevention after the acute phase of stroke is relatively lacking or far below the standards of clinical guidelines in China.

From 2017 to 2018, you have participated in SINEMA project and completed the baseline and 1-year follow-up surveys. This study is the 5-year follow-up of the SINEMA project. It will determine the long-term effectiveness of the SINEMA intervention for stroke prevention in rural areas in China, as well as the factors that affect the long-term prognosis of stroke patients.

**How many people will take part in this study?**

At most 1,269 people will take part in this study.

**What is involved in the study?**

If you agree to participate in this study, you will need to complete:

1) a questionnaire survey (30-40 minutes) and the physical measurement (about 5-10 minutes) conducted by trained surveyors. The content of the questionnaire includes information including your demographic information, life style and health behavior, disease history, medication and adherence of medication, disability and dependence, health status and self-reported health, cognitive function and medication cost. You will also have a physical examination comprising of assessment of blood pressure, weight, height, waist circumference, neck circumference, grip strength and the "Timed Up and Go" test. Most of the components of the questionnaire and measurements will be identical with the follow-up survey four years ago. We also plan to conduct follow-up surveys every 1-5 years from now on. Your consent will allow us to include you in future follow up studies. You always have the right to decline at any time without any impact on your medical services or other benefits and rights.

2) After getting your approval, we will collect data about your medical expenditure through county hospital inpatient medical records and CDC registration system from September 1^st^, 2018 to September 30^th^, 2022.

**How long will be in this this study?**

The study will last from 35-50 minutes.

**What are the risks of the study?**

Your participation in the research will lead to minimum risks, known and anticipated. In addition, the existing medical services you are receiving will not be affected by your participation in this research. There are no physical risks associated with this study. There is, however, the potential risk of loss of confidentiality. Every effort will be made to keep your information confidential.

**Are there benefits to taking part in the study?**

You will not get any direct benefit by participating this study. However, you will be aware of your blood pressure level and other health related measurements (weight, height, etc.) through the survey process.

**Will my information be kept confidential?**

Participation in research involves some loss of privacy. We will do our best to make sure that information about you is kept confidential. No one outside our research team including your family members, neighbors and the local health workers will have access to your personal information. All the data will be stored securely on password protected computers and servers. Your name and other identifying information will not be linked with the responses. These data records will be completely deleted three years after the end of the project. Any reportage or presentation of research results will be in the aggregate, and your information will not be identifiable. We will share only the minimum necessary information in order to conduct the research.

While the information and data resulting from this study may be presented at scientific meetings or published in a scientific journal, your name or other personal information will not be revealed.

**What ere the costs to you?**

There is no fee for your participation in this research.

**What about compensation?**

You will not be paid for your participation in this research. Rather, we will give a small gift equivalent to 5-10 RMB (1-1.5 USD) at the time of your enrollment.

**What about my rights to decline participation or withdraw from the study?**

You may choose not to be in the study, or, if you agree to be in the study, you may withdraw from the study at any time. If you withdraw from the study, no new data about you will be collected for study purposes other than data needed to keep track of your withdrawal.

A description of this study will be available on *https://clinicaltrials.gov/* as required by U.S. Law. This Web site will not include information that can identify you. At most, the Web site will include a summary of the results. You can search this Web site at any time.

Your data may be stored and shared for future research without additional informed consent if identifiable private information, such as your name and medical record number, are removed. If your identifying information is removed from your data, we will no longer be able to identify and destroy them.

The use of your data may result in commercial profit. You will not be compensated for the use of your data other than what is described in this consent form.

**Whom do I call if I have questions or problems?**

For questions about the study or if you have problems, concerns, questions or suggestions about the research, contact Prof. ***Lijing L. Yan*** at ***(0512-36657057)*** during regular business hours.

For questions about your rights as a research participant, or to discuss problems, concerns or suggestions related to the research, or to obtain information or offer input about the research, contact the Duke University Health System Institutional Review Board (IRB) Office at (919) 668-5111.

**Statement of consent**

"The purpose of this study, procedures to be followed, risks and benefits have been explained to me. I have been allowed to ask questions, and my questions have been answered to my satisfaction. I have been told whom to contact if I have questions, to discuss problems, concerns, or suggestions related to the research, or to obtain information or offer input about the research. I have read this consent form and agree to be in this study, with the understanding that I may withdraw at any time. I have been told that I will be given a signed and dated copy of this consent form."

__________________________________________ ___________ ____________

Signature of Subject Date Time

__________________________________________ ___________ ____________

Signature of Person Obtaining Consent Date Time

__________________________________________ ___________ ____________

Signature of Accessing Information on Date Time

Hospital Medical Records and Medical Insurance

***(Optional)***

**____________________________________ _____________________** ____________

**Signature of Principal Investigator Date Time**

***(If applicable, add or substitute any of the following:)***

____________________________________ ___________ ____________

Signature of Legal Representative Date Time

_______________________________________

Relationship to Subject

Study PI：Lijing Yan

[lijing.yan@duke.edu](mailto:lijing.yan@duke.edu)

Duke Kunshan University

Tele: (0512) 36657057

Local Contractor: Mobai Hou

Nanhe County Department of health and family planning

Tele: (+86) 17732968998

Peking Union Medical College Medical Ethics Committee:

Tele: (010) 65105715

### SINEMA long-term follow-up consent form for village doctors

You are being asked to take part in this research study because you have (***participated in the previous SINEMA study***). Research studies are voluntary and include only people who choose to take part. Please read this consent form carefully and take your time making your decision. As your study doctor or study staff discusses this consent form with you, please ask him/her to explain any words or information that you do not clearly understand. We encourage you to talk with your family and friends before you decide to take part in this research study. The nature of the study, risks, inconveniences, discomforts, and other important information about the study are listed below. Please tell the study doctor or study staff if you are taking part in another research study.

**Why is this study being done?**

Stroke is the leading cause of death and disability among adults in rural China. The recurrent rate of stroke is relatively high in China, while secondary prevention and management for stroke can effectively prevent disease recurrence. However, the secondary prevention after the acute phase of stroke is relatively lacking or far below the standards of clinical guidelines in China.

From 2017 to 2018, you have participated in SINEMA project and completed the baseline and 1-year follow-up surveys. This study is the 5-year follow-up of the SINEMA project. It will determine the long-term effectiveness of the SINEMA intervention for stroke prevention in rural areas in China.

**How many people will take part in this study?**

Approximately 50 village doctors will take part in this study.

**What is involved in the study?**

If you agree to participate in this study, you will need to complete a survey includes questions about your demographic information, history of medical training or education, past and current occupation, etc. You will be asked about your knowledge, attitude and behaviors regarding the secondary prevention of stroke. You will be invited to have an interview with researchers to seek feedback. The interview will last for about 20-30 minutes. We will ask you questions about the following three aspects, and the interview will be audio-recorded:

1) Your experience in managing the stroke patients;

2) The sustainability for implementing the secondary management;

3) The reasons that you can or cannot be sustainable to implement the secondary prevention.

**How long will be in this this study?**

The study will last for 30-45 minutes.

**What are the risks of the study?**

We expected no to minimum risk for you to participate in this study. Your response will not be shared to any authorities and will not affect your career development in anyway. There is, however, the potential risk of loss of confidentiality. Every effort will be made to keep your information confidential.

**Are there benefits to taking part in the study?**

Taking part in this project will not bring you any direct benefits. But the result of this study may help us to understand the capacity of village doctors in providing the secondary prevention of stroke in resource-limited settings.

**Will my information be kept confidential?**

Participation in research involves some loss of privacy. We will do our best to make sure that information about you is kept confidential. No one outside our research team including your family members, neighbors and the local health workers will have access to your personal information. All the data will be stored securely on password protected computers and servers. Any reportage or presentation of research results will be in the aggregate, and your information will not be identifiable. We will share only the minimum necessary information in order to conduct the research.

While the information and data resulting from this study may be presented at scientific meetings or published in a scientific journal, your name or other personal information will not be revealed.

**What ere the costs to you?**

There is no fee for your participation in this research.

**What about compensation?**

You will not be paid for your participation in this research. Rather, we will give a gift equivalent to 50 RMB at the time of your enrollment.

**What about my rights to decline participation or withdraw from the study?**

You may choose not to be in the study, or, if you agree to be in the study, you may withdraw from the study at any time. If you withdraw from the study, no new data about you will be collected for study purposes other than data needed to keep track of your withdrawal.

A description of this study will be available on https://clinicaltrials.gov/ as required by U.S. Law. This Web site will not include information that can identify you. At most, the Web site will include a summary of the results. You can search this Web site at any time.

Your data may be stored and shared for future research without additional informed consent if identifiable private information, are removed. If your identifying information is removed from your data, we will no longer be able to identify and destroy them.

The use of your data may result in commercial profit. You will not be compensated for the use of your data other than what is described in this consent form.

**Whom do I call if I have questions or problems?**

For questions about the study or if you have problems, concerns, questions or suggestions about the research, contact Prof. ***Lijing L. Yan)*** at ***(0512-36657057)*** during regular business hours.

For questions about your rights as a research participant, or to discuss problems, concerns or suggestions related to the research, or to obtain information or offer input about the research, contact the Duke University Health System Institutional Review Board (IRB) Office at (919) 668-5111.

**Statement of consent**

"The purpose of this study, procedures to be followed, risks and benefits have been explained to me. I have been allowed to ask questions, and my questions have been answered to my satisfaction. I have been told whom to contact if I have questions, to discuss problems, concerns, or suggestions related to the research, or to obtain information or offer input about the research. I have read this consent form and agree to be in this study, with the understanding that I may withdraw at any time. I have been told that I will be given a signed and dated copy of this consent form."

__________________________________________ ___________ ____________

Signature of Subject Date Time

__________________________________________ ___________ ____________

Signature of Person Obtaining Consent Date Time

__________________________________________ ___________ ____________

Signature of Accessing Information on Date Time

Hospital Medical Records and Medical Insurance

***(Optional)***

**____________________________________ _____________________** ____________

**Signature of Principal Investigator Date Time**

***(If applicable, add or substitute any of the following:)***

__________________________________________ ___________ ____________

Signature of Legal Representative Date Time

__________________________________________

Relationship to Subject

Study PI：Lijing Yan

[lijing.yan@duke.edu](mailto:lijing.yan@duke.edu)

Duke Kunshan University

Tele: (0512) 36657057

Local Contractor: Mobai Hou

Nanhe County Department of health and family planning

Tele: (+86) 17732968998

Peking Union Medical College Medical Ethics Committee:

Tele: (010) 65105715

### SINEMA long-term follow-up consent form for verbal autopsy interview

Thank you and your family member for participating the RuSS project. We want to have an interview with you to collect information on cause of death of your family member.

I am very sorry to hear that a member of your household has passed away. Please accept my sympathies. For the purpose of assessing impacts of stroke on health outcome, we would like to collect further information on all deaths of participants of this project. I would like to invite you to participate in an interview as a main caretaker of [the deceased’s name].

If you agree to participate, we will interview you about the past health of the person in your family who recently died. The interview will last no more than 30 minutes and may be much shorter. We will be using a smartphone to record all information.

**Risks and Benefits**

Risk: Your participation in the research will lead to none to minimum risks. We will not share your response to any authorities.

Benefit: You will not get a direct benefit by participating in the interview.

**Payment**

You will not be paid for your participation in this interview.

**Confidentiality**

We will not link your name to your answers, so the information we learn will not be connected to you. Your responses will be treated in a confidential manner. No one outside our research team will have access to your personal information. All the information will be stored securely on password protected computers and servers. Any reportage or presentation of research results will be in the aggregate, and your information will not be identifiable.

**Withdrawal**

Participation in this interview is voluntary. You have the right to withdraw from it at any time without any fear of penalty or consequences. You are free to decide not to participate in the interview. We understand that you might be sad about your family member’s death and you may not want to talk about the person. Even if you agree at first to take part, you are free to change your mind at any time and to quit the interview. If you want to stop, you can tell the person asking you questions that you want to stop the interview. You will not suffer any penalty.

**Questions**

If you have any questions, please feel free to ask at any time. We will try our best to answer them.

**Authorization**

I have read (or someone explained) the information above and have had the opportunity to ask questions and receive answers.

**STATEMENT OF CONSENT**

"The purpose of this study, procedures to be followed, risks and benefits have been explained to me. I have been allowed to ask questions, and my questions have been answered to my satisfaction. I have been told whom to contact if I have questions, to discuss problems, concerns, or suggestions related to the research, or to obtain information or offer input about the research. I have read this consent form and agree to be in this study, with the understanding that I may withdraw at any time. I have been told that I will be given a signed and dated copy of this consent form."

__________________________________________ ___________ ____________

Signature of Subject Date Time

__________________________________________ ___________ ____________

Signature of Person Obtaining Consent Date Time

__________________________________________ ___________ ____________

Signature of Accessing Information on Date Time

Hospital Medical Records and Medical Insurance

***(Optional)***

**____________________________________ _____________________** ____________

**Signature of Principal Investigator Date Time**

***(If applicable, add or substitute any of the following:)***

__________________________________________ ___________ ____________

Signature of Legal Representative Date Time

__________________________________________

Relationship to Subject

Study PI：Lijing Yan

[lijing.yan@duke.edu](mailto:lijing.yan@duke.edu)

Duke Kunshan University

Tele: (0512) 36657057

Local Contractor: Mobai Hou

Nanhe County Department of health and family planning

Tele: (+86) 17732968998

Peking Union Medical College Medical Ethics Committee:

Tele: (010) 65105715

## 5.2 Appendix B2. Questionnaire of Patient Post-trial Follow-up

**A: BASIC INFORMATION**

A1. Date of interview: _(Month) / (Day)_

A2. Patient's initials (e.g. Jianguo Zhang, please fill in ZJG) _____

A3. Patient item number ☐ ☐ ☐ ☐ ☐ ☐ ☐

A4. Township ___________ Village ___________

A5. Patient's gender: ☐ Male ☐ Female

A6. Marital status: ☐ Married ☐ Unmarried ☐ Widowed ☐ Divorced ☐ Other

A7. Patient's medical insurance status

☐ Basic medical insurance for urban and rural residents of Xingtai (i.e. New Agricultural Cooperative)

☐ Outpatient special chronic disease management for urban and rural residents of Xingtai

☐ Other types of insurance (e.g. commercial insurance)

☐ Not covered by any insurance

A8. What is the main work you do now?

☐ Unable to work due to health reasons (including farm work and housework)

☐ Only light work (e.g. housework or manual work)

☐ Can do normal household work

☐ Can do simple farm work

☐ Can do heavy farm work

☐ Working outside the home and other physical work

☐ Business, self-employment

☐ Mental work (government workers/doctors/teachers/professionals)

☐ Other

**B: HOUSEHOLD/FAMILY INFORMATION**

B1. Do you have a mobile phone?

☐ Yes, a regular mobile phone (elderly)☐ Yes, a smartphone☐ No (go to B4)

B2. Do you share a mobile phone with your family?

☐ Yes☐ No

B3. How many people live with the patient (not counting the patient themselves, based on eating with the patient, including children over 6 years old, adolescents, and adults)? ________

B4. Do you have someone to take care of you during the day? (including reminding you to take your medication)

☐ Yes☐ No

B5. Who is your primary family caregiver?

☐ Spouse☐ Daughter☐ Son☐ Child's spouse☐ Grandchildren

☐ Grandchildren's spouse☐ Grandchildren and spouse☐ Siblings

☐ Parents ☐ Parents-in-law☐ Other

B6. How often does he/she care for you (including reminding you to take your medication)?

☐ Never (0 days per week)☐ Rarely (1-2 days per week)☐ Sometimes (3-4 days per week)

☐ Often (4-5 days per week)☐ Always (6-7 days per week)

B7. Do you need him/her to look after you?

☐ Not at all☐ Not very much☐ Fairly☐ More☐ Very much

B8. Do you feel that you need to be cared for by other people or in other ways?

☐ Very much☐ More than necessary☐ Fair☐ Less than necessary☐ Not at all

B9. For you and your partner, the amount of money you earn on your own in a year is: ________

☐ Don't know

B10. For you and your partner, the amount of social welfare income (retirement, pension, benefits, etc.) for one year is: ________

☐ Don't know

B11. For you and your partner, the amount of money given by your children in a year is: ________

☐ Don't know

Please write the patient item number again ☐ ☐ ☐ ☐ ☐ ☐

**C: SMOKING**

C1. Do you smoke (100 or more cigarettes in total)?

☐ Smoke (skip to C2)☐ Used to smoke, but don't anymore (skip to C3)

☐ Never smoked (skip to C4)

C2. How many cigarettes do you smoke on average in a day? (Please fill in the number) _______

C3. When you used to smoke, how many cigarettes did you smoke on average in a day? (Please fill in the number) _______

C4. In the past, how many years have you smoked in total? _______

C4a. In which year did you stop smoking? If you stopped smoking in 2015, please enter "2015": _______

C4b. Please enter the month in which you stopped smoking: _______

C4. Is there anyone in the household who smokes regularly?

☐ Yes☐ No

**D: EXERCISE**

D1. How many days in the past 7 days has the patient engaged in vigorous physical activity (e.g. carrying water, jogging, fast running, farming, fast cycling, etc.)?

Please consider only those physical activities that lasted at least 10 minutes at a time. ______ days

D2. On the days when there was vigorous physical activity as described above, how long was the patient usually able to do vigorous physical activity each day? ________ minutes

D3. How many days in the past 7 days has the patient engaged in moderate intensity physical activity (e.g. brisk walking, lifting light objects, cycling, jogging, etc.)?

Please consider only those physical activities that lasted at least 10 minutes at a time. ________ days

D4. On the days when moderate intensity physical activity as described above, how long was the patient usually able to do moderate intensity physical activity each day? ________ minutes

D5. How many days in the past 7 days has the patient walk for at least 10 minutes at a time on that day? (Includes walking at work, at home, from somewhere to somewhere, for recreation, for leisure) ________ days

D6. On the days when walking was available as described above, how long did the patient usually spend walking each day? ______ minutes

D7. How many hours did the patient sleep at night (not including time spent lying awake and not including daytime naps)? _____ hours

D8. How many hours in the past 7 days has the patient spend sitting or lying down approximately (not including sleeping at night)? _______ minutes

**E: DIET**

E1. How often do you eat vegetables on average?

☐ Every day (or >5 times a week)☐ 2-5 times a week☐ Once a week

☐ Less than once a week☐ Never or hardly ever

E2. How often do you eat fruit on average?

☐ Every day (or >5 times a week) ☐ 2-5 times a week ☐ Once a week

☐ Less than once a week ☐ Never or hardly ever

**F: FIRST BLOOD PRESSURE MEASUREMENT**

Measurement should do the following:

Step 1 Land on both feet

Step 2. Right arm measurement

Step 3. The upper arm and sphygmomanometer should be at the same level as the heart

Step 4. The center of the balloon is located right in the brachial artery

Step 5. Standard sleeves

Fa Please enter the value of the first blood pressure measurement for systolic pressure (high pressure): ________mmHg

Fb Please enter the value of the first blood pressure measurement for diastolic pressure (low pressure): ________mmHg

**H: HISTORY OF STROKE**

Only consider strokes diagnosed by county hospitals and above

H1. How many times have you been hospitalized for stroke in the last four months?

H2. Has there been a recurrence of stroke in the past year? (based on hospitalization, any level of hospitalization, only infusion without overnight stay in hospital does not count)

☐ Recurrence (skip to H2) ☐ No recurrence

H3. Number of recurrences of stroke: _______. For each recurrence, complete the following information.

|  | 1 | 2 | 3 | 4 | 5 |
| --- | --- | --- | --- | --- | --- |
| H4. Year |  |  |  |  |  |
| H5. Month |  |  |  |  |  |
| H6. Type of stroke |  |  |  |  |  |
| H7. Health facility attended |  |  |  |  |  |
| H8. Whether hospitalized for stroke |  |  |  |  |  |
| H9. Days of hospitalization |  |  |  |  |  |
| H10. Insurance reimbursement |  |  |  |  |  |
| G11. Out-of-pocket expenses |  |  |  |  |  |
| G12. Total expenses |  |  |  |  |  |

**I: HOSPITALIZATION FOR OTHER ILLNESSES**

I1. In the past year, have you been hospitalized for any reason other than a stroke? (only infusion without overnight stay in hospital does not count)

☐ Yes ☐ No

I2. In the past year, how many times have you been hospitalized for reasons other than stroke? ________

I3. In the past year, what was the total number of days spent in hospital for reasons other than stroke? ________

I4. In the past year, what were the reasons for hospitalization other than stroke? (If multiple hospitalizations, select all reasons for hospitalization)

☐ Heart disease (acute heart attack, angina, heart failure, coronary artery disease, etc.)

☐ Lung disease (acute pulmonary embolism, asthma, etc.)

☐ Blood system disease ☐ Kidney disease ☐ Tumour or cancer ☐ Trauma

☐ Other, please specify: ________

I5. Has the following been newly diagnosed in the past year at a county or higher hospital? (ask as fully as possible and tick all appropriate boxes)

☐ Hypertension☐ Dyslipidemia☐ Diabetes

☐ Serious heart disease (acute heart attack, angina, heart failure, coronary heart disease, etc.)

☐ Serious lung disease (e.g. acute pulmonary embolism, asthma, etc.)

☐ Tumor or cancer ☐ Hematological disorders ☐ Kidney disease

☐ Other diseases diagnosed at county level or above

☐ No diseases (other than stroke) diagnosed at county level or above

**J VILLAGE CLINIC (ASK ONLY ABOUT STROKE AS A MEDICAL CONDITION)**

J1. In the past 12 months, how often did you visit the village clinic because of stroke on average (including asking about your condition, taking blood pressure, getting medicine, controlling blood sugar; not including preventive fluids, seeing other illnesses such as cold, upset stomach, kidney disease, etc.)?

☐ Once a week or more (≥4 times a month)☐ 2-3 times a month☐ Once a month

☐ Once every 2 months (included) to once a month (not included)

☐ Once every 3 months (included) to once every 2 months (not included)

☐ Less than once every 3 months

J2. In the past month, how many times did you visit the village clinic because of stroke (including asking about your condition, taking blood pressure, getting medicine, controlling blood sugar; not including preventive fluids, seeing other illnesses such as cold, upset stomach, kidney disease, etc.)?

________ times

J3. In the past month, how many times did you have your blood pressure taken at the village clinic? ________ times

J4. In the past month, how many times did you have your blood pressure taken at home?

________ times

J5. In the past month, how many times have you had your blood pressure taken elsewhere than at the village clinic and at home?

________ times

**K: CSI-D**

K1. Now I am going to tell you three words and I would like you to repeat them after me

Boat House Fish

Repeat the three words, up to a maximum of six times. Or until the person has remembered them all correctly. Then say:

Very good, now try to remember these words because I will be asking you later

|  | **Correct** | **Incorrect** | **Can not tell** |
| --- | --- | --- | --- |
| (Interviewer points to their elbow)  K2. What do we call this? |  |  |  |
| K3. What do you do with a hammer?  Acceptable answer ‘To drive a nail into something’ |  |  |  |
| K4. Where is the local market/local store? |  |  |  |
| K5. What day of the week is it? |  |  |  |
| K6. What is the season? |  |  |  |
| K7. Please point first to the window and then to the door |  |  |  |
| K8. Do you remember the three words I told you a few minutes ago? |  |  |  |
| Boat |  |  |  |
| House |  |  |  |
| Fish |  |  |  |

**L: MODIFIED RANKIN SCALE**

J1 Patient's condition (single choice)

☐ No symptoms at all

☐ No significant disability despite symptoms; able to carry out all usual duties and activities

☐ Slight disability; unable to carry out all previous activities, but able to look after own affairs without assistance

☐ Moderate disability; requiring some help, but able to walk without assistance

☐ Moderately severe disability; unable to walk without assistance and unable to attend to own bodily needs without assistance

☐ Severe disability; bedridden, incontinent and requiring constant nursing care and attention

**E. ACTIVITIES OF DAILY LIVING (ADL) AND INSTRUMENTAL ACTIVITIES OF DAILY LIVING (IADL)**

| E1 **Bathing** – either sponge bath, tub bath, shower or washing the body | 1 receives no assistance (gets in and out of tub alone if tub is usual means of bathing)---*skip to E2*  2 receives assistance in bathing only for part of the body (such as back or a leg)  3 receives assistance in bathing more than one part of the body (or doesn’t bathe) |
| --- | --- |
| E1.0 If receiving assistance, for how long? | _________ days |
| E2 **Dressing –** gets clothes from closets and drawers – including underwear, outer garments and fasteners (including suspenders, if worn) | 1 gets clothes and gets completely dressed without assistance--- *skip to E3*  2 gets clothes and gets dressed without assistance except for tying shoes  3 receives assistance in getting clothes or in getting dressed, or stays partly or completely undressed |
| E2.0 If receiving assistance, for how long? | _________ days |
| E3 **Toilet –** going to the toilet; cleaning oneself afterwards | 1 goes to the toilet, cleans self, and arranges clothes without assistance (may use object for support such as cane, walker, or wheelchair)--- *skip to E4*  2 can partly manage on his/her won, and receives assistance in going to the toilet or in cleaning self or in arranging clothes afterwards or in use of night bedpan or commode  3 bedridden and needs complete assistance in use of night bedpan or commode in bed. |
| E3.0 If receiving assistance, for how long? | _________ days |
| E4 **Indoor** **Transfer** | 1 gets in and out of bed as well as in and out of a chair without assistance (may use object for support such as cane or walker)--- *skip to E5*  2 gets in and out of bed or chair with assistance  3 bedridden |
| E4.0 If receiving assistance, for how long? | _________ days |
| E5 **Continence** | 1 has complete control of urination and bowel movement without assistance--- *skip to E6*  2 has occasional ‘accidents’  3 supervision helps keep urine or bowel control; catheter is used or elder is incontinent |
| E5.0 If has occasional ‘accidents’ or needs supervision, for how long? | _________ days |
| E6 **Eating** | 1 feeds self without assistance--- *skip to E6.1*  2 feeds self, with some help  3 receives assistance in feeding or is fed partly or completely intravenously |
| E6.0 If receiving assistance, for how long? | _________ days |
| ***If the respondent chooses all 1 for E1 to E6, skip to E7*** | |
| E6.1 Who is the primary caregiver when you need assistance in above bathing, dressing, toileting, indoor transferring, continence, and eating? | 1 spouse  2 son  3 daughter-in-law  4 daughter  5 son-in-law  6 unmarried son and daughter  7 grandchild(ren)  8 relative(s)  9 friends and neighbors  10 social services  11 housekeeper  12 nobody (*skip to E6.6*) |
| E6.2 What is your primary caregiver’s attitude when she/he takes care of you? | 1 willing to do  2 impatient/unwilling to do  3 need respite care  4 don’t know |
| E6.3 How much is the total direct cost paid for caregiving last week? | ______ Yuan (*if more than 100,000, please code 99998*) |
| E6.4 Who mainly pays the above cost? | 1 self 2 spouse  3 children & their spouses  4 grandchildren & their spouses  5 state/collective 6 others |
| E6.5 Do you think the help that you received for the above six tasks met your needs? | 1 fully met  2 so so  3 unmet |
| E6.6 How many persons among your children, grandchildren and their spouses helped you for the above six tasks last week? | _____ persons (*If nobody, fill 0 and skip to E7*) |
| E6.7 How many hours in total did your children, grandchildren and their spouses help you last week? | ________ hours |
| E7 Can you visit your neighbors by yourself? | 1 yes, independently  2 yes, but need some help  3 no, can’t |
| E8 Can you go shopping by yourself? | 1 yes, independently  2 yes, but need some help  3 no, can’t |
| E9 Can you cook a meal by yourself whenever necessary? | 1 yes, independently  2 yes, but need some help  3 no, can’t |
| E10 Can you wash clothing by yourself whenever necessary? | 1 yes, independently  2 yes, but need some help  3 no, can’t |
| E11 Can you walk continuously for 1 kilometer at a time by yourself? | 1 yes, independently  2 yes, but need some help  3 no, can’t |
| E12 Can you lift a weight of 5kg, such as a heavy bag of groceries? | 1 yes, independently  2 yes, but need some help  3 no, can’t |
| E13 Can you continuously crouch and stand up three times? | 1 yes, independently  2 yes, but need some help  3 no, can’t |
| E14 Can you take public transportation by yourself? | 1 yes, independently  2 yes, but need some help  3 no, can’t |

**M: EuroQOL Five Dimensions Questionnaire (EQ-5D)**

By placing a tick in one box in each group below, please indicate which statements best describe your own health state today.

M1. MOBILITY

I have no problems in walking about

I have slight problems in walking about

I have moderate problems in walking about

I have severe problems in walking about

I am unable to walk about

M2. SELF-CARE

I have no problems washing or dressing myself

I have slight problems washing or dressing myself

I have moderate problems washing or dressing myself

I have severe problems washing or dressing myself

I am unable to wash or dress myself

M3. USUAL ACTIVITIES (e.g. work, study, housework, family or leisure activities)

I have no problems doing my usual activities

I have slight problems doing my usual activities

I have moderate problems doing my usual activities

I have severe problems doing my usual activities

I am unable to do my usual activities

M4. PAIN / DISCOMFORT

I have no pain or discomfort

I have slight pain or discomfort

I have moderate pain or discomfort

I have severe pain or discomfort

I have extreme pain or discomfort

M5. ANXIETY / DEPRESSION

I am not anxious or depressed

I am slightly anxious or depressed

I am moderately anxious or depressed

I am severely anxious or depressed

I am extremely anxious or depressed

M6. Next, I would like to ask you to tell me how good or bad your health is today. (Show the patient the scale.) The top of the scale is a score of 100, which represents the best health you have in mind, and the bottom of the scale is a score of 0, which represents the worst health you have in mind. Now I would like you to tell me how many points you would like to rate your health today on this scale. (Please ask the patient to point out the position)

Record the value corresponding to the marked position _________

**N: IN THE PAST 2 WEEKS, HOW OFTEN HAVE YOU BEEN BOTHERED BY THE FOLLOWING PROBLEMS?**

|  | Almost every day 3 | More than half the time 2 | A few days 1 | Not at all 0 |
| --- | --- | --- | --- | --- |
| No interest or pleasure in doing things |  |  |  |  |
| Poor mood, depression or lack of hope in life |  |  |  |  |

If the sum of the scores from the previous two questions is greater than or equal to 3, then complete the following 7 questions. If the sum of the scores on the last two questions is less than 3, then skip.

|  | Almost every day 3 | More than half the time 2 | A few days 1 | Not at all 0 |
| --- | --- | --- | --- | --- |
| Difficulty falling asleep, or waking up easily, or sleeping too much |  |  |  |  |
| Feeling tired or lacking energy |  |  |  |  |
| Have a poor appetite or overeat |  |  |  |  |
| Feeling poorly about yourself, or being a failure, or letting yourself or your family down |  |  |  |  |
| Have difficulty concentrating on things, such as reading the newspaper or watching TV |  |  |  |  |
| Moving or speaking slowly enough for others to see; or conversely, becoming more irritable or fidgety than usual, walking around |  |  |  |  |
| Has thoughts that it would be better to die than to live, or to hurt yourself in some way |  |  |  |  |

**O: SECOND BLOOD PRESSURE MEASUREMENT**

Oa Please enter the value of the first blood pressure measurement for systolic pressure (high pressure): ________mmHg

Ob Please enter the value of the first blood pressure measurement for diastolic pressure (low pressure): ________mmHg

**P: DRUG USE - ASPIRIN**

P1. Are you taking anti-platelet (aspirin) medication?

Yes No(Jump to lipid-lowering drugs)

P2. Drug classes

Aspirin Bayaspirin Clopidogrel Dipyridamole

Cilostazol Ozagrel Other______

For this type of medicine:

P3a. When did you start taking this medicine?

Within 6 months

Within 6 months to 1 year

1 to 3 years

More than 3 years

P3b. How many months in the past year have you been taking the drug? ______ months

P4. How many days in the past two weeks have you been taken the drug? _______ days

P5. Did you take the medicine today? Yes No

P6. Did you sometimes forget to take your medication? Yes No

P7. Did you ever fail to take your medication? Yes No

P8. Did you sometimes stop taking your medication when you feel your symptoms are getting better?

Yes No

P9. Have you reduced or stopped taking your medication on your own without informing your doctor because you felt worse after taking it?

Yes No

P10. Why did you not take it as prescribed by your doctor?

☐ The medication is too expensive

☐ I forget to take it

☐ It doesn't work

☐ There are side effects

☐ I'm worried about side effects

☐ I don't feel I need to take it every day and can stop when my symptoms improve

☐ I think I'm taking too much medication in a day

☐ I'm on other medication

☐ I'm out of medication (lack of medication)

☐ I don't want to take it

☐ Other reasons (please specify) _____________

**Q: DRUG USE - LIPID-LOWERING DRUGS**

Q1. Are you taking lipid-lowering statins?

Yes No

Q2. Drug classes

Simvastatin Pravastatin Atorvastatin Other______

For this type of medicine:

Q3a. When did you start taking this medicine?

Within 6 months

Within 6 months to 1 year

1 to 3 years

More than 3 years

Q3b. How many months in the past year have you been taking the drug? ______ months

Q4. How many days in the past two weeks have you been taken the drug? _______ days

Q5. Did you take the medicine today? Yes No

Q6. Did you sometimes forget to take your medication? Yes No

Q7. Did you ever fail to take your medication? Yes No

Q8. Did you sometimes stop taking your medication when you feel your symptoms are getting better?

Yes No

Q9. Have you reduced or stopped taking your medication on your own without informing your doctor because you felt worse after taking it?

Yes No

Q10. Why did you not take it as prescribed by your doctor?

☐ The medication is too expensive

☐ I forget to take it

☐ It doesn't work

☐ There are side effects

☐ I'm worried about side effects

☐ I don't feel I need to take it every day and can stop when my symptoms improve

☐ I think I'm taking too much medication in a day

☐ I'm on other medication

☐ I'm out of medication (lack of medication)

☐ I don't want to take it

☐ Other reasons (please specify) _____________

**R: DRUG USE - ANTIHYPERTENSIVE DRUGS**

R1. Are you taking antihypertensive drugs?

Yes No

R2. Drug classes

Nifedipine Extended release nifedipine tablets Nitrendipine Amlodipine

Enalapril Captopril Hydrochlorothiazide Indapamide Compound reserpine tablets

Lacidipine Felodipine Sustained-release Tablets Levoamlodipine

Benazepril Fosinopril Lisinopril Losartan Telmisartan

Irbesartan Valsartan Atenolol Metoprolol Bisoprolol

Compound Dibazol hydrochlorothiazide capsules Propranolol hydrochloride tablets

Compound Hypotensive Tablets Others______

For this type of medicine:

R3a. When did you start taking this medicine?

Within 6 months

Within 6 months to 1 year

1 to 3 years

More than 3 years

R3b. How many months in the past year have you been taking the drug? ______ months

R4. How many days in the past two weeks have you been taken the drug? _______ days

R5. Did you take the medicine today? Yes No

R6. Did you sometimes forget to take your medication? Yes No

R7. Did you ever fail to take your medication? Yes No

R8. Did you sometimes stop taking your medication when you feel your symptoms are getting better?

Yes No

R9. Have you reduced or stopped taking your medication on your own without informing your doctor because you felt worse after taking it?

Yes No

R10. Why did you not take it as prescribed by your doctor?

☐ The medication is too expensive

☐ I forget to take it

☐ It doesn't work

☐ There are side effects

☐ I'm worried about side effects

☐ I don't feel I need to take it every day and can stop when my symptoms improve

☐ I think I'm taking too much medication in a day

☐ I'm on other medication

☐ I'm out of medication (lack of medication)

☐ My blood pressure is too low

☐ I don't want to take it

☐ Other reasons (please specify) _____________

**S: DRUG USE - DIABETES-RELATED DRUGS**

S1. Are you taking glucose-lowering drugs or insulin injections??

Yes No

S2. Drug classes

Insulin Sulfonylurea（Glipizide、Gliquidone、Glyburide、Gliclazide etc.）

Metformin α Glycosidase inhibitors (Acarbose, etc.) Glinides (Repaglinides, etc.)

Other______

For this type of medicine:

S3a. When did you start taking this medicine?

Within 6 months

Within 6 months to 1 year

1 to 3 years

More than 3 years

S3b. How many months in the past year have you been taking the drug? ______ months

S4. How many days in the past two weeks have you been taken the drug? _______ days

S5. Did you take the medicine today? Yes No

S6. Did you sometimes forget to take your medication? Yes No

S7. Did you ever fail to take your medication? Yes No

S8. Did you sometimes stop taking your medication when you feel your symptoms are getting better?

Yes No

S9. Have you reduced or stopped taking your medication on your own without informing your doctor because you felt worse after taking it?

Yes No

S10. Why did you not take it as prescribed by your doctor?

☐ The medication is too expensive

☐ I forget to take it

☐ It doesn't work

☐ There are side effects

☐ I'm worried about side effects

☐ I don't feel I need to take it every day and can stop when my symptoms improve

☐ I think I'm taking too much medication in a day

☐ I'm on other medication

☐ I'm out of medication (lack of medication)

☐ My blood sugar is too low

☐ I don't want to take it

☐ Other reasons (please specify) _____________

**T THIRD BLOOD PRESSURE MEASUREMENT**

If the difference between the two blood pressure values is 10mmHg, please measure the blood pressure for the third time and fill in the third blood pressure measurement value:

Ta Please enter the value of the second blood pressure measurement for systolic pressure (high pressure): ________mmHg

Tb Please enter the value of the second blood pressure measurement for diastolic pressure (low pressure): ________mmHg

**U Timed Up and Go**

On the word GO the patient will stand up, walk to the line on the floor (3 meters away from the chair) at his/her regular place, turn around and walk back to the chair and sit down.

Time (in seconds) needed to complete the task: ______

**V: Body Measurements**

V1. Height: ______

V2. Weight: ______

V3. Waist circumference: ______

V4. Neck circumference: ______

V5. Grip strength: ______

**W: INVESTIGATOR'S SUMMARY OF THIS INTERVIEW**

W1. Do you think that the patient clearly understood the questions in the interview

☐ Not understood at all☐ Not understood☐ Generally understood

☐ Understood ☐ Very understood

W2. Do you think the patient was resistant to the interview?

☐ Not resistant at all ☐ Not resistant ☐ Generally cooperative

☐ Relatively resistant ☐ Very resistant

W3. Do you think the patient answered the questions truthfully?

☐ Completely untrue☐ Relatively untrue ☐ Mostly true

☐ Relatively true ☐ Completely true

W4. How many of the above questions were answered by the patient themselves rather than with the help of family members?

☐All answered by the patient ☐25% answered by family ☐50% answered by family

☐75% answered by family ☐100% answered by family

☐ Other ________________________________________________________

W5. Please ask the interviewer to fill in their initials ______

## 5.3 Appendix B3. Interview outline for SINEMA long-term follow-up

### Interview guide for patients

**Introduction:** Thank the participant for their time and explain the purpose of the interview.

Clarify that their participation is voluntary and their responses will be kept confidential.

Obtain consent for recording the interview.

**Step 1:** Building Rapport: Begin with light conversation to make the participant feel comfortable. Share your interest in understanding their personal experience with the SINEMA intervention.

**Step 2:** Conducting the Interview

**Question 1: Stroke Experience and Health Management Post-Intervention**

- Since the SINEMA intervention ended, could you describe how you've been managing your health and any specific stroke-related care you've continued?

- Are there any particular habits or practices recommended during the intervention that you've maintained? Why do you think you've continued these?

**Question 2: Continued Practices from Intervention**

- Has the SINEMA intervention led to any long-lasting changes in your daily routine, especially regarding your stroke management?

- What advice or aspects of the intervention have you found most valuable and continue to use?

**Question 3: Support Systems**

- What kind of support do you feel is essential for you to continue managing your health effectively? This could be from healthcare providers, family, community, etc.

- Can you give examples of how your family or community has supported you in maintaining the health practices initiated during the SINEMA intervention?

**Question 4: Medication Adherence Post-Intervention**

- How do you manage your medication schedule now that the intervention has ended? Have you faced any challenges with this?

- If the daily voice messages or handouts were discontinued after a year, how did that affect your medication adherence?

**Question 5: Communication Preferences**

- After the intervention, what has been your preferred method of receiving health information and why?

- How effective do you find different forms of communication, like text messages, voice messages, or direct conversations with healthcare providers, in helping you manage your health?

**Question 6: Impact of COVID-19**

- How has the COVID-19 pandemic affected the way you manage your health, particularly in relation to stroke management?

- What adaptations have you made to your health management routine as a result of the pandemic?

**Question 7: Lifestyle Changes and Health Behaviors**

- Apart from medication adherence, what lifestyle changes have you implemented since the intervention? This can include diet, exercise, smoking cessation, etc.

- How sustainable have these lifestyle changes been, and what do you think has contributed to your adherence to these changes?

**Question 8: Additional Feedback and Suggestions**

- Reflecting on the SINEMA intervention, is there anything you would have liked to be done differently?

- Do you have any suggestions for additional support or resources that would help you or other stroke patients manage their health better?

**Step 3**: Conclusion

- Reiterate your thanks and ask if they have any final thoughts to share.

- Provide contact information for any further questions or support they might need.

- Assure them that the feedback they've provided will be invaluable in enhancing the SINEMA intervention for future participants.

Based on the provided outline and additional information, here is a revised and detailed interview guide tailored for village doctors (VDs), township, and county managers:

### Interview guide for VDs, township and county managers

**Introduction:** Thank you for your dedication and contribution to the SINEMA study. This interview aims to gather your insights on the SINEMA intervention model and the associated mobile application you have used. Your feedback is critical to refining our approach to better serve stroke patients in rural China.

**Consent:**

- Explain the interview process, confidentiality measures, and how the data will be used.

- Confirm that the participant consents to the interview and its recording.

**Step 1**: Rapport Building

- Begin with an informal conversation to establish a comfortable atmosphere.

- Show genuine interest in their professional experiences and roles in the SINEMA intervention.

**Step 2**: Detailed Interview Questions

**Question 1: Stroke Management Challenges**

- Reflecting on your experience, what are the primary challenges you face in stroke prevention and management within primary healthcare settings?

- How well do you think the national basic public health services cater to the needs of secondary stroke prevention?

**Question 2: Comparing SINEMA App with National Health Service System**

- In contrast to the traditional national public health service system, what benefits or challenges has the SINEMA App presented?

**Question 3: Training Efficacy and Content**

- Can you describe the impact of the training on your medical knowledge and practices?

- Which parts of the training were most beneficial, and which could be improved or omitted?

- Are there crucial topics that the training did not cover that you feel should be included?

**Question 4: Follow-up Visits and Patient Management**

- How did you organize the follow-up visits with the patients? Were these conducted at clinics or at the patients' homes?

- Could you describe the integration of the follow-up visits with your regular duties?

- What is your perspective on the workload? Is the frequency and duration of the intervention feasible for you to manage?

**Question 5: Medication Adherence Monitoring**

- How do you assess and ensure medication adherence among your patients?

- What methods do you use to measure their blood pressure, and how often?

**Question 6: SINEMA App Usability and Impact**

- Share your experience with learning and using the SINEMA App.

- Has the App facilitated better patient management, or has it introduced new complexities?

- Could you provide any suggestions for improving the App's design and functionality?

**Question 7: Performance, Compensation, and Support Systems**

- What key performance indicators do you believe would effectively measure the success of interventions like SINEMA?

- Discuss the compensation structure and whether it aligns with your expectations and workload.

- Evaluate the support provided by the township hospital managers. Are there ways this support could be enhanced?

**Question 8: Communication Tools and Peer Support**

- How effective is the WeChat group in fostering communication and support among healthcare providers involved in the SINEMA intervention?

**Question 9: Long-term Adaptations and Sustainability**

- Since the official SINEMA intervention period has concluded, what strategies have you employed to sustain its practices and principles in your routine healthcare services?

- Can you describe any modifications you have made to the intervention's methods to better fit the local context or to address any barriers that have arisen over time?

- In terms of the technologies and methods introduced by SINEMA, which do you believe have the potential for long-term integration into your health management practices?

- How do you foresee the continuation of these health practices without the direct support of the SINEMA program, particularly in terms of resources and training?

- What additional tools, resources, or changes in policy would you consider essential to ensure the ongoing success and sustainability of the practices introduced by the SINEMA intervention?

**Question 10: Additional Feedback**

- Is there any other feedback or suggestions you would like to share that we haven’t covered?

**Step 3:** Conclusion

- Thank the interviewee for sharing their valuable insights and contributing their time.

- Hand over a small token of appreciation to show gratitude for their participation.

- Ensure the interviewee knows how to contact you for any follow-up questions or further information.

- Close the conversation by reiterating the importance of their feedback to the improvement of the SINEMA project.

# **REFERENCES:**

1. Zhou M, Wang H, Zhu J, et al. Cause-specific mortality for 240 causes in China during 1990-2013: a systematic subnational analysis for the Global Burden of Disease Study 2013. *Lancet*. Jan 16 2016;387(10015):251-72. doi:10.1016/s0140-6736(15)00551-6

2. Liu M, Wu B, Wang WZ, Lee LM, Zhang SH, Kong LZ. Stroke in China: epidemiology, prevention, and management strategies. *Lancet Neurol*. May 2007;6(5):456-64. doi:10.1016/s1474-4422(07)70004-2

3. Global Burden of Disease Collaborative Network. Global Burden of Disease Study 2016 (GBD 2016) Cause-Specific Mortality 1980-2016. Seattle, United States of America: Institute for Health Metrics and Evaluation (IHME), 2017.

4. Wang W, Jiang B, Sun H, et al. Prevalence, Incidence, and Mortality of Stroke in China: Results from a Nationwide Population-Based Survey of 480 687 Adults. *Circulation*. Feb 21 2017;135(8):759-771. doi:10.1161/circulationaha.116.025250

5. Xu G, Liu X, Wu W, Zhang R, Yin Q. Recurrence after ischemic stroke in chinese patients: impact of uncontrolled modifiable risk factors. *Cerebrovasc Dis*. 2007;23(2-3):117-20. doi:10.1159/000097047

6. Hankey GJ, Warlow CP. Treatment and secondary prevention of stroke: evidence, costs, and effects on individuals and populations. *Lancet*. Oct 23 1999;354(9188):1457-63. doi:10.1016/s0140-6736(99)04407-4

7. Rashid P, Leonardi-Bee J, Bath P. Blood pressure reduction and secondary prevention of stroke and other vascular events: a systematic review. *Stroke*. Nov 2003;34(11):2741-8. doi:10.1161/01.Str.0000092488.40085.15

8. Warlow C. Secondary prevention of stroke. *Lancet*. Mar 21 1992;339(8795):724-7. doi:10.1016/0140-6736(92)90608-6

9. Chinese guideline for cerebrovascular disease tretment in primary healthcare settings (Peking Union Medical College Press) (2016).

10. World Health O. Prevention of recurrent heart attacks and strokes in low and middle income populations : evidence-based recommendations for policy-makers and health professionals. Geneva: World Health Organization; 2004.

11. Wei JW, Wang JG, Huang Y, et al. Secondary prevention of ischemic stroke in urban China. *Stroke*. May 2010;41(5):967-74. doi:10.1161/strokeaha.109.571463

12. Fuster V. Global burden of cardiovascular disease: time to implement feasible strategies and to monitor results. *J Am Coll Cardiol*. Aug 5 2014;64(5):520-2. doi:10.1016/j.jacc.2014.06.1151

13. Zhao M, Li X, Zheng L, et al. Optimal Blood Pressure in Patients after Stroke in Rural Areas of China. *J Stroke Cerebrovasc Dis*. Feb 2016;25(2):270-80. doi:10.1016/j.jstrokecerebrovasdis.2015.09.028

14. Yip W, Hsiao W. Harnessing the privatisation of China's fragmented health-care delivery. *Lancet*. Aug 30 2014;384(9945):805-18. doi:10.1016/s0140-6736(14)61120-x

15. Shi Y, Yi H, Zhou H, et al. The quality of primary care and correlates among grassroots providers in rural China: a cross-sectional standardised patient study. *The Lancet*. 2017;390:S16.

16. Kruk ME, Nigenda G, Knaul FM. Redesigning primary care to tackle the global epidemic of noncommunicable disease. *Am J Public Health*. Mar 2015;105(3):431-7. doi:10.2105/ajph.2014.302392

17. Liu X, Sun X, Zhao Y, Meng Q. Financial protection of rural health insurance for patients with hypertension and diabetes: repeated cross-sectional surveys in rural China. *BMC Health Serv Res*. Sep 8 2016;16(1):481. doi:10.1186/s12913-016-1735-5

18. Yan R, Li W, Yin L, Wang Y, Bo J. Cardiovascular Diseases and Risk-Factor Burden in Urban and Rural Communities in High-, Middle-, and Low-Income Regions of China: A Large Community-Based Epidemiological Study. *J Am Heart Assoc*. Feb 6 2017;6(2)doi:10.1161/jaha.116.004445

19. Jiang Y, Yang X, Li Z, et al. Persistence of secondary prevention medication and related factors for acute ischemic stroke and transient ischemic attack in China. *Neurol Res*. Jun 2017;39(6):492-497. doi:10.1080/01616412.2017.1312792

20. Labrique AB, Vasudevan L, Kochi E, Fabricant R, Mehl G. mHealth innovations as health system strengthening tools: 12 common applications and a visual framework. *Glob Health Sci Pract*. Aug 2013;1(2):160-71. doi:10.9745/ghsp-d-13-00031

21. Hamine S, Gerth-Guyette E, Faulx D, Green BB, Ginsburg AS. Impact of mHealth chronic disease management on treatment adherence and patient outcomes: a systematic review. *J Med Internet Res*. Feb 24 2015;17(2):e52. doi:10.2196/jmir.3951

22. Beratarrechea A, Lee AG, Willner JM, Jahangir E, Ciapponi A, Rubinstein A. The impact of mobile health interventions on chronic disease outcomes in developing countries: a systematic review. *Telemed J E Health*. Jan 2014;20(1):75-82. doi:10.1089/tmj.2012.0328

23. Peiris D, Praveen D, Johnson C, Mogulluru K. Use of mHealth systems and tools for non-communicable diseases in low- and middle-income countries: a systematic review. *J Cardiovasc Transl Res*. Nov 2014;7(8):677-91. doi:10.1007/s12265-014-9581-5

24. Beratarrechea A, Moyano D, Irazola V, Rubinstein A. mHealth Interventions to Counter Noncommunicable Diseases in Developing Countries: Still an Uncertain Promise. *Cardiol Clin*. Feb 2017;35(1):13-30. doi:10.1016/j.ccl.2016.08.009

25. Gandhi S, Chen S, Hong L, et al. Effect of Mobile Health Interventions on the Secondary Prevention of Cardiovascular Disease: Systematic Review and Meta-analysis. *Can J Cardiol*. Feb 2017;33(2):219-231. doi:10.1016/j.cjca.2016.08.017

26. Tian M, Zhang J, Luo R, et al. mHealth Interventions for Health System Strengthening in China: A Systematic Review. *JMIR Mhealth Uhealth*. Mar 16 2017;5(3):e32. doi:10.2196/mhealth.6889

27. Tian M, Ajay VS, Dunzhu D, et al. A Cluster-Randomized, Controlled Trial of a Simplified Multifaceted Management Program for Individuals at High Cardiovascular Risk (SimCard Trial) in Rural Tibet, China, and Haryana, India. *Circulation*. Sep 1 2015;132(9):815-24. doi:10.1161/circulationaha.115.015373

28. Li N, Yan LL, Niu W, et al. The Effects of a Community-Based Sodium Reduction Program in Rural China - A Cluster-Randomized Trial. *PLoS One*. 2016;11(12):e0166620. doi:10.1371/journal.pone.0166620

29. Al-Fedaghi SA, Thalheim B. Databases of personal identifiable information. IEEE; 2008:617-624.

30. Fielding RT, Taylor RN. Principled design of the modern web architecture. *ACM Transactions on Internet Technology (TOIT)*. 2002;2(2):115-150.

31. Mosa AS, Yoo I, Sheets L. A systematic review of healthcare applications for smartphones. *BMC Med Inform Decis Mak*. Jul 10 2012;12:67. doi:10.1186/1472-6947-12-67

32. Sposaro F, Tyson G. iFall: an Android application for fall monitoring and response. IEEE; 2009:6119-6122.

33. Banerjee P. About Face 2.0: the essentials of interaction design: Alan Cooper and Robert Reimann published by John Wiley & Sons, 2003, 576 pp, ISBN 0764526413. SAGE Publications Sage UK: London, England; 2004.

34. Champion VL, Skinner CS. The health belief model. *Health behavior and health education: Theory, research, and practice*. 2008;4:45-65.

35. Diez-Canseco F, Zavala-Loayza JA, Beratarrechea A, et al. Design and multi-country validation of text messages for an mHealth intervention for primary prevention of progression to hypertension in Latin America. *JMIR mHealth and uHealth*. 2015;3(1):e3874.

36. China Statistic Press. Hebei Economic Yearbook 2016. 2016. URL: <http://www.hetj.gov.cn/res/nj2016/indexch.htm>

37. Xu G, Ma M, Liu X, Hankey GJ. Is there a stroke belt in China and why? *Stroke*. 2013;44(7):1775-1783.

38. *Health behavior and health education: Theory, research, and practice, 4th ed*. Health behavior and health education: Theory, research, and practice, 4th ed. Jossey-Bass; 2008:xxxiii, 552-xxxiii, 552.

39. Yan LL, Fang W, Delong E, et al. Population impact of a high cardiovascular risk management program delivered by village doctors in rural China: design and rationale of a large, cluster-randomized controlled trial. *BMC Public Health*. 2014;14:1-8.

40. Zhou B, Zhang J, Zhao Y, et al. Caregiver-delivered stroke rehabilitation in rural China: the RECOVER Randomized Controlled Trial. *Stroke*. 2019;50(7):1825-1830.

41. Hebei People's Government. Hebei Economic Yearbook 2016.China Statistic Press. 2016.

42. The World Bank. Mobile Cellular Subscriptions (per 100 people). 2016.

43. Yang X, Guo H, Zhao Y. Investigation and analysis of information infrastructure status and utilizaiton and farmers information behavior in rural areas of Hebei province. J Mod Inf 2013;33(5): 38-4

44. Feldstein CA. Lowering blood pressure to prevent stroke recurrence: a systematic review of long-term randomized trials. *J Am Soc Hypertens*. Jul 2014;8(7):503-13. doi:10.1016/j.jash.2014.05.002

45. Meurer WJ, Lewis RJ. Cluster randomized trials: evaluating treatments applied to groups. *Jama*. May 26 2015;313(20):2068-9. doi:10.1001/jama.2015.5199

46. Lauer MS, Mensah GA. Power of the Cluster. *Circulation*. Sep 1 2015;132(9):794-5. doi:10.1161/circulationaha.115.018142

47. Caille A, Kerry S, Tavernier E, Leyrat C, Eldridge S, Giraudeau B. Timeline cluster: a graphical tool to identify risk of bias in cluster randomised trials. *Bmj*. Aug 16 2016;354:i4291. doi:10.1136/bmj.i4291

48. Li X, Lu J, Hu S, et al. The primary health-care system in China. *Lancet*. Dec 9 2017;390(10112):2584-2594. doi:10.1016/s0140-6736(17)33109-4

49. Zhang J, Joshi R, Sun J, et al. A feasibility study on using smartphones to conduct short-version verbal autopsies in rural China. *Popul Health Metr*. 2016;14:31. doi:10.1186/s12963-016-0100-6

50. Mathias S, Nayak US, Isaacs B. Balance in elderly patients: the "get-up and go" test. *Arch Phys Med Rehabil*. Jun 1986;67(6):387-9.

51. Podsiadlo D, Richardson S. The timed "Up & Go": a test of basic functional mobility for frail elderly persons. *J Am Geriatr Soc*. Feb 1991;39(2):142-8. doi:10.1111/j.1532-5415.1991.tb01616.x

52. Morisky DE, Green LW, Levine DM. Concurrent and predictive validity of a self-reported measure of medication adherence. *Med Care*. Jan 1986;24(1):67-74. doi:10.1097/00005650-198601000-00007

53. Macfarlane DJ, Lee CC, Ho EY, Chan KL, Chan DT. Reliability and validity of the Chinese version of IPAQ (short, last 7 days). *J Sci Med Sport*. Feb 2007;10(1):45-51. doi:10.1016/j.jsams.2006.05.003

54. Herdman M, Gudex C, Lloyd A, et al. Development and preliminary testing of the new five-level version of EQ-5D (EQ-5D-5L). *Qual Life Res*. Dec 2011;20(10):1727-36. doi:10.1007/s11136-011-9903-x

55. Mathias S, Nayak U, Isaacs B. Balance in elderly patients: the" get-up and go" test. *Archives of physical medicine and rehabilitation*. 1986;67(6):387-389.

56. Podsiadlo D, Richardson S. The Timed “Up & Go”: A Test of Basic Functional Mobility for Frail Elderly Persons. *Journal of the American Geriatrics Society*. 1991;39(2):142-148. doi:10.1111/j.1532-5415.1991.tb01616.x

57. Morisky DE, Green LW, Levine DM. Concurrent and predictive validity of a self-reported measure of medication adherence. *Medical care*. 1986;24(1):67-74.

58. Macfarlane DJ, Lee CC, Ho EY, Chan K, Chan DT. Reliability and validity of the Chinese version of IPAQ (short, last 7 days). *Journal of Science and Medicine in Sport*. 2007;10(1):45-51.

59. Li P, Redden DT. Comparing denominator degrees of freedom approximations for the generalized linear mixed model in analyzing binary outcome in small sample cluster-randomized trials. *BMC Med Res Methodol*. Apr 23 2015;15:38. doi:10.1186/s12874-015-0026-x

60. Laird NM, Ware JH. Random-effects models for longitudinal data. *Biometrics*. Dec 1982;38(4):963-74.

61. Turner EL, Prague M, Gallis JA, Li F, Murray DM. Review of Recent Methodological Developments in Group-Randomized Trials: Part 2-Analysis. *Am J Public Health*. Jul 2017;107(7):1078-1086. doi:10.2105/ajph.2017.303707

62. Zou GY, Donner A. Extension of the modified Poisson regression model to prospective studies with correlated binary data. *Stat Methods Med Res*. Dec 2013;22(6):661-70. doi:10.1177/0962280211427759

63. Groenwold RH, Donders AR, Roes KC, Harrell FE, Jr., Moons KG. Dealing with missing outcome data in randomized trials and observational studies. *Am J Epidemiol*. Feb 1 2012;175(3):210-7. doi:10.1093/aje/kwr302

64. Groenwold RH, Moons KG, Vandenbroucke JP. Randomized trials with missing outcome data: how to analyze and what to report. *Cmaj*. Oct 21 2014;186(15):1153-7. doi:10.1503/cmaj.131353

65. Fiero MH, Hsu CH, Bell ML. A pattern-mixture model approach for handling missing continuous outcome data in longitudinal cluster randomized trials. *Stat Med*. Nov 20 2017;36(26):4094-4105. doi:10.1002/sim.7418

66. Campbell MK, Elbourne DR, Altman DG. CONSORT statement: extension to cluster randomised trials. *Bmj*. Mar 20 2004;328(7441):702-8. doi:10.1136/bmj.328.7441.702

67. Gong E, Gu W, Sun C, et al. System-integrated technology-enabled model of care to improve the health of stroke patients in rural China: protocol for SINEMA-a cluster-randomized controlled trial. *Am Heart J*. Jan 2019;207:27-39. doi:10.1016/j.ahj.2018.08.015

68. Hall KS, Hendrie HC, Brittain HM, Norton JA. The development of a dementia screening interview in two distinct languages. *International journal of methods in psychiatric research*. 1993;

69. Chan TS, Lam LC, Chiu HF, Prince M. Validity and applicability of the Chinese version of community screening instrument for dementia. *Dementia and geriatric cognitive disorders*. 2002;15(1):10-18.

70. Duncan PW, Jorgensen HS, Wade DT. Outcome measures in acute stroke trials: a systematic review and some recommendations to improve practice. *Stroke*. 2000;31(6):1429-1438.

71. Gong E, Yan LL, McCormack K, Gallis JA, Bettger JP, Turner EL. System-integrated technology-enabled model of care (SINEMA) to improve the health of stroke patients in rural China: Statistical analysis plan for a cluster-randomized controlled trial. *Int J Stroke*. Feb 2020;15(2):226-230. doi:10.1177/1747493019869707

72. Gallis JA, Turner EL. Relative Measures of Association for Binary Outcomes: Challenges and Recommendations for the Global Health Researcher. *Ann Glob Health*. Nov 20 2019;85(1):137. doi:10.5334/aogh.2581
