# Supplementary material for: Long-term mortality outcome of a primary care-based mobile health intervention for stroke management: Six-year follow-up of a cluster-randomized controlled trial
Source: PLoS Med. 2025 Mar 27;22(3):e1004564. doi: 10.1371/journal.pmed.1004564 (PMC11949329; doi:10.1371/journal.pmed.1004564)
Supplement: S1 File — Fig A. Timeline for trial phase and post-trial follow-up phase in SINEMA. Table A. Full list of mortality outcomes with ICD-10 codes used in the present study. Appendix A. Definitions, measurement for intermediate outcomes in S1 File. Appendix B. Statistical analysis plan for intermediate outcomes in S1 File. Table B. Baseline characteristics of participants who were followed and deceased at 70-month follow-up (n = 1,255). Table C. Baseline characteristics by status of follow-up among participants who survived at 70 months (n = 1,023). Fig B. Flow diagram for confirming mortality information from three sources. Table D. Baseline characteristics of participants of the SINEMA trial who died during 70-months follow-up post-baseline (n = 276). Table E. Baseline characteristics by intervention arms of participants who survived at 70-months post-baseline (n = 1,023). Table F. Baseline characteristics of participants in the SINEMA trial by vital status at 70-months post-baseline (n = 1,299). Table G. The adjusted hazard ratios (95% confidence intervals) of intervention on mortality in patients experiencing stroke at 70-month follow-up using sub-distribution model. Fig C. Long-term outcomes on cardiovascular cause-specific mortality in each pre-specified and post-hoc subgroup, minimally adjusted model. Fig D. Long-term outcomes on stroke cause-specific mortality in each pre-specified and post-hoc subgroup, minimally adjusted model. Table H. Effects (risk difference) of the SINEMA intervention on intermediate outcomes from baseline to 70 months among living patients (n = 979). Table I. Participants characteristics and intermediate outcomes at baseline and at the long-term follow-up (n = 979). (DOCX) [file pmed.1004564.s002.docx]

**Long-term mortality outcome of a primary care-based mobile health intervention for stroke management: six year follow-up of a cluster-randomized controlled trial**

**S1 Supporting information**

**Table of Contents**

**Figure A.** Timeline for trial phase and post-trial follow-up phase in SINEMA1

**Table A.** Full list of mortality outcomes with ICD-10 codes used in the present study2

**Appendix A.** Definitions, measurement for intermediate outcomes5

**Appendix B.** Statistical analysis plan for intermediate outcomes6

**Table B.** Baseline characteristics of participants who were followed and deceased at 70-month follow-up (n=1255)7

**Table C.** Baseline characteristics by status of follow-up among participants who survived at 70 months (n=1023)10

**Figure B.** Flow diagram for confirming mortality information from three sources 13

**Table D.** Baseline characteristics of participants of the SINEMA trial who died during 70-months follow-up post-baseline (n=276)14

**Table E.** Baseline characteristics by intervention arms of participants who survived at 70-months post-baseline (n=1023)17

**Table F.** Baseline characteristics of participants in the SINEMA trial by vital status at 70-months post-baseline (n=1299)20

**Table G.** The adjusted hazard ratios (95% confidence intervals) of intervention on mortality in patients experiencing stroke at 70-month follow-up using sub-distribution model 20

**Figure C.** Long-term outcomes on cardiovascular cause-specific mortality in each pre-specified and post-hoc subgroup, minimally adjusted model21

**Figure D.** Long-term outcomes on stroke cause-specific mortality in each pre-specified and post-hoc subgroup, minimally adjusted model 22

**Table H.** Effects (risk difference) of the SINEMA intervention on intermediate outcomes from baseline to 70 months among living patients (n=979) 25

**Table I.** Participants characteristics and intermediate outcomes at baseline and at the long-term follow-up (n=979)25

**
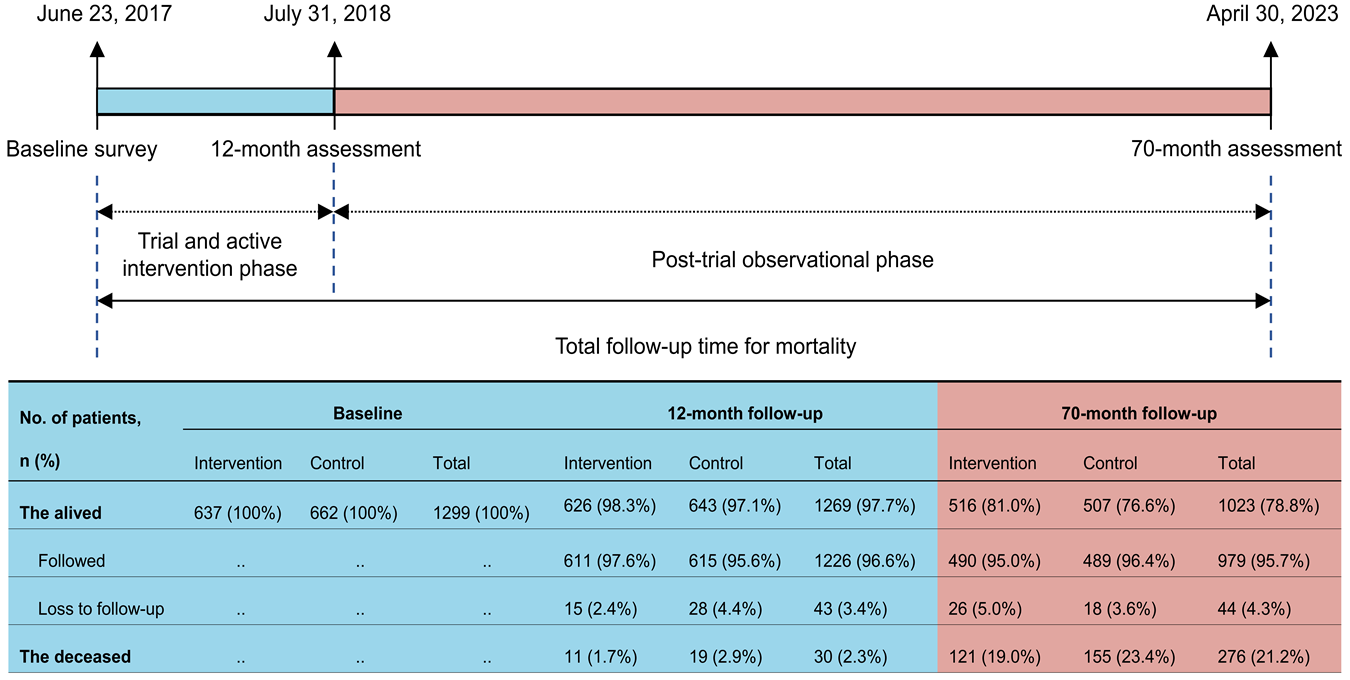
**

**Figure A.** **Timeline for trial phase and post-trial follow-up phase of the SINEMA study**

| **Table A. Full list of causes of death with ICD-10 codes used in the study** | |
| --- | --- |
| **Causes of death** | **ICD-10 code** |
| **Cardiovascular diseases** |  |
| **Non-stroke cardiovascular diseases** |  |
| Rheumatic heart disease, unspecified | I09.9 |
| Hypertensive heart disease | I11 |
| Hypertensive heart disease with (congestive) heart failure | I11.0 |
| Hypertensive renal disease with renal failure | I12.0 |
| Hypertensive heart and renal disease | I13 |
| Hypertensive heart and renal disease with both (congestive)  heart failure and renal failure | I13.2 |
| Secondary hypertension | I15 |
| Acute myocardial infarction | I21 |
| Acute myocardial infarction, unspecified | I21.9 |
| Other acute ischaemic heart diseases | I24 |
| Chronic ischaemic heart disease | I25 |
| Atherosclerotic heart disease | I25.1 |
| Pulmonary embolism with mention of acute cor pulmonale | I26.0 |
| Pulmonary heart disease, unspecified | I27.9 |
| Heart failure | I50 |
| Heart failure, unspecified | I50.9 |
| Atherosclerosis | I70 |
| Arteritis, unspecified | I77.6 |
| **Stroke** |  |
| Intracerebral haemorrhage | I61 |
| **Table A. Continued** | |
| **Causes of death** | **ICD-10 code** |
| Intracerebral haemorrhage, unspecified | I61.9 |
| Cerebral infarction | I63 |
| Cerebral infarction due to thrombosis of cerebral arteries | I63.3 |
| Cerebral infarction due to embolism of cerebral arteries | I63.4 |
| Stroke, not specified as haemorrhage or infarction | I64 |
| Cerebral atherosclerosis | I67.2 |
| Hypertensive encephalopathy | I67.4 |
| Sequelae of intracerebral haemorrhage | I69.1 |
| Sequelae of cerebral infarction | I69.3 |
| **Other diseases** |  |
| Tuberculosis of lung, without mention of bacteriological or  histological confirmation | A16.2 |
| Pulmonary mycobacterial infection | A31.0 |
| Oesophagus, unspecified | C15.9 |
| Malignant neoplasm of stomach | C16 |
| Stomach, unspecified | C16.9 |
| Malignant neoplasm of rectum | C20 |
| Liver cell carcinoma | C22.0 |
| Malignant neoplasm of bronchus and lung | C34 |
| Bronchus or lung, unspecified | C34.9 |
| Malignant neoplasm of prostate | C61 |
| Malignant neoplasm of bladder | C67 |
| Malignant neoplasm of brain | C71 |
| **Table A. Continued** |  |
| **Causes of death** | **ICD-10 code** |
| Malignant neoplasm of brain, unspecified | C71.9 |
| Malignant neoplasm, primary site unspecified | C80.9 |
| Type 1 diabetes mellitus | E10 |
| Type 2 diabetes mellitus | E11 |
| Epilepsy, unspecified | G40.9 |
| Viral pneumonia, unspecified | J12.9 |
| Pneumonia in viral diseases classified elsewhere | J17.1 |
| Pneumonia, unspecified | J18.9 |
| Chronic obstructive pulmonary disease, unspecified | J44.9 |
| Hepatic sclerosis | K74.1 |
| Other and unspecified cirrhosis of liver | K74.6 |
| Fracture of bone in neoplastic disease | M90.7 |
| Unspecified kidney failure | N19 |
| Cachexia | R64 |
| Other specified general symptoms and signs | R68.8 |
| Other sudden death, cause unknown | R96 |
| Pedestrian injured in collision with pedal cycle | V01.1 |
| Pedestrian injured in traffic accident involving other and  unspecified motor vehicles | V09.2 |
| Intentional self-harm by jumping from a high place | X80 |
| ICD-10=International Statistical Classification of Diseases and Related Health Problems, Tenth Revision | |

**Appendix A. Definition and measurement**

Blood pressure is measured on the right upper arm (or left arm if the right arm is disabled) with participant seated after 5 minutes of rest using an electronic blood pressure monitor (Omron HEM-7052). Two measurements are taken, and the mean value is calculated. If the differences between the 2 systolic blood pressure is larger than 10 mm Hg, a third measurement is conducted, and the mean value of the only or the last 2 reading is calculated. Physical measurements included height, weight, waist circumference, and mobility. Mobility is measured using the Timed Up and Go test, a simple and quick functional mobility test that requires the participants to stand up, walk 3 meters, turn, walk back, and sit down [1,2]. Medication adherence is measured using the 4-item Morisky Green Levine Scale with scores ranging from 0 to 4 with a higher score indicating lower medication adherence [3]. Participants will respond to the 4 questions with yes or no for each type of medicines (anti-hypertension, aspirin and statin) if they were prescribed by the physicians before [3]. Physical activity level is measured using the short version of the International Physical Activity Questionnaire [4]. Participants were asked to recall the frequency and duration of rigorous activities, moderate activities and walking they performed in the past 7 days.

**References**

1. Mathias S, Nayak U, Isaacs B. Balance in elderly patients: the “get-up and go” test. Arch Phys Med Rehabil 1986; 67(6): 387-9.

2. Podsiadlo D, Richardson S. The timed “Up & Go”: a test of basic functional mobility for frail elderly persons. J Am Geriatr Soc 1991; 39(2): 142-8.

3. Morisky DE, Green LW, Levine DM. Concurrent and predictive validity of a self-reported measure of medication adherence. Med Care 1986; 24(1): 67-74.

4. Macfarlane DJ, Lee CC, Ho EY, et al. Reliability and validity of the Chinese version of IPAQ (short, last 7 days). J Sci Med Sport 2007; 10(1): 45-51.

**Appendix B. *St*atistical analysis plan for intermediate outcomes**

For continuous variables, we applied mixed-effects linear regression models to estimate between-arm differences in mean outcomes at 5.5 years, with a random intercept for the cluster (village) and a fixed effect for townships, baseline outcome, age, sex, and interview month (minimally adjusted model). Restricted maximum likelihood was used together with the between-within method to calculate degrees of freedom [1]. We removed outliers from all main analyses based on an a priori decision to remove those that were more than two interquartile ranges above the third quartile or below the first quartile [2]. When the mixed effects model did not converge, we employed the generalized estimating equations (GEE) method with Gaussian model and an identity link. For binary outcome variables, GEE approach was used to obtain population-averaged intervention effects using a Poisson model with both log and identity link and robust standard errors in order to obtain probability ratios and differences, respectively (typically referred to as risk ratios and risk differences, respectively) [3]. All GEE models used an independence working correlation [3]. If GEE models did not converge, we used a marginal standardization approach by fitting a binomial model with a logit link to obtain risk differences and risk ratios [4].

**References**

1. Li P, Redden DT. Comparing denominator degrees of freedom approximations for the generalized linear mixed model in analyzing binary outcome in small sample cluster-randomized trials. BMC Medical Research Methodology 2015; 15(1): 38.

2. Gong E, Yan LL, McCormack K, Gallis JA, Bettger JP, Turner EL. System-integrated technology-enabled model of care (SINEMA) to improve the health of stroke patients in rural China: Statistical analysis plan for a cluster-randomized controlled trial. International Journal of Stroke 2019; 15(2): 226-30.

3. Zou GY, Donner A. Extension of the modified Poisson regression model to prospective studies with correlated binary data. Statistical Methods in Medical Research 2011; 22(6): 661-70.

4. Gallis JA, Turner EL. Relative Measures of Association for Binary Outcomes: Challenges and Recommendations for the Global Health Researcher. Ann Glob Health 2019; 85(1): 137.

| **Table B. Baseline characteristics of participants who were followed and deceased at 70-month follow-up (n=1255)** | | | | | | | | |
| --- | --- | --- | --- | --- | --- | --- | --- | --- |
| **Characteristics** | **Followed** |  | | **Deceased** |  | **Total** |  | **P value** |
|  | 979 (78.0%) | | 276 (22.0%) | | 1255 (100%) | | |  |
| Intervention allocation |  | |  | |  | | | 0.08 |
| Control | 489 (49.9%) | | 155 (56.2%) | | 644 (51.3%) | | |  |
| Intervention | 490 (50.1%) | | 121 (43.8%) | | 611 (48.7%) | | |  |
| Age, years | 64.9 (8.2) | | 69.0 (7.1) | | 65.8 (8.2) | | | <0.001 |
| Sex |  | |  | |  | | | <0.001 |
| Female | 451 (46.1%) | | 84 (30.4%) | | 535 (42.6%) | | |  |
| Male | 528 (53.9%) | | 192 (69.6%) | | 720 (57.4%) | | |  |
| Education |  | |  | |  | | | 0.59 |
| No schooling | 414 (42.3%) | | 109 (39.5%) | | 523 (41.7%) | | |  |
| Some or primary school only | 292 (29.8%) | | 82 (29.7%) | | 374 (29.8%) | | |  |
| Above primary school | 273 (27.9%) | | 85 (30.8%) | | 358 (28.5%) | | |  |
| Marital status |  | |  | |  | | | 0.30 |
| Married | 816 (83.4%) | | 222 (80.4%) | | 1038 (82.7%) | | |  |
| Widowed, diveorced, or not married | 163 (16.6%) | | 54 (19.6%) | | 217 (17.3%) | | |  |
| Annual income, CNY |  | |  | |  | | | 0.70 |
| ≤ 5000 | 556 (56.8%) | | 161 (58.3%) | | 717 (57.1%) | | |  |
| > 5000 | 423 (43.2%) | | 115 (41.7%) | | 538 (42.9%) | | |  |
| Phone ownership |  | |  | |  | | | <0.001 |
| No personal phone | 211 (21.6%) | | 104 (37.7%) | | 315 (25.1%) | | |  |
| Basic phone | 690 (70.4%) | | 160 (58.0% ) | | 850 (67.7%) | | |  |
| Smart phone | 78 (8.0%) | | 12 (4.3%) | | 90 (7.2%) | | |  |
| Smoking status |  | |  | |  | | | 0.002 |
| Current smoker | 154 (15.7%) | | 54 (19.6%) | | 208 (16.6%) | | |  |
| Former smoker | 182 (18.6%) | | 72 (26.1%) | | 254 (20.2%) | | |  |
| Never smoker | 643 (65.7%) | | 150 (54.3%) | | 793 (63.2%) | | |  |
| Health enhancing physical activity |  | |  | |  | | | <0.001 |
| Not achieving | 801 (81.8%) | | 253 (91.7%) | | 1054 (84.0%) | | |  |
| Achieving | 178 (18.2%) | | 23 (8.3%) | | 201 (16.0%) | | |  |
| Stroke type |  | |  | |  | | | 0.87 |
| **Table B. Continued** | | | | | | | | |
| **Characteristics** | **Followed** |  | | **Deceased** |  | **Total** |  | **P value** |
|  | 979 (78.0%) | | 276 (22.0%) | | 1255 (100%) | | |  |
| Ischemic | 844 (86.2%) | | 241 (87.3%) | | 1085 (86.5%) | | |  |
| Hemorrghage | 132 (13.5%) | | 34 (12.3%) | | 166 (13.2%) | | |  |
| Not specified | 3 (0.3%) | | 1 (0.4%) | | 4 (0.3%) | | |  |
| Stroke duration, years |  | |  | |  | | |  |
| Since the first event | 5.0 (2.1-9.7) | | 5.9 (3.0-11.1) | | 5.2 (2.3-9.8) | | | <0.001 |
| Since the last event | 3.3 (1.1-6.7) | | 3.2 (1.0-7.3) | | 3.3 (1.1-6.8) | | | <0.001 |
| Self-report diseases |  | |  | |  | | |  |
| Hypertension | 678 (69.3%) | | 186 (67.4%) | | 864 (68.8%) | | | 0.61 |
| Dyslipidemia | 388 (39.6%) | | 113 (40.9%) | | 501 (39.9%) | | | 0.75 |
| Diabetes | 156 (15.9%) | | 52 (18.8%) | | 208 (16.6%) | | | 0.29 |
| Heart Diseases | 90 (9.2%) | | 29 (10.5%) | | 119 (9.5%) | | | 0.59 |
| Systolic blood pressure, mm Hg | 145.9 (21.4) | | 145.7 (25.4) | | 145.9 (22.3) | | | 0.90 |
| Diastolic blood pressure, mm Hg | 79.1 (11.6) | | 77.8 (12.3) | | 78.8 (11.8) | | | 0.09 |
| Health-related quality of life* | 0.82 (0.20) | | 0.74 (0.26) | | 0.80 (0.22) | | | <0.001 |
| Depression status** |  | |  | |  | | | 0.010 |
| Having depressive symptoms | 71 (7.3%) | | 34 (12.3%) | | 105 (8.4%) | | |  |
| Not having depressive symptoms | 908 (92.7%) | | 242 (87.7%) | | 1150 (91.6%) | | |  |
| Timed uo and go, completion time≥14s^†^ | 465 (47.5%) | | 189 (71.1%) | | 654 (52.9%) | | | <0.001 |
| Medication use |  | |  | |  | | |  |
| Antiplatelet | 640 (65.3%) | | 187 (67.8%) | | 827 (65.9%) | | | 0.51 |
| Statin | 257 (26.2%) | | 67 (24.3%) | | 324 (25.8%) | | | 0.56 |
| Antihypertensive medicines | 779 (79.6%) | | 217 (78.6%) | | 996 (79.4%) | | | 0.80 |
| Adherence to medications^‡^ |  | |  | |  | | |  |
| Antiplatelet | 400/640 (62.5%) | | 120/187 (64.2%) | | 520/827 (62.9%) | | | 0.74 |
| Statin | 162/257 (63.0%) | | 41/67 (61.2%) | | 203/324 (62.7%) | | | 0.89 |
| Antihypertensive medicines | 485/779 (62.3%) | | 139/217 (64.1%) | | 624/996 (62.7%) | | | 0.69 |
| Moderate to severe disability^§^ | 240 (24.5%) | | 106 (38.4%) | | 346 (27.6%) | | | <0.001 |
| **Table B. Continued** | | | | | | | | |
| **Characteristics** | **Followed** |  | | **Deceased** |  | **Total** |  | **P value** |
|  | 979 (78.0%) | | 276 (22.0%) | | 1255 (100%) | | |  |
| Whether experienced recurrence in the past year |  | |  | |  | | | 0.001 |
| No | 717 (73.2%) | | 173 (62.7%) | | 890 (70.9%) | | |  |
| Yes | 262 (26.8%) | | 103 (37.3%) | | 365 (29.1%) | | |  |
| Stroke hospitalization in the past year | 190 (19.4%) | | 58 (21.0%) | | 248 (19.8%) | | | 0.61 |
| Data are mean (standard deviation), n (%), or median (Quantile 1-Quantile 3). | | | | | | | | |
| * Health-related quality of life was measured by using EQ5D-5L and was converted into a utility score based on the Chinese value set. The utility score ranged from −0.4 to 1. | | | | | | | | |
| ** Depression status was measure by using 2 items Patient Health Questionnaire, and dichotomised into binary as scores ≥ 2 (indicating with depressive symptoms) versus < 2 (indicating without depressive symptoms). | | | | | | | | |
| ^†^ Timed up and go results were recorded in seconds and dichotomized into binary as ≥ 14 s (indicating poorer limb mobility) versus < 14 s (better limb mobility) based on previous literature. | | | | | | | | |
| ^‡^ Medication agherence was only measured among participants who were taking the specific medicine based on 4-item Morisky Green Levine Scale. | | | | | | | | |
| ^§^ Disability was measured by the modified Rankin Scale, and patients who received a score above 3 were grouped into the “moderate to severe disability” group. | | | | | | | | |

| **Table C. Baseline characteristics by status of follow-up among participants who survived at 70 months (n=1023)** | | | | | | | |
| --- | --- | --- | --- | --- | --- | --- | --- |
| **Characteristics** | **Followed** |  | **Lost to follow-up** |  | **Total** |  | **P value** |
|  | 979 (95.7%) | | 44 (4.3%) | | 1023 (100%) | |  |
| Intervention allocation |  | |  | |  | | 0.31 |
| Control | 489 (49.9%) | | 18 (40.9%) | | 507 (49.6%) | |  |
| Intervention | 490 (50.1%) | | 26 (59.1%) | | 516 (50.4%) | |  |
| Age, years | 64.9 (8.2) | | 62.1 (8.8) | | 64.8 (8.2) | | 0.029 |
| Sex |  | |  | |  | | 0.61 |
| Female | 451 (46.1%) | | 18 (40.9%) | | 469 (45.8%) | |  |
| Male | 528 (53.9%) | | 26 (59.1%) | | 554 (54.2%) | |  |
| Education |  | |  | |  | | 0.42 |
| No schooling | 414 (42.3%) | | 15 (34.1%) | | 429 (41.9%) | |  |
| Some schooling or primary school only | 292 (29.8%) | | 13 (29.5%) | | 305 (29.8%) | |  |
| Above primary school | 273 (27.9%) | | 16 (36.4%) | | 289 (28.3%) | |  |
| Marital status |  | |  | |  | | 1.00 |
| Married | 816 (83.4%) | | 37 (84.1%) | | 853 (83.4%) | |  |
| Widowed, diveorced, or not married | 163 (16.6%) | | 7 (15.9%) | | 170 (16.6%) | |  |
| Annual income, CNY |  | |  | |  | | 0.30 |
| ≤ 5000 | 556 (56.8%) | | 21 (47.7%) | | 577 (56.4%) | |  |
| > 5000 | 423 (43.2%) | | 23 (52.3%) | | 446 (43.6%) | |  |
| Phone ownership |  | |  | |  | | <0.001 |
| No personal phone | 211 (21.6%) | | 8 (18.2%) | | 219 (21.4%) | |  |
| Basic phone | 690 (70.4%) | | 25 (56.8%) | | 715 (69.8%) | |  |
| Smart phone | 78 (8.0%) | | 11 (25.0%) | | 89 (8.7%) | |  |
| Smoking status |  | |  | |  | | 0.048 |
| Current smoker | 154 (15.7%) | | 13 (29.5%) | | 167 (16.3%) | |  |
| Former smoker | 182 (18.6%) | | 8 (18.2%) | | 190 (18.6%) | |  |
| Never smoker | 643 (65.7%) | | 23 (52.3%) | | 666 (65.1%) | |  |
| Health enhancing physical activity |  | |  | |  | | 0.19 |
| Not achieving | 801 (81.8%) | | 32 (72.7%) | | 833 (81.4%) | |  |
| **Table C. Continued** |  | |  | |  | |  |
| **Characteristics** | **Followed** |  | **Lost to follow-up** |  | **Total** |  | **P value** |
|  | 979 (95.7%) | | 44 (4.3%) | | 1023 (100%) | |  |
| Achieving | 178 (18.2%) | | 12 (27.3%) | | 190 (18.6%) | |  |
| Stroke type |  | |  | |  | | 0.21 |
| Ischemic | 844 (86.2%) | | 34 (77.3%) | | 878 (85.8%) | |  |
| Hemorrghage | 132 (13.5%) | | 10 (22.7%) | | 142 (13.9%) | |  |
| Not specified | 3 (0.3%) | | 0 (0.0%) | | 3 (0.3%) | |  |
| Stroke duration, years |  | |  | |  | |  |
| Since the first event | 5.0 (2.1-9.7) | | 6.3 (3.4-0.7) | | 5.1 (2.2-9.7) | | <0.001 |
| Since the last event | 3.3 (1.1-6.7) | | 3.7 (1.8-7.1) | | 3.3 (1.1-6.7) | | 0.001 |
| Self-report diseases |  | |  | |  | |  |
| Hypertension | 678 (69.3%) | | 33 (75.0%) | | 711 (69.5%) | | 0.52 |
| Dyslipidemia | 388 (39.6%) | | 18 (40.9%) | | 406 (39.7%) | | 0.99 |
| Diabetes | 156 (15.9%) | | 8 (15.6%) | | 164 (16.0%) | | 0.85 |
| Heart Diseases | 90 (9.2%) | | 5 (8.2%) | | 95 (9.3%) | | 0.83 |
| Systolic blood pressure, mm Hg | 145.9 (21.4) | | 145.0 (24.2) | | 145.9 (21.5) | | 0.79 |
| Diastolic blood pressure, mm Hg | 79.1 (11.6) | | 80.0 (10.5) | | 79.2 (11.6) | | 0.62 |
| Health-related quality of life* | 0.82 (0.20) | | 0.85 (0.19) | | 0.82 (0.20) | | 0.33 |
| Depression status** |  | |  | |  | | 0.87 |
| Having depressive symptoms | 71 (7.3%) | | 4 (9.1%) | | 75 (7.3%) | |  |
| Not having depressive symptoms | 908 (92.7%) | | 40 (90.9%) | | 948 (92.7%) | |  |
| Timed uo and go, completion time≥14s^†^ | 465 (47.5%) | | 17 (38.6%) | | 482 (47.5%) | | 0.30 |
| Medication use |  | |  | |  | |  |
| Antiplatelet | 640 (65.3%) | | 25 (56.8%) | | 665 (65.0%) | | 0.32 |
| Statin | 257 (26.2%) | | 16 (36.4%) | | 273 (26.7%) | | 0.19 |
| Antihypertensive medicines | 779 (79.6%) | | 34 (77.3%) | | 813 (79.5%) | | 0.86 |
| Adherence to medications^‡^ |  | |  | |  | |  |
| Antiplatelet | 400/640 (62.5%) | | 17/25  (68.0%) | | 417/665 (62.7%) | | 0.73 |
| **Table C. Continued** |  | |  | |  | |  |
| **Characteristics** | **Followed** |  | **Lost to follow-up** |  | **Total** |  | **P value** |
|  | 979 (95.7%) | | 44 (4.3%) | | 1023 (100%) | |  |
| Statin | 162/257 (63.0%) | | 13/16 (81.3%) | | 175/273 (64.1%) | | 0.23 |
| Antihypertensive medicines | 485/779 (62.3%) | | 21/34 (61.8%) | | 506/813 (62.2%) | | 1.00 |
| Moderate to severe disability^§^ | 240 (24.5%) | | 6 (13.6%) | | 246 (24.1%) | | 0.14 |
| Whether experienced recurrence in the past year |  | |  | |  | | 0.82 |
| No | 717 (73.2%) | | 31 (70.5%) | | 748 (73.1%) | |  |
| Yes | 262 (26.8%) | | 13 (29.5%) | | 275 (26.9%) | |  |
| Stroke hospitalization in the past year | 190 (19.4%) | | 8 (18.2%) | | 198 (19.4%) | | 0.99 |
| Data are mean (standard deviation), n (%), or median (Quantile 1-Quantile 3). | | | | | | | |
| * Health-related quality of life was measured by using EQ5D-5L and was converted into a utility score based on the Chinese value set. The utility score ranged from −0.4 to 1. | | | | | | | |
| ** Depression status was measure by using 2 items Patient Health Questionnaire, and dichotomised into binary as scores ≥ 2 (indicating with depressive symptoms) versus < 2 (indicating without depressive symptoms). | | | | | | | |
| ^†^ Timed up and go results were recorded in seconds and dichotomized into binary as ≥ 14 s (indicating poorer limb mobility) versus < 14 s (better limb mobility) based on previous literature. | | | | | | | |
| ^‡^ Medication agherence was only measured among participants who were taking the specific medicine based on 4-item Morisky Green Levine Scale. | | | | | | | |
| ^§^ Disability was measured by the modified Rankin Scale , and patients who received a score above 3 were grouped into the “moderate to severe disability” group. | | | | | | | |

**
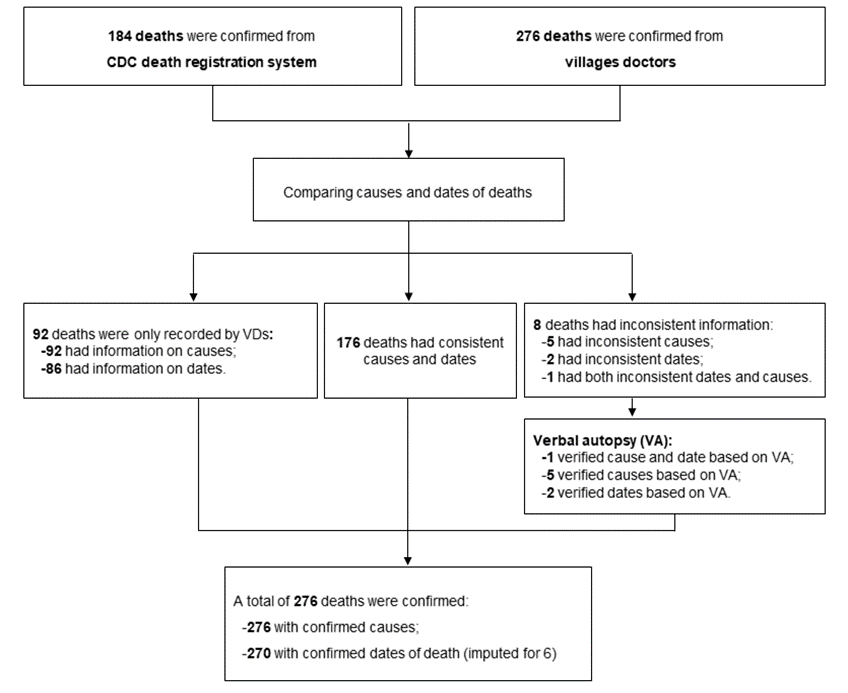
**

**Figure B. Flow-diagram for confirming mortality information from three sources**

| **Table D. Baseline characteristics of participants of the SINEMA trial who died during 70-months follow-up post-baseline (n=276)** | | | | |
| --- | --- | --- | --- | --- |
| **Characteristic** | **Intervention arms** | |  | **Overall (n=276)** |
|  | **Intervention**  **(n=121)** | **Control**  **(n=155)** | |  |
| Age, years | 69.5 (7.3) | 68.6 (7.0) | | 69.0 (7.1) |
| Sex |  |  | |  |
| Female | 30 (24.8%) | 54 (34.8%) | | 84 (30.4%) |
| Male | 91 (75.2%) | 101 (65.2%) | | 192 (69.6%) |
| Education |  |  | |  |
| No schooling | 40 (33.1%) | 69 (44.5%) | | 109 (39.5%) |
| Primary school only | 39 (32.2%) | 43 (27.7%) | | 82 (29.7%) |
| Above primary school | 42 (34.7%) | 43 (27.7%) | | 85 (30.8%) |
| Marital status |  |  | |  |
| Married | 15 (12.4%) | 39 (25.2%) | | 222 (80.4%) |
| Widowed, diveorced,  or not married | 106 (87.6%) | 116 (74.8%) | | 54 (19.6%) |
| Phone ownership |  |  | |  |
| No personal phone | 42 (34.7%) | 62 (40.0%) | | 104 (37.7%) |
| Basic phone | 74 (61.2%) | 86 (55.5%) | | 160 (58.0% ) |
| Smart phone | 5 (4.1%) | 7 (4.5%) | | 12 (4.3%) |
| Smoking status |  |  | |  |
| Current smoker | 20 (16.5%) | 34 (21.9%) | | 54 (19.6%) |
| Former smoker | 38 (31.4%) | 34 (21.9%) | | 72 (26.1%) |
| Never smoker | 63 (52.1%) | 87 (56.1%) | | 150 (54.3%) |
| Body mass index (BMI), kg/m^2^ | 24.6 (3.7) | 24.4 (3.8) | | 24.4 (3.8) |
| Waist circumference, cm | 91.6 (10.0) | 91.0 (9.9) | | 91.3 (9.9) |
| Stroke type |  |  | |  |
| Ischemic | 109 (90.1%) | 132 (85.2%) | | 241 (87.3%) |
| Hemorrghage | 12 (9.9%) | 22 (14.2%) | | 34 (12.3%) |
| Not specified | 0 (0%) | 1 (0.6%) | | 1 (0.4%) |
| **Table D. Continued** |  |  | |  |
| **Characteristic** | **Intervention arms** | |  | **Overall (n=276)** |
|  | **Intervention**  **(n=121)** | **Control**  **(n=155)** | |  |
| Stroke duration, years |  |  | |  |
| Since the first event | 6.1 (3.1-10.3) | 5.9 (3.0-11.6) | | 5.9 (3.0-11.1) |
| Since the last event | 3.5 (0.7-7.1) | 3.0 (1.1-7.4) | | 3.2 (1.0-7.3) |
| Self-report diseases |  |  | |  |
| Hypertension | 93 (76.9%) | 93 (60.0%) | | 186 (67.4%) |
| Dyslipidemia | 52 (43.0%) | 61 (39.4%) | | 113 (40.9%) |
| Diabetes | 26 (21.5%) | 26 (16.8%) | | 52 (18.8%) |
| Heart Diseases | 17 (14.0%) | 12 (7.7%) | | 29 (10.5%) |
| Medication use |  |  | |  |
| Antiplatelet | 85 (70.2%) | 102 (65.8%) | | 187 (67.8%) |
| Statin | 31 (25.6%) | 36 (23.2%) | | 67 (24.3%) |
| Antihypertensive medicines | 102 (84.3%) | 115 (74.2%) | | 217 (78.6%) |
| Adherence to medications^*^ |  |  | |  |
| Antiplatelet | 56/85 (65.9%) | 64/102 (62.7%) | | 120/187 (64.2%) |
| Statin | 18/31 (58.1%) | 23/36 (63.9%) | | 41/67 (61.2%) |
| Antihypertensive medicines | 60/102 (58.8%) | 79/115 (68.7%) | | 139/217 (64.1%) |
| Physical activity, metabolic equivalents minutes per week | 693.0  (198.0-1848.0) | 693.0  (0.0-1386.0) | | 693.0  (49.5-1543.5) |
| Systolic blood pressure, mm Hg | 147.0 (20.5) | 144.7 (28.7) | | 145.7 (25.4) |
| Diastolic blood pressure, mm Hg | 77.1 (11.1) | 78.3 (13.1) | | 77.8 (12.3) |
| Timed up and go,  completion time≥14s^†^ | 83 (68.6%) | 106 (68.4%) | | 189 (71.1%) |
| Moderate to severe disability^‡^ | 42 (34.7%) | 64 (41.3%) | | 106 (38.4%) |
| Stroke hospitalization in the past 12 months | 27 (22.3%) | 31 (20.0%) | | 58 (21.0%) |
| Data are n (%), n/N (%), mean (standard deviation), or median (Quantile1-Quantile3). | | | | |
| * Medication adherence was only measured among participants who were taking the specific medicine and was based on 4-item Morisky Green Levine Scale. | | | | |
| ^†^ Timed up and go results were recorded in seconds and dichotomised into binary as ≥ 14 s (indicating poorer limb mobility) versus < 14 s (better limb mobility). | | | | |
| ^‡^ Disability was measured by the modified Rankin Scale , and patients who received a score above 3 were grouped into the “moderate to severe disability” group. | | | | |

| **Table E. Baseline characteristics by intervention arms of participants who survived at 70-months post-baseline (n=1023)** | | | | |
| --- | --- | --- | --- | --- |
| **Characteristic** | **Intervention arms** | |  | **Overall (n=1023)** |
|  | **Intervention**  **(n=516)** | **Control**  **(n=507)** | |  |
| Age, years | 65.4 (8.2) | 64.2 (8.2) | | 64.8 (8.2) |
| Sex |  |  | |  |
| Female | 242 (46.9%) | 227 (44.8%) | | 469 (45.8%) |
| Male | 274 (53.1%) | 280 (55.2%) | | 554 (54.2%) |
| Education |  |  | |  |
| No schooling | 224 (43.4%) | 205 (40.4%) | | 429 (41.9%) |
| Primary school only | 143 (27.7%) | 162 (32.0%) | | 305 (29.8%) |
| Above primary school | 149 (28.9%) | 140 (27.6%) | | 289 (28.3%) |
| Marital status |  |  | |  |
| Married | 420 (81.4%) | 433 (85.4%) | | 853 (83.4%) |
| Widowed, diveorced,  or not married | 96 (18.6%) | 74 (14.6%) | | 170 (16.6%) |
| Phone ownership |  |  | |  |
| No personal phone | 122 (23.6%) | 97 (19.1%) | | 219 (21.4%) |
| Basic phone | 361 (70.0%) | 354 (69.8%) | | 715 (69.9%) |
| Smart phone | 33 (6.4%) | 56 (11.0%) | | 89 (8.7%) |
| Smoking status |  |  | |  |
| Current smoker | 79 (15.3%) | 88 (17.4%) | | 167 (16.3%) |
| Former smoker | 92 (17.8%) | 98 (19.3%) | | 190 (18.6%) |
| Never smoker | 345 (66.9%) | 321 (63.3%) | | 666 (65.1%) |
| Body mass index (BMI), kg/m^2^ | 25.8 (3.6) | 25.8 (3.5) | | 25.8 (3.6) |
| Waist circumference, cm | 93.4 (9.9) | 93.8 (10.0) | | 93.6 (9.9) |
| Stroke type |  |  | |  |
| Ischemic | 446 (86.4%) | 432 (85.2%) | | 878 (85.8%) |
| Hemorrghage | 68 (13.2%) | 74 (14.6%) | | 142 (13.9%) |
| Not specified | 2 (0.4%) | 1 (0.2%) | | 3 (0.3%) |
| **Table E. Continued** |  |  | |  |
| **Characteristic** | **Intervention arms** | |  | **Overall (n=1023)** |
|  | **Intervention**  **(n=516)** | **Control**  **(n=507)** | |  |
| Stroke duration, years |  |  | |  |
| Since the first event | 5.0 (2.1-9.7) | 5.1 (2.3-9.7) | | 5.1 (2.2-9.7) |
| Since the last event | 3.4 (1.1-6.7) | 3.3 (1.2-6.7) | | 3.3 (1.1-6.7) |
| Self-report diseases |  |  | |  |
| Hypertension | 368 (71.3%) | 343 (67.7%) | | 711 (69.5%) |
| Dyslipidemia | 196 (38.0%) | 210 (41.4%) | | 406 (39.7%) |
| Diabetes | 87 (16.9%) | 77 (15.2%) | | 164 (16.0%) |
| Heart Diseases | 53 (10.3%) | 42 (8.3%) | | 95 (9.3%) |
| Medication use |  |  | |  |
| Antiplatelet | 318 (62.7%) | 347 (67.2%) | | 665 (65.0%) |
| Statin | 146 (28.8%) | 127 (24.6%) | | 273 (26.7%) |
| Antihypertensive medicines | 393 (77.5%) | 420 (81.4%) | | 813 (79.5%) |
| Adherence to medications^*^ |  |  | |  |
| Antiplatelet | 198/318 (62.3%) | 219/347 (63.1%) | | 417/655 (62.7%) |
| Statin | 87/146 (59.6%) | 88/127 (69.3%) | | 175/273 (64.1%) |
| Antihypertensive medicines | 237/393 (60.3%) | 269/420 (64.0%) | | 506/813 (62.2%) |
| Physical activity, metabolic equivalents minutes per week | 1386.0  (462.0-2772.0) | 1302.0  (388.2-2959.5) | | 1386.0  (438.5-2772.0) |
| Systolic blood pressure, mm Hg | 144.5 (21.0) | 144.5 (22.0) | | 145.9 (21.5) |
| Diastolic blood pressure, mm Hg | 78.2 (11.7) | 80.2 (11.3) | | 79.2 (11.5) |
| Timed up and go,  completion time≥14s^†^ | 241 (46.7%) | 241 (47.5%) | | 482 (47.5%) |
| Moderate to severe disability^‡^ | 137 (26.6%) | 109 (21.5%) | | 246 (24.1%) |
| Stroke hospitalization in the past 12 months | 97 (18.8%) | 101 (19.9%) | | 198 (19.4%) |
| Data are n (%), n/N (%), mean (standard deviation), or median (Quantile1-Quantile3). | | | | |
| * Medication adherence was only measured among participants who were taking the specific medicine and was based on 4-item Morisky Green Levine Scale. | | | | |
| ^†^ Timed up and go results were recorded in seconds and dichotomised into binary as ≥ 14 s (indicating poorer limb mobility) versus < 14 s (better limb mobility). | | | | |
| ^‡^ Disability was measured by the modified Rankin Scale , and patients who received a score above 3 were grouped into the “moderate to severe disability” group. | | | | |

| **Table F. Baseline characteristics of participants in the SINEMA trial by vital status at 70-months post-baseline (n=1299)** | | | |
| --- | --- | --- | --- |
| **Characteristic** | **Vital status*** | | **Overall (n=1299)** |
|  | **Survivors**  **(n=1023)** | **Decedents (n=276)** |  |
| Age, years | 64.8 (8.2) | 69.0 (7.1) | 65.7 (8.2) |
| Sex |  |  |  |
| Female | 469 (45.8%) | 84 (30.4%) | 553 (42.6%) |
| Male | 554 (54.2%) | 192 (69.6%) | 746 (57.4%) |
| Education |  |  |  |
| No schooling | 429 (41.9%) | 109 (39.5%) | 538 (41.4%) |
| Primary school only | 305 (29.8%) | 82 (29.7%) | 387 (29.8%) |
| Above primary school | 289 (28.3%) | 85 (30.8%) | 374 (28.8%) |
| Marital status |  |  |  |
| Married | 853 (83.4%) | 222 (80.4%) | 1075 (82.8%) |
| Widowed, diveorced,  or not married | 170 (16.6%) | 54 (19.6%) | 224 (17.2%) |
| Phone ownership |  |  |  |
| No personal phone | 219 (21.4%) | 104 (37.7%) | 323 (24.9%) |
| Basic phone | 715 (69.9%) | 160 (58.0% ) | 875 (67.4%) |
| Smart phone | 89 (8.7%) | 12 (4.3%) | 101 (7.8%) |
| Smoking status |  |  |  |
| Current smoker | 167 (16.3%) | 54 (19.6%) | 221 (17.0%) |
| Former smoker | 190 (18.6%) | 72 (26.1%) | 262 (20.2%) |
| Never smoker | 666 (65.1%) | 150 (54.3%) | 816 (62.8%) |
| Body mass index (BMI), kg/m^2^ | 25.8 (3.6) | 24.4 (3.8) | 25.5 (3.7) |
| Waist circumference, cm | 93.6 (9.9) | 91.3 (9.9) | 93.1 (10.0) |
| Stroke type |  |  |  |
| Ischemic | 878 (85.8%) | 241 (87.3%) | 1119 (86.1%) |
| Hemorrghage | 142 (13.9%) | 34 (12.3%) | 176 (13.5%) |
| Not specified | 3 (0.3%) | 1 (0.4%) | 4 (0.3%) |
| **Table F. Continued** | | | |
| **Characteristic** | **Vital status*** | | **Overall (n=1299)** |
|  | **Survivors**  **(n=1023)** | **Decedents (n=276)** |  |
| Stroke duration, years |  |  |  |
| Since the first event | 5.1 (2.2-9.7) | 5.9 (3.0-11.1) | 5.3 (2.3-9.8) |
| Since the last event | 3.3 (1.1-6.7) | 3.2 (1.0-7.3) | 3.3 (1.1-6.8) |
| Self-report diseases |  |  |  |
| Hypertension | 711 (69.5%) | 186 (67.4%) | 897 (69.1%) |
| Dyslipidemia | 406 (39.7%) | 113 (40.9%) | 519 (40.0%) |
| Diabetes | 164 (16.0%) | 52 (18.8%) | 216 (16.6%) |
| Heart Diseases | 95 (9.3%) | 29 (10.5%) | 124 (9.5%) |
| Medication use |  |  |  |
| Antiplatelet | 665 (65.0%) | 187 (67.8%) | 852 (65.6%) |
| Statin | 273 (26.7%) | 67 (24.3%) | 340 (26.2%) |
| Antihypertensive medicines | 813 (79.5%) | 217 (78.6%) | 1030 (79.3%) |
| Adherence to medications^†^ |  |  |  |
| Antiplatelet | 417/655 (62.7%) | 120/187 (64.2%) | 537/852 (63.0%) |
| Statin | 175/273 (64.1%) | 41/67 (61.2%) | 216/340 (63.5%) |
| Antihypertensive medicines | 506/813 (62.2%) | 139/217 (64.1%) | 645/1030 (62.6%) |
| Physical activity, metabolic equivalents minutes per week | 1386.0  (438.5-2772.0) | 693.0  (49.5-1543.5) | 1164.0  (346.5-2658.0) |
| Systolic blood pressure, mm Hg | 145.9 (21.5) | 145.7 (25.4) | 145.9 (22.4) |
| Diastolic blood pressure, mm Hg | 79.2 (11.5) | 77.8 (12.3) | 78.9 (11.7) |
| Timed up and go,  completion time≥14s^‡^ | 482 (47.5%) | 189 (71.1%) | 671 (52.4%) |
| Moderate to severe disability**^§^** | 246 (24.1%) | 106 (38.4%) | 352 (27.1%) |
| Stroke hospitalization in the past 12 months | 198 (19.4%) | 58 (21.0%) | 256 (19.7%) |
| Data are n (%), n/N (%), mean (standard deviation), or median (Quantile1-Quantile3). | | | |
| * Vital status was ascertained according to standard procedures at 70 months after baseline, up to April 30, 2023. Participants who died before this date were considered decedents; otherwise, they were considered survivors. | | | |
| ^†^ Medication adherence was only measured among participants who were taking the specific medicine and was based on 4-item Morisky Green Levine Scale. | | | |
| ^‡^ Timed up and go results were recorded in seconds and dichotomised into binary as ≥ 14 s (indicating poorer limb mobility) versus < 14 s (better limb mobility). | | | |
| ^§^ Disability was measured by the modified Rankin Scale , and patients who received a score above 3 were grouped into the “moderate to severe disability” group. | | | |

| **Table G. The adjusted hazard ratios (95% confidence intervals) of intervention on mortality in patients experiencing stroke at 70-month follow-up using sub-distribution model.** | | | | |
| --- | --- | --- | --- | --- |
| **Outcomes** | **Minimally adjusted HR (95% CI) *** | **p value** | **Fully adjusted HR (95% CI) **** | **p value** |
| **All-cause mortality** | 0·73 (0.59, 0.90) | 0.004 | 0.72 (0.58, 0.90) | 0.004 |
| **Cardiovascular cause-specific mortality^†^** | 0.75 (0.59, 0.96) | 0.024 | 0.74 (0.58, 0.96) | 0.022 |
| Stroke | 0.73 (0.45, 1.18) | 0.21 | 0.77 (0.47, 1.26) | 0.30 |
| Non-stroke | 0.78 (0.54, 1.11) | 0.17 | 0.74 (0.52, 1.05) | 0.09 |
| **Other cause of mortality^‡^** | 0.72 (0.47, 1.10) | 0.13 | 0.73 (0.47, 1.13) | 0.16 |
| ^*^ HRs were adjusted for town, age, and sex. | | | | |
| ^**^ HRs were adjusted for town, age, sex, and variables noted to be differential by intervention arms at baseline (baseline diastolic blood pressure, having hypertension, having none of home assets [TV, refrigerator, air conditioner, and computer], and taking antihypertensive medicines).  CI= confidential interval, HR= hazard ratio. | | | | |
| ^†^ The classification of cardiovascular cause-specific mortality is based on the Global Burden of Diseases, Injuries, and Risk Factors Study, where death due to any type of stroke is classified as such.  ^‡^ The specific other causes of mortality are detailed in appendix pp 3-5. | | | | |

**
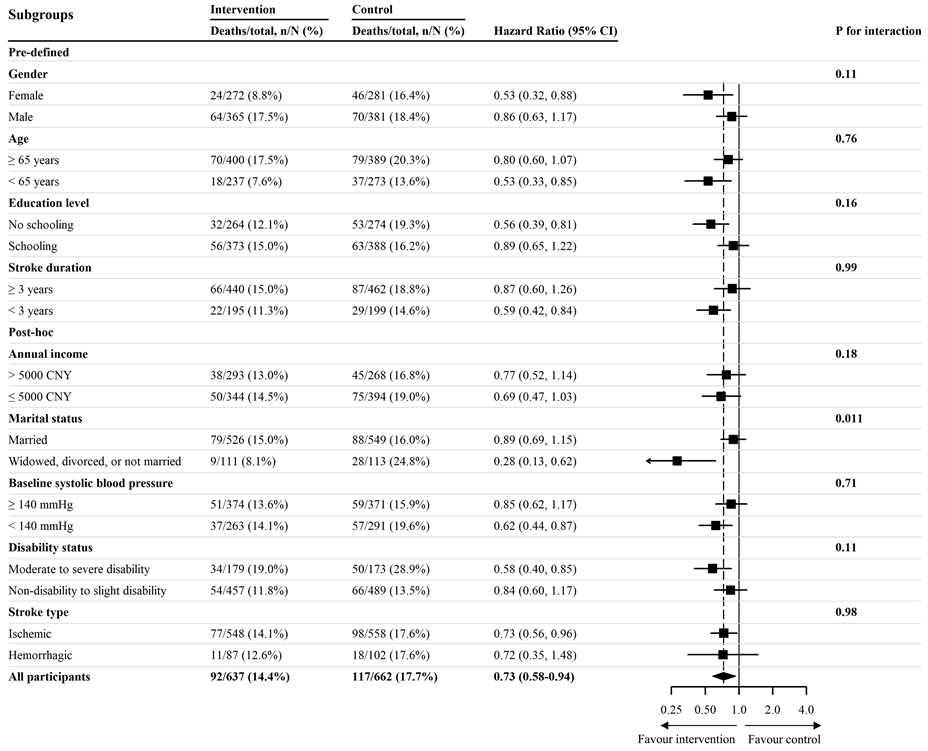
** **Figure C. Long-term outcomes on cardiovascular cause-specific mortality in each pre-specified and post-hoc subgroup, minimally adjusted model**

CI= confidence interval. Hazard ratios were adjusted for age, sex, and township, with villages considered as clusters.

**
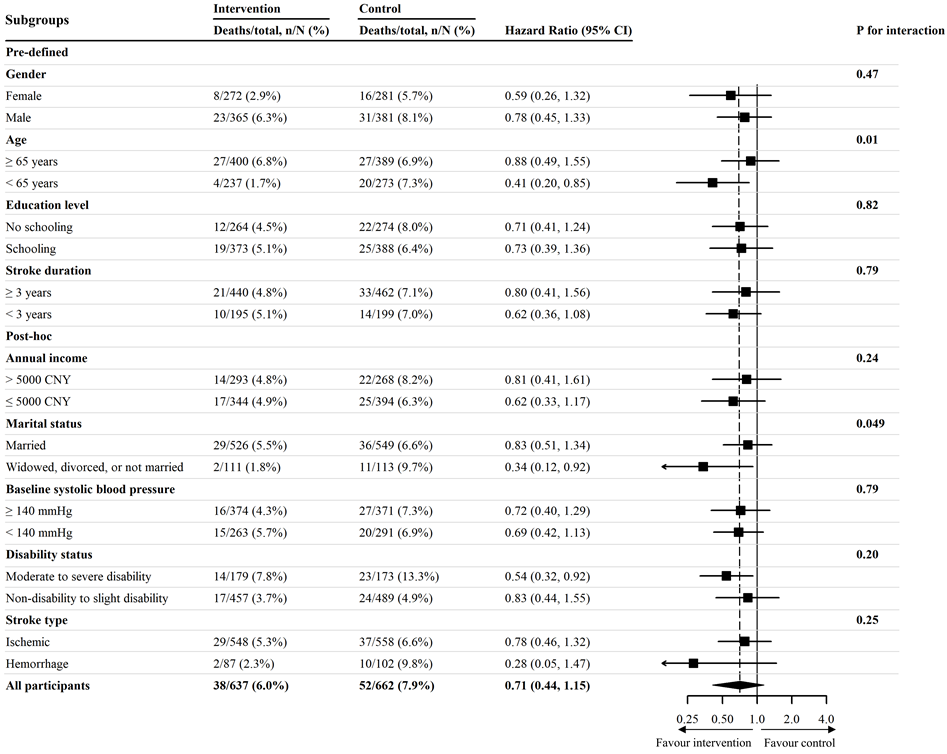
** **Figure D. Long-term outcomes on stroke cause-specific mortality in each pre-specified and post-hoc subgroup, minimally adjusted model**

CI= confidence interval. Hazard ratios were adjusted for age, sex, and township, with villages considered as clusters.

| **Table H. Effects (risk difference) of the SINEMA intervention on intermediate outcomes from baseline to 70 months among living patients (n=979)** | | | | | |
| --- | --- | --- | --- | --- | --- |
| **Outcomes** | **Intervention arms** | |  | **Minimally adjusted model^*^** | **Fully adjusted model^**^** |
|  | **Intervention (n=490)** | **Control (n=489)** | | **Estimate (95% CI)^†^** | **Estimate (95% CI)^†^** |
| **Medication adherence**^‡^ |  |  | |  |  |
| Antiplatelets | 64.9% (198) | 59.4% (174) | | 5.7pp (-2.8, 14.3) | 5.1pp (-3.9, 14.1) |
| Statins | 63.9% (131) | 61.0% (136) | | 4.5pp (-6.7, 15.7) | 4.5pp (-5.9, 14.8) |
| Antihypertensive medicines | 67.0% (286) | 61.0% (246) | | 6.2pp (0, 12.6) | 5.0pp (-1.3, 11.3) |
| **Physical activity and functionning** |  |  | |  |  |
| Timed up and go ≥14 seconds**^§^** | 62.7% (279) | 59.9% (266) | | 2.1pp (-4.3, 8.5) | 1.6pp (-4.9, 7.7) |
| Data are % (n). CI=confidence interval. pp=percentage points. | | | |  |  |
| * Minimally adjusted model: Adjusted for baseline outcome, township, sex, age, and interview month; removing outliers (2 for systolic blood pressure, 6 for diastolic blood pressure) in the outcome variable (based on a priori decision to remove those that are more than 2 interquartile ranges above the third quartile or below the first quartile). | | | | | |
| ** Fully adjusted model: Adjusted for baseline outcome, township, sex, age, interview month, variables noted to be differential by treatment arm at baseline (p<0.05; baseline diastolic blood pressure, having hypertension, having none of home assets [TV, refrigerator, air conditioner, and computer], and taking antihypertensive medicines), and baseline variables related to death (health-related quality of life, whether achieve health enhancing physical activity, stroke recurrence, disability, depression status, and completion time of timed up and go test) and loss-to-follow-up among living patients (phone ownership, smoking statu, and stroke duration); removing the same outliers as in the minimally adjusted model. | | | | | |
| ^†^ “Estimate” refers to the risk difference (control arm is the reference). | | | | | |
| **^‡^** Medication adherence refers to a perfect adherence with score of 0 based on the 4-item Morisky Green Levine Scale. Medication adherence was only measured among participants who were taking antiplatelets (intervention n=305, control=293), statins (n=205, n=223), and antihypertensive medicines (n=427, n=403). Medication adherence outcomes were not adjusted for baseline outcome, since the set of participants taking a given medication at baseline was not the same set taking the medicines at the post-trial follow-up. | | | | | |
| **^§^** “Timed up and go test” results were recorded in seconds during measurement and dichotomised into binary as ≥ 14 seconds (indicating poorer limb mobility) versus < 14 seconds (better limb mobility). | | | | | |

| **Table I.** **Participants characteristics and intermediate outcomes at baseline and at the long-term follow-up (n=979)** | | | | | | | | |
| --- | --- | --- | --- | --- | --- | --- | --- | --- |
| **Characteristic** | **Baseline assessment** | | |  | **Long-term follow-up assessment** | | |  |
|  | **Intervention**  **(n=490)** | **Control**  **(n=489)** | **Overall**  **(n=979)** | | **Intervention**  **(n=490)** | **Control**  **(n=489)** | **Overall**  **(n=979)** | |
| Marital status |  |  |  | |  |  |  | |
| Married | 400 (81.6%) | 416 (85.1%) | 816 (83.4%) | | 369 (75.3%) | 388 (79.3%) | 757 (77.3%) | |
| Widowed, diveorced,  or not married | 90 (18.4%) | 73 (14.9%) | 163 (16.6%) | | 121 (24.7%) | 101 (20.7%) | 222 (22.7%) | |
| Phone ownership |  |  |  | |  |  |  | |
| No personal phone | 94 (19.2%) | 117 (23.9%) | 211 (21.6%) | | 111 (22.7%) | 115 (23.5%) | 226 (23.1%) | |
| Basic phone | 344 (70.3%) | 346 (70.6%) | 690 (70.5%) | | 267 (54.5%) | 250 (51.1%) | 517 (52.8%) | |
| Smart phone | 51 (10.4%) | 27 (5.5%) | 78 (8.0%) | | 112 (22.9%) | 124 (25.4%) | 236 (24.1%) | |
| Smoking status |  |  |  | |  |  |  | |
| Current smoker | 72 (14.7%) | 82 (16.8%) | 154 (15.7%) | | 67 (13.7%) | 80 (16.4%) | 147 (15.0%) | |
| Former smoker | 87 (17.8%) | 95 (19.4%) | 182 (18.6%) | | 103 (21.0%) | 106 (21.7%) | 209 (21.3%) | |
| Never smoker | 331 (67.6%) | 312 (63.8%) | 643 (65.7%) | | 320 (65.3%) | 303 (62.0%) | 623 (63.6%) | |
| Body mass index (BMI), kg/m^2^ | 25.7 (3.6) | 25.8 (3.6) | 25.8 (3.6) | | 26.4 (3.7) | 26.7 (4.7) | 26.5 (4.2) | |
| Waist circumference, cm | 93.4 (9.9) | 93.8 (10.0) | 93.6 (10.0) | | 96.0 (10.4) | 96.4 (9.8) | 96.2 (10.1) | |
| Self-report diseases |  |  |  | |  |  |  | |
| Hypertension | 349 (71.2%) | 329 (67.3%) | 678 (69.3%) | | 429 (87.6%) | 422 (86.3%) | 851 (86.9%) | |
| Dyslipidemia | 186 (38.0%) | 202 (41.3%) | 388 (39.6%) | | 240 (49.0%) | 262 (53.6%) | 502 (51.3%) | |
| **Table I. Continued** |  |  |  | |  |  |  | |
| **Characteristic** | **Baseline assessment** | | |  | **Long-term follow-up assessment** | | |  |
|  | **Intervention**  **(n=490)** | **Control**  **(n=489)** | **Overall**  **(n=979)** | | **Intervention**  **(n=490)** | **Control**  **(n=489)** | **Overall**  **(n=979)** | |
| Diabetes | 83 (16.9%) | 73 (14.9%) | 156 (15.9%) | | 100 (20.4%) | 105 (21.5%) | 205 (20.9%) | |
| Heart Diseases | 49 (10.0%) | 41 (8.4%) | 90 (9.2%) | | 58 (11.8%) | 53 (10.8%) | 111 (11.3%) | |
| Medication use |  |  |  | |  |  |  | |
| Antiplatelet | 333 (68.0%) | 307 (62.8%) | 640 (65.4%) | | 305 (62.2%) | 293 (59.9%) | 598 (61.1%) | |
| Statin | 119 (24.3%) | 138 (28.2%) | 257 (26.3%) | | 205 (41.8%) | 223 (45.6%) | 428 (43.7%) | |
| Antihypertensive medicines | 400 (81.6%) | 379 (77.5%) | 779 (79.6%) | | 427 (87.1%) | 403 (82.4%) | 830 (84.8%) | |
| Adherence to medications^*^ |  |  |  | |  |  |  | |
| Antiplatelet | 209/333 (62.8%) | 191/307 (62.2%) | 400/640 (62.5%) | | 198/305 (64.9%) | 174/293 (59.4%) | 372/598 (62.2%) | |
| Statin | 81/119 (68.1%) | 81/138 (58.7%) | 162/257 (63.0%) | | 131/205 (63.9%) | 136/223 (61.0%) | 267/428 (62.4%) | |
| Antihypertensive medicines | 254/400 (63.5%) | 231/379 (60.9%) | 485/779 (62.3%) | | 286/427 (67.0%) | 246/403 (61.0%) | 532/830 (64.1%) | |
| Physical activity, metabolic equivalents minutes per week | 1386.0  (462.0-2772.0) | 1223.0  (346.5-2784.0) | 1386.0  (396.0-2772.0) | | 693.0  (66.0-2054.0) | 813.0  (247.5-2310.0) | 693.0  (66.0-2079.0) | |
| Systolic blood pressure, mm Hg | 146 (20.8) | 146 (22.0) | 146 (21.4) | | 141.5 (19.8) | 144.0 (21.5) | 142.7 (20.7) | |
| Diastolic blood pressure, mm Hg | 78.2 (11.8) | 80.1 (11.3) | 79.1 (11.6) | | 81.5 (11.0) | 83.5 (11.8) | 82.4 (11.5) | |
| Timed up and go,  completion time≥14s^†^ | 227 (46.3%) | 238 (48.7%) | 465 (47.5%) | | 279 (56.9%) | 266 (54.4%) | 545 (55.7%) | |
| Moderate to severe disability^‡^ | 132 (26.9%) | 108 (22.1%) | 240 (24.5%) | | 159 (32.4%) | 157 (32.1%) | 316 (32.3%) | |
| **Table I. Continued** |  |  |  | |  |  |  | |
| **Characteristic** | **Baseline assessment** | | |  | **Long-term follow-up assessment** | | |  |
|  | **Intervention**  **(n=490)** | **Control**  **(n=489)** | **Overall**  **(n=979)** | | **Intervention**  **(n=490)** | **Control**  **(n=489)** | **Overall**  **(n=979)** | |
| Stroke hospitalization in the past 12 months | 94 (19.2%) | 96 (19.6%) | 190 (19.4%) | | 45 (9.2%) | 41 (8.4%) | 86 (8.8%) | |
| Data are n (%), n/N (%), mean (standard deviation), or median (Quantile1-Quantile3). | | | | | | | | |
| * Medication adherence was only measured among participants who were taking the specific medicine and was based on 4-item Morisky Green Levine Scale. | | | | | | | | |
| ^†^ Timed up and go results were recorded in seconds and dichotomised into binary as ≥ 14 s (indicating poorer limb mobility) versus < 14 s (better limb mobility). | | | | | | | | |
| ^‡^ Disability was measured by the modified Rankin Scale , and patients who received a score above 3 were grouped into the “moderate to severe disability” group. | | | | | | | | |
