# Supplementary material for: Long-term mortality outcome of a primary care-based mobile health intervention for stroke management: Six-year follow-up of a cluster-randomized controlled trial
Source: PLoS Med. 2025 Mar 27;22(3):e1004564. doi: 10.1371/journal.pmed.1004564 (PMC11949329; doi:10.1371/journal.pmed.1004564)
Supplement: Attachment — Submitted filename: Response letter-PLOS Med.docx [file pmed.1004564.s004.docx]

Alexandra Tosun, PhD
Associate Editor - PLOS Medicine
Manuscript no.: MEDICINE-D-24-03550R1

Dec 20, 2024

Dear Dr. Tosun,

Thank you for your and the reviewers’ valuable comments and suggestions on our manuscript entitled “**Long-term effect on mortality of a primary care-based mobile health intervention for stroke: six-year follow-up of a cluster-randomized controlled trial.**” We appreciate the opportunity to revise and improve our manuscript and have carefully addressed the concerns raised by you and the reviewers. Please find our item-by-item responses to each comment raised by you and the reviewers. We have also submitted a clean version of the revised manuscript. We believe that the manuscript has been significantly improved and sincerely hope it will now meet the standards for publication in ***PLOS Medicine***.

Lijing L. Yan, PhD, MPH, and Enying Gong, PhD, *on behalf of all authors*

-----------------------------------------------------------
**Comments from the editorial team:**

*1) In your response regarding study affiliation, you stated that the current study is an extension of the SINEMA study, but also that the current manuscript is part of the SaFaRI study. Please clarify this and ensure that it is clearly described in the Methods section.*

**Response:** We have clarified this point in the study design and participants section of the Methods section (**pp 8, lines 157-162**) as follows: “This study is part of the Stroke Patients and Family Longitudinal Study in Rural China (SaFaRI) (clincailtrial.gov registration number: NCT05792618)), a long-term passive (observational) follow-up of participants and their spouse in the SINEMA cRCT (S1 Study protocol). The original SINEMA trial (clinicaltrial registration number: NCT03185858) was designed to evaluate a primary care-based mobile health intervention for stroke management within one year with protocol published previously [5].”

*2) In the study protocol you stated that the secondary outcomes of interest will be in line with the original protocol. We are aware that some of the long-term outcomes have already been reported in a separate publication. We ask you to transparently list all outcomes in the Methods section and describe which outcomes are reported in the current submission, have been or will be reported elsewhere. In line with this, it will be important to clearly describe which outcomes were pre-specified and which were added post hoc. We would also encourage you to include all remaining outcomes, such as health-related quality of life.*

**Response:** We have revised the outcomes section and detailed all the pre-specified outcomes of the follow-up study. In general, this paper focused on the effectiveness of mortality as pre-specified co-primary outcome and relevant intermediate outcomes. The findings on systolic blood pressure and BP control related outcomes have been reported elsewhere and a few secondary outcomes in cognitive impairment and mental health as major interests in long-term follow-up study will also be report separately.

Please refer to the relevant revision in the **Outcomes section in page 11**, as follows: “The primary outcome of this long-term follow-up study followed the original SINEMA trial in systolic blood pressure but also consider all-cause mortality as pre-specified long-term co-primary outcomes as described in the study protocol (S1 Study protocol). The secondary outcomes include diastolic blood pressure, medication adherence (measured using the 4-item Morisky Green Levine Scale), physical activity level (measured by the short version of the International Physical Activity Questionnaire), mobility (measured using the Timed-Up and Go test), health-related quality of life (measured using the European Quality of Life 5 Dimensions 5 Level Version), disability (measured using the modified Rankin Scale), stroke recurrence (measured by self-reported experience of stroke recurrence), stroke-related hospitalization (measured by self-reported history of hospitalization) and other potential complications (such as cognition impairment).

This paper considered all-cause mortality as primary outcome. Causes of deaths were classified by using ICD 10 codes based on the Global Burden of Diseases, Injuries, and Risk Factors Study into cardiovascular diseases (including stroke and non-stroke cardiovascular diseases) and other causes (Table A in S2 Text) [13]. We also illustrate a subset of secondary outcomes as major intermediate outcomes of mortality, including changes in blood pressure, medication adherence to antiplatelet, statin and antihypertensive medications, changes in physical activity, functioning, disability and stroke hospitalization measured through follow-up surveys. Definitions and measurement of these outcomes have been specified previously and in Appendix A in S2 Text [5]. A detailed analysis in systolic blood pressure and blood pressure control related outcomes in medication use and stroke recurrence have recently been reported separately [12] and changes in cognition and mental health status will also be reported separately.”

-----------------------------------------------------------
Comments from the reviewers:

***Reviewer #1: Statistical review***

This paper reports a long-term follow-up of a cluster randomised trial which assessed a primary-care intervention. The study shows the intervention had long-term beneficial effects on a number of important outcomes. Overall the methods used are appropriate and the results reported well. I have a few comments which I have listed below:

*1.      Abstract: the abstract mentions a number of subgroup analyses. Some of these were posthoc according to the methods, so I would generally recommend not to mention them in the abstract (or at least label them as so). It is more appropriate to include the pre-specified subgroups in the abstract but it seems to me that none of these had significant interactions: I'd therefore not recommend highlighting any of the subgroup analyses in the abstract unless I got any of the above wrong.*

**Response:** Thank you for your suggestion. We have removed the description of subgroup analysis from the abstract.

*2.      Methods: the registration page for the long-term follow-up (NCT05792618) mentions a lot of outcomes that aren't reported here - it might be good to clarify that this paper is not reporting those outcomes.*

**Response:** Thank you for your comment. The registration is also for observational purpose of long-term follow-up of both participants and their spouse. Please see the details of the protocol in Supplement file and we have also clarified all plans for reporting the outcomes in the revised version of the outcome section. Please refers to the response to Q2 by editorial team.

*3.      Methods/results: I did not follow how the number of survivors was so similar in each arm in the intermediate outcome analysis given that there was a difference in mortality between groups - can this be explained?*

**Response:** Thank you for this comment. We have moved the flow diagram from the supplement file to Figure 1 to clarify the number of participants at each wave of follow-up. As shown in **Figure 1**, 662 and 637 participants were randomly allocated into the control and intervention arm respectively. By the end of the one-year intervention, 19 participants in the control arm and 11 participants in the intervention arm had died. During the long-term follow-up period, an additional 136 participants in the control arm and 110 participants in the intervention arm died, leaving 507 and 516 survivors in the control and intervention arm respectively. Among these survivors, 18 and 26 survivors in the control and intervention arm refused to participate in the face-to-face survey, thus leaving 489 and 490 participants in the control and intervention arm respectively in the analysis.

***Reviewer #2:*** *This is a study reporting post-intervention long-term outcomes of a 12-month intervention namely system-integrated technology-enabled model of care for stroke survivors. Up to 70 months of follow-up revealed the SINEMA intervention reduced all-cause mortality, and cardiovascular mortality than the control arm. This is a well written paper with important findings that are particularly relevant to low income regions of the globe.*

**Response:** Thank you for your positive evaluation on our manuscript.

Comments
None

***Reviewer #3***: In the manuscript "Long-term effect on mortality of a primary care-based mobile health intervention for stroke: six-year follow-up of a cluster-randomized controlled trial" the authors have evaluated the long-term (70 months) effect of a primary care-based integrated mobile health intervention for secondary prevention of stroke on all-cause and cardiovascular mortality in rural China. The original intervention was tested in a cluster randomised controlled trial (SINEMA) which evaluated the effect of the intervention on blood pressure reduction.
While the study provides evidence for long term effect of this intervention on mortality, I have several concerns.
Major comments

*1.      One major concern is the way the data are presented. A key question is what has led to differences in mortality in the two arms after the follow up period. The answer to this question is not very clear in the data presented. One would like to know how the baseline characteristics varied in the follow up cohort in the two arms. The authors should provide the details of these characteristics for surviving individuals in the intervention and control arms in table. The data for the decedents should also be presented for both the arms separately.*

**Response:** Thank you for your valuable comment. Based on your comment, we have added two separate tables (**Table D and E in S2**) in the supplementary material to presents the baseline characteristics of surviving individuals and decedents for both arms separately.

For baseline characteristics of surviving participants, please refer to the text in the **Result section (pp 14)** as follows: “**Table D in S2 Text** presents the well-balanced baseline characteristics of surviving participants in two arms, except that surviving participants in the intervention arm had lower level of adherence to antihypertensive medications and slightly higher proportion in individuals suffer from moderate to severe disability.”

For baseline characteristics of deceased participants, we have added explanations in the **Result section (pp 15)** as follows: “**Table E in S2 Text** presents the baseline characteristics of decedents in two arms, which suggests higher proportion in females, being widowed, current smoker and hemorrghage stroke patients in the control arm than in the intervention arm at baseline among deceased participants.”

*2.      In addition to smoking tobacco, the authors should present data on other risk factors for stroke such as alcohol use, salt use or awareness about the harms of high salt use and BMI in both the arms during follow up if they have these data.*

**Response:** Thank you for your suggestion. We acknowledged the important of managing a series of risk factors for stroke patients. However, our study had decided to prioritize on medication use and physical activities in its design based on intensive needs assessment, and some of the risk factors you mentioned were not pre-specified and not available. Based on the best availability of the data, we have added the change of factors in BMI and waist circumference in **Table H in S2 Text** to provide more information to readers. We also acknowledge such limitation in the **Discussion section**, as follow: “Second, although we have tried to infer the roles of several factors through intermediate outcome analyses among survivors, we were unable to fully uncover the exact mechanisms by which the multicomponent intervention reduced mortality.”

*3.      The authors should provide a flow diagram of the number of patients enrolled, survived, died and migrated in both the arms during the initial trial period and subsequent to this period.*

**Response:** Thank you for your comment. We have moved the figure from supplement file to **Figure 1**.

*4.      The authors refer to the original paper for the details of the intervention but in the interest of the readers it will be important to describe these in brief. For example, the components of mobile health intervention and lifestyle interventions should be described in a few sentences in this manuscript as well.*

**Response:** Thank you for your valuable suggestion. We have added further details about the intervention in the section of **Interventions and implementation strategies** **section** to provide clarity for readers as follows: “Village doctors, who can prescribe essential medications [10], received 1.5-day of training, performance-based financial and non-financial incentives, and equipped with an Android smartphone with the SINEMA App installed to support the delivery of monthly follow-up and patients management [7,8]. Village doctors provided monthly follow-ups to stroke patients by following standardized procedures, including blood pressure monitoring, stroke symptom review, medication adherence checks, and therapeutic lifestyle recommendations. Patients also received daily voice messages with emphasis on physical activity and medication adherence, which were automatically dispatched every morning- recorded in the local dialect [7].”

*5.      Table 2 shows that in the cardiovascular mortality, it is the "non-stroke mortality" that is lower in the intervention arm. The authors might want to discuss potential reason for this as the intervention was primarily designed for stroke.*

**Response:** Thank you for your valuable suggestion. While the SINEMA intervention was primarily designed for stroke patients, several components of the intervention—such as blood pressure control, promotion of medication adherence (including statins and antiplatelet drugs), and increased physical activity—are also relevant for the prevention and management of other cardiovascular diseases (CVDs). We have added the relevant discussion in the **Discussion section (pp 19)**, as follows: “Interestingly, we also observed reduction in non-stroke cardiovascular mortality, indicating the potential broader CVD benefits beyond stroke prevention. Such observed reduction in non-stroke cardiovascular mortality may be attributable to the control of shared risk factors between stroke and other CVDs in blood pressure, improvement in medication adherence to statins and antiplatelet use and increase in physical activity; though more studies are needed to investigate such broader benefits. "

*6.      The authors have attributed the reduction is mortality primarily to medication adherence and modest reduction in blood pressure but have not considered effect of lifestyle changes. However, this is not very clear to me based on the data presented. This will only be true if the risk factor/lifestyle change distribution is not different in the two arms during the follow up period. By presenting the data as asked for in point 1 a reader may be able to infer better.*

**Response:** Thank you for your insightful comment. We agree that the reduction in mortality could be attributed to many factors and the long-term follow-up of this complex intervention is not able to fully reveal all the mechanism of change. We have added more presentation of lifestyle factors in available factors in **Table H in S2** **Text.**

Additionally, our intervention has been prioritize medication adherence in the nature of the intervention design, thus we believe that the maintenance in medication use and blood pressure control do play key roles. We have revised the statement in the **Discussion section** as follows: “The relative reduction in mortality observed in our study may seem larger than what can be expected from the reduction in blood pressure. Although the net reduction of 2.9 mm Hg in systolic blood pressure appeared modest, this reduction was sustained over the long term of six years. Although multiple factors and mechanisms could contribute to the changes in mortality and our complex intervention is not fully designed to illustrate such mechanisms of changes, this sustained effect likely resulted from the maintained behaviour changes in medication adherence in the intervention arm may provide some clues.”

Minor comments
*1.      Table 3, the number of patients included in the analysis is 979 but the number of survivors is 1023. The authors may want to provide an explanation on the footnote for this difference.*

**Response:** Thank you for your suggestion, we have added footnotes as follows: “At 70 months post-baseline, there were a total of 1,023 survivors, of whom 979 completed the long-term follow-up survey. The remaining 44 participants were either unreachable or declined to participate. The intermediate outcome analysis included only the 979 individuals who completed the survey.”

*2.      In table 3, the explanation for the data shown as percentages for medication adherence is not very clear. The authors have provided a note "‡ Medication adherence refers to a perfect adherence with score of 0 based on the 4-item Morisky Green Levine Scale." The wording may be changed to-"‡ Medication adherence refers to the percentage of patients with a perfect adherence which refers to a score of 0 on the 4-item Morisky Green Levine Scale."*

**Response:** Thank you for your suggestion. We have updated the explanation in Table 3 as follows: "‡ Medication adherence refers to the percentage of patients with a perfect adherence, which refers to a score of 0 on the 4-item Morisky Green Levine Scale."

-----------------------------------------------------------

***General editorial requests:***

**	We ask every co-author listed on the manuscript to fill in a contributing author statement, making sure to declare all competing interests. If any of the co-authors have not filled in the statement, we will remind them to do so when the paper is revised. If all statements are not completed in a timely fashion this could hold up the re-review process. If new competing interests are declared later in the revision process, this may also hold up the submission. Should there be a problem getting one of your co-authors to fill in a statement we will be in contact. Please do not add or remove authors without first discussing this with the handling editor.*

**Response:** We have reviewed and fully understand the journal’s policy regarding contributing author statements and competing interests.

**	Please upload any figures associated with your paper as individual TIF or EPS files with 300dpi resolution at resubmission. While revising your submission, please upload your figure files to the PACE digital diagnostic tool.*

**Response:** We have reviewed the figure guidelines and uploaded all relevant figures to the PACE digital diagnostic tool as requested.

**	Please ensure that the paper adheres to the PLOS Data Availability Policy, which requires that all data underlying the study's findings be provided in a repository or as Supporting Information. For data residing with a third party, authors are required to provide instructions with contact information (web or email address) for obtaining the data. Please note that a study author cannot be the contact person for the data. PLOS journals do not allow statements supported by "data not shown" or "unpublished results." For such statements, authors must provide supporting data or cite public sources that include it.*

**Response:** We have reviewed and adhered to the PLOS Data Availability Policy.

**	We expect all researchers with submissions to PLOS in which author-generated code underpins the findings in the manuscript to make all author-generated code available without restrictions upon publication of the work. In cases where code is central to the manuscript, we may require the code to be made available as a condition of publication. Authors are responsible for ensuring that the code is reusable and well documented.*

**Response:** The data used in this study are derived from real-world sources, and the study findings do not rely on author-generated code. The data code could be available by contacting to the responding author after approval.

**	ETHICS STATEMENTS: Please provide details of the consent and whether it was written or oral. Please also provide the approval numbers.*

**Response:** We have descripted the consent process and provide the approval numbers in the manuscript as follows: “This post-trial follow-up study was approved by the ethical boards of the Chinese Academy of Medical Sciences and Peking Union Medical College (approval number: CAMS&PUMC-IEC-2024-047) and Duke University (approval number: Pro00082130-AMD-9.1) and reported according to the Consolidated Standards of Reporting Trials (S3 CONSORT Checklist under extension for cRCT). All study participants or relatives of deceased participants provided written informed consent.”

**	COMPETING INTERESTS: All authors must declare their relevant competing interests per the PLOS policy.*

**Response:** All authors have read and adhered to the PLOS Competing Interests Policy and declare that they have no competing interests. The statement of no competing interests has also been included in the manuscript.

**	DATA AVAILABILITY: PLOS Medicine requires that the de-identified data underlying the specific results in a published article be made available, without restrictions on access, in a public repository or as Supporting Information at the time of article publication, provided it is legal and ethical to do so.*

**Response:** In accordance with local policies and the requirements of the ethics committees, the data used in this study will not be made publicly available. De-identified data may be accessed under appropriate conditions. The data-sharing statement has been included in the manuscript.

***FORMATTING - GENERAL***

**	Abstract: Please structure your abstract using the PLOS Medicine headings (Background, Methods and Findings, Conclusions). Please combine the Methods and Findings sections into one section.*

**Response:** The abstract has been structured according to the PLOS Medicine requirements.

**	At this stage, we ask that you include a short, non-technical Author Summary of your research to make findings accessible to a wide audience that includes both scientists and non-scientists. The Author Summary should immediately follow the Abstract in your revised manuscript. This text is subject to editorial change and should be distinct from the scientific abstract. Ideally each sub-heading should contain 2-3 single sentence, concise bullet points containing the most salient points from your study. In the final bullet point of 'What Do These Findings Mean?', please include the main limitations of the study in non-technical language.*

**Response:** The Author Summary has been added immediately following the Abstract, in accordance with the journal's requirements.

**	Please express the main results with 95% CIs as well as p values. When reporting p values please report as p<0.001 and where higher as the exact p value p=0.002, for example. Throughout, suggest reporting statistical information as follows to improve clarity for the reader "22% (95% CI [13%,28%]; p</=)". Please be sure to define all numerical values at first use.*

**Response:** We have formatted the p values and 95% CIs throughout the manuscript in accordance with the journal’s requirements.

**	Please include page numbers and line numbers in the manuscript file. Use continuous line numbers (do not restart the numbering on each page).*

**Response:** Page numbers and continuous line numbers have been added throughout the manuscript as requested.

**	Please cite the reference numbers in square brackets. Citations should precede punctuation.*

**Response:** All references have been inserted following the journal’s requirements, with reference numbers in square brackets and citations placed before punctuation.

***FIGURES AND TABLES***

**	Please provide titles and legends for all figures and tables (including those in Supporting Information files).*

**Response:** Titles and legends have been provided for all figures and tables, including those in the Supporting Information files.

**	Please define all abbreviations used in each figure/table (including those in Supporting Information files).*

**Response:** All abbreviations used in the figures and tables, including those in the Supporting Information files, have been defined in the notes.

**	Please consider avoiding the use of red and green in order to make your figure more accessible to those with color blindness.*

**Response:** No red or green colors have been used in any of the figures or tables.

***SUPPLEMENTARY MATERIAL***

**	Please note that supplementary material will be posted as supplied by the authors. Therefore, please amend it according to the relevant comments outlined here.*

**Response:** All requested revisions to the supplementary material, as outlined by the reviewers, have been addressed and updated in this revision.

**	Please cite your Supporting Information as outlined here:* [*https://urldefense.com/v3/__https://journals.plos.org/plosmedicine/s/supporting-information__;!!OToaGQ!ufDNprW_o3ijOSdWqEDR6L_kHKc6AqZOkPjtRyqykN_x5-fEa8a3SnhJTpLLWsT8c122byFD-BW_Vda60tU$*](https://urldefense.com/v3/__https://journals.plos.org/plosmedicine/s/supporting-information__;!!OToaGQ!ufDNprW_o3ijOSdWqEDR6L_kHKc6AqZOkPjtRyqykN_x5-fEa8a3SnhJTpLLWsT8c122byFD-BW_Vda60tU$)

**Response:** All citations of Supporting Information in the manuscript have been formatted according to the journal's guidelines.

***REFERENCES***

**	PLOS uses the numbered citation (citation-sequence) method and first six authors, et al.*

**Response:** All references have been formatted and cited according to the journal's guidelines, using the numbered citation method and listing the first six authors followed by "et al."

**	Please ensure that journal name abbreviations match those found in the National Center for Biotechnology Information (NCBI) databases and are appropriately formatted and capitalised.*

**Response:** All journal name abbreviations have been checked and formatted to match the standards in the NCBI database.

**	Where website addresses are cited, please include the complete URL and specify the date of access (e.g. [accessed: 12/06/2023]).*

**Response:** No website addresses have been cited in this manuscript.

**	Please also see https://urldefense.com/v3/__https://journals.plos.org/plosmedicine/s/submission-guidelines*loc-references__;Iw!!OToaGQ!ufDNprW_o3ijOSdWqEDR6L_kHKc6AqZOkPjtRyqykN_x5-fEa8a3SnhJTpLLWsT8c122byFD-BW_pwI1FyU$ for further details on reference formatting.*

**Response:** We have reviewed the reference formatting guidelines provided by the journal and ensured that all references adhere to these requirements.

***STUDY TYPE-SPECIFIC REQUESTS***

**	Please be explicit in the title and the abstract that is a post-hoc sub-study/follow-on study of the primary trial. We suggest reporting in-line with CONSORT explicitly stating the sub-study nature and ensuring that the abstract details the main trial items in 2-3 sentences, including the study population, dates, intervention and primary outcome. The majority of the abstract should then describe the complete details of this post-hoc sub-study.*

**Response:** The study has been reported in accordance with the CONSORT guidelines and the journal’s requirements, with all relevant sections explicitly addressing the nature of this study.

**	Please complete the CONSORT checklist and ensure that all components of CONSORT are present in the manuscript as well as clearly defined details of this sub-study. When completing the checklist, please use section and paragraph numbers, rather than page numbers as these often change in the event of publication.*

**Response:** The CONSORT checklist has been submitted as Supporting Information. Section and paragraph numbers have been used in the checklist as requested.

**	Please ensure that study registration details are included in the Methods section.*

**Response:** The study registration details have been provided in the Methods section of the manuscript.

**	Abstract: Please include the study design, population and setting, number of participants, years during which the study took place (original enrollment and follow up), length of follow up, and main outcome measures.*

**Response:** The abstract has been updated to include all required information as mentioned above.

**	Please include absolute numbers wherever you report percentages; eg, n/N (%)*

**Response:** Absolute numbers have been provided wherever percentages are reported in the manuscript.

**	In keeping with our commitment to Open Science, please include the study protocol document and analysis plan (including any amendments) as Supporting Information to be published with the manuscript if accepted.*

**Response:** We agree to make the study protocol publicly available, and it has already been submitted as Supporting Information during this submission.
