# Supplementary material for: Long-term mortality outcome of a primary care-based mobile health intervention for stroke management: Six-year follow-up of a cluster-randomized controlled trial
Source: PLoS Med. 2025 Mar 27;22(3):e1004564. doi: 10.1371/journal.pmed.1004564 (PMC11949329; doi:10.1371/journal.pmed.1004564)
Supplement: Attachment — Submitted filename: Response letter R2-PLOS Med-clean.docx [file pmed.1004564.s005.docx]

Alexandra Tosun, PhD
Associate Editor - PLOS Medicine
Manuscript no.: PMEDICINE-D-24-03550R2

Feb 3, 2025

Dear Dr. Tosun,

Thank you for your and the reviewers’ valuable comments and suggestions on our manuscript entitled “**Long-term effect on mortality of a primary care-based mobile health intervention for stroke: six-year follow-up of a cluster-randomized controlled trial.**” We appreciate the opportunity to further revise and improve our manuscript based on the previous round of revisions and have carefully addressed the concerns raised by you and the reviewers. Please find our item-by-item responses to each comment raised by you and the reviewers. We have also submitted a clean version of the revised manuscript. We believe that the manuscript has been significantly improved and sincerely hope it will now meet the standards for publication in ***PLOS Medicine***.

Lijing L. Yan, PhD, MPH, and Enying Gong, PhD, *on behalf of all authors*

-----------------------------------------------------------

**Comments from Reviewers:**

Reviewer #1: Thank you to the authors for addressing my previous comments well. I have no further issues to raise.

**Response:** We appreciate your positive comments.

Reviewer #3: The authors have not provided the demographic features of the participants in the cohort for this follow up study. The cohort for this follow up study has 626 survivors in the intervention arm and 643 in the control arm as shown in Figure 1. I have raised this concern in the earlier review as well. This is very important as these individuals form the baseline for the follow up phase described in this manuscript. They should present this data in table 1. The data on survivors and decedents in table 1 can be moved to supplementary material. The authors have addressed my other concerns.

**Response:** Thank you for your suggestion. We have followed your suggestions to provide the baseline characteristics of all participants (n=1299) for the SINEMA trial and participants who were survived at 1-year (n=1269) for long-term follow-up (**Table 1**). The data on survivors and decedents have been moved to the supplementary materials (**Table F in S2 Supporting information**).

-----------------------------------------------------------

**Requests from Editors:**

**GENERAL**
1) Your study is observational and therefore causality cannot be inferred. Please remove any language that implies causality, such as effect. Please refer to associations instead. Please revise throughout the entire manuscript including figures and tables.
**Response:** Thank you for your insightful comment, though we also identified other studies that implies the long-term real-world effectiveness from observational follow-up of trial participants. We do consider our study as a long-term observational study followed participants of a cluster RCT to measure the long-term sustained real-world effectiveness on mortality. We acknowledged the potential influence of unmeasured factors that may influence the causal inferences and we have expanded such interpretation in the discussion section (See response to your question 2). We revised the relevant statement throughout the entire manuscript including Tables and Figures to avoid potential overstatement on causality.

2) Given that the intervention was not continued after the 12-month intervention period, we ask you to temper the findings and conclusions of your study. Within the 58-month time frame following the active trial, other factors not accounted for may have influenced the associations observed in this study, i.e., the sustained link you observe may not be a result of the intervention. Accordingly, we ask you to expand the limitations section to consider factors such as the survey-based nature of your study and the confounders that may not have been accounted for.

**Response:** Thank you for your suggestion. We have expanded the limitations section to address these concerns. Please see the revision in discussion **on p 21**, **line 491-495**: “Moreover, given the observational nature of the post-trial follow-up phase and the potential influence of unobserved interventions or unmeasured factors on mortality, we are not able to fully build the causal inferences and caution should be exercised when interpreting the findings.”

3) The terms gender and sex are not interchangeable (as discussed in https://www.who.int/health-topics/gender#tab=tab_1 ); please use the appropriate term and revise accordingly throughout the manuscript. Please note that you currently use both.

**Response:** Thank you, we have carefully reviewed the manuscript and ensured that 'sex' is used consistently throughout the text, figures, tables, and supplementary materials.

**TITLE**

Please revise your title according to PLOS Medicine's style. Your title must be nondeclarative and not a question. It should begin with main concept if possible. "Effect of" should be used only if causality can be inferred, i.e., for an RCT. Please place the study design ("A randomized controlled trial," "A retrospective study," "A modelling study," etc.) in the subtitle (ie, after a colon).

**Response:** Thank you. We have revised the title to align with PLOS Medicine’s style. The updated title is as follows: Long-term mortality outcome of a primary care-based mobile health intervention for stroke management: six-year follow-up of a cluster-randomized controlled trial

**ETHICS**

The ethical approval numbers from the ethical boards of the Chinese Academy of Medical Sciences between the protocol (CAMS & PUMC-IEC-2022-062) and the manuscript (CAMS&PUMC-IEC-2024-047) do not match. Please check and revise.

**Response:** Thank you for pointing that out. CAMS &PUMC review board provide a new approval number once it completed routine renewing and review. We have now kept both IRB numbers in the manuscript to in line with the IRB approval status of the study.

**DATA AVAILABILITY**

Thank you for providing a data availability statement. We feel that other researchers would not know how to request the data from the links you provide. Could you please provide a web or email address that provides a clearer way to access the data?

**Response:** We have updated the data sharing statement to include the email of the corresponding authors regarding any inquiries for data usage or code usage. The details can be found in the manuscript **on page 23, in the Data sharing section.**

**CODE AVAILABILITY**

You have stated that the data code could be made available by contacting the responding author after approval. Could you please include this statement in your data availability statement?

**Response:** We have included the statement regarding code availability in the Data Sharing section.

**ABSTRACT**

1) Please clarify in the Abstract that the study is part of the Stroke Patients and Family Longitudinal Study in Rural China (SaFaRI, NCT05792618), a long-term passive (observational) follow-up of SINEMA cRCT participants and their spouses, as stated in your rebuttal. It should be clear to the reader that this is an observational study and not a pre-specified long-term outcome of the SINEMA trial.

**Response:** We have added the statement in the beginning of the methods and findings section as follows on **page 3, lines 58-60**: This study (clinicltiral.gov registration number: NCT05792618) is a long-term passive observational follow-up of participants and their spouse of the SINEMA cluster randomized controlled trial (clinicaltrial.gov registration number: NCT03185858).

2) l.72ff: Throughout the abstract and main text, please ensure that abbreviations, including statistical abbreviations, are defined the first time they are used (e.g. Q, CI etc.).

**Response:** Thank you for your comment. We have ensured that all abbreviations, including statistical abbreviations, are defined the first time they are used throughout the abstract.

3) l.76/l.77: We suggest changing ‘cardiovascular mortality’ and ‘stroke mortality’ to ‘cardiovascular cause-specific mortality’ and ‘stroke cause-specific mortality’ (or similar). Please revise throughout the main text.

**Response:** Thank you for your suggestion. We have replaced 'cardiovascular mortality' and 'stroke mortality' with 'cardiovascular cause-specific mortality' and 'stroke cause-specific mortality' throughout the main text and supplementary materials.

4) Since the primary objective of the SINEMA trial was to determine whether an integrated primary care-based mobile health intervention could improve stroke management, we feel that you should emphasize in the Abstract Conclusion that during six years of follow-up, no difference in stroke-related mortality was observed between the intervention group and the usual care group.

**Response:** We have updated the Conclusion of the abstract as follows: Despite no observed long-term effect on stroke cause-specific mortality, the 12-month SINEMA intervention, compared with usual care, significantly associated with reduced all-cause and cardiovascular cause-specific mortality during six years of follow-up, suggesting potential sustained long-term benefits to stroke patients.

5) In the last sentence of the Abstract Methods and Findings section, please describe the main limitation(s) of the study's methodology.

**Response:** We have added the following main limitation to the Abstract Methods and Findings section: Although multisource verification were used to verify the outcomes, limitations exist as the survey- and record-matching based nature of the study, unavailability of accurate clinical diagnostic records for some cases and the potential confounders that may influence the observed association on mortality.

6) Please ensure that all numbers presented in the abstract are present and identical to numbers presented in the main manuscript text.

**Response:** We have reviewed the abstract and cross-checked all numbers with the main manuscript text and have confirmed the consistency.

7) Please include the important dependent variables that are adjusted for in the analyses.

**Response:** We have added the primary statistical model and the important dependent variables adjusted for in the analyses in the Methods and findings section as follows on **p 3, lines 70-72**: Cox proportional hazards models with cluster-robust standard errors were used to compute hazard ratios (HRs) and 95% confidence intervals (95% CIs), adjusting for town, age, and sex in the main analysis model.

8) Since you have included the ClinicalTrials.gov IDs below the Abstract, we suggest deleting the sentence on lines 68-70.

**Response:** We have included the ClinicalTrials.gov IDs for the relevant studies and have removed the sentence on lines 68-70 accordingly.

9) l.61: Please spell out ‘mHealth’ the first time used.

**Response:** We have spelled out ‘mHealth’ in the Abstract as requested.

**AUTHOR SUMMARY**

1) l.110: Please remove ‘stroke mortality’ since the difference was not significant.

**Response:** We have removed 'stroke mortality' from the Author summary.

2) The last bullet point under ‘What did the researchers do and find?’ should be moved under last sub-heading.

**Response:** We have moved the last bullet point under 'What did the researchers do and find?' to the last sub-heading as requested.

3) We suggest changing the third bullet point under ‘What did the researchers do and find?’ to: “Stronger associations were observed among more vulnerable individuals, which may indicate the inequity-reducing impact of the intervention.”

**Response:** We have revised the point accordingly.

4) We feel that the bullet points under the heading "What do these findings mean?" could be more concise. Please revise. Ideally, the subheading should contain 2-3 single sentence, concise bullet points that summarize the most important points of your study.

**Response:** Thank you for your suggestion. We have merged and revised several of the bullet points to make them more concise.

**INTRODUCTION**

If there has been a systematic review of the evidence related to your study (or you have conducted one), please refer to and reference that review and indicate whether it supports the need for your study.

**Response:** Before drafting the manuscript, a systematic literature search was conducted, while no relevant systematic reviews have been published in the field. We have mentioned all relevant trials that we have identified via the systematic search in the introduction to describe the evidence-base.

**METHODS AND RESULTS**

1) Similar to comment 1 under "Abstract", please clarify that the manuscript describes the Stroke Patients and Family Longitudinal Study in Rural China (SaFaRI, NCT05792618), a long-term passive (observational) follow-up of SINEMA cRCT participants and their spouses.

**Response:** We have made clarification in the first sentence of the **Methods section**.

2) l.168ff: “stroke survivors” - PLOS Medicine prefers the use of patient-centered language, e.g. “patients who were clinically stable after stroke” (or similar). Please revise throughout the manuscript.

**Response:** We have replaced "stroke survivors" with "patients who were clinical stable after stroke” or participants throughout the manuscript.

3) l. 216: Please define ‘ID’ at first use.

**Response:** Thank you and we have spelled out in full for “ID” at its first occurrence in the manuscript.

4) ll.228-231, please change to: “For six decedents with missing dates of death (2% of confirmed deaths), dates were imputed using median survival times, which were 8.69 months for five individuals who died during the one-year trial phase and 46.39 months for one individual who died during the post-trial phase.”

**Response:** Thank you for your suggestion. We have made the revision accordingly on **p 11, lines 233-236**.

5) l.277ff: Please ensure to introduce abbreviations the first time used (here: ‘HR’, introduced on line 281, but should be introduced on line 277).

**Response:** Thank you for pointing this out. We have revised it to ensure that the full term is provided, and the abbreviation is introduced at its first occurrence.

6) l.283: “…while the fully adjusted model included these variables along with additional baseline variables noted to be significantly different between the arms at baseline (P<0.05) [16].” – we do not feel that providing a reference is sufficient. Please outline the variable adjusted for in the fully adjusted model or detail in the Appendix.

**Response:** Thank you for your suggestion. We have explicitly listed these variables in the manuscript and revised the sentence accordingly. You can find the details on **p 13, lines 285-290.**

7) l.289: The terms gender and sex are not interchangeable (as discussed in https://www.who.int/health-topics/gender#tab=tab_1 ); please use the appropriate term.

**Response:** We have consistently used sex throughout the manuscript.

8) l.335: The Table E title is ‘Baseline characteristics of decedents in the SINEMA trial by intervention arms at 70-months post-baseline’ – it is our understanding that you are presenting characteristics at 70 months post baseline, so the first "baseline" should be removed. Please revise the table title and text accordingly.

**Response:** Table E did present the baseline characteristics of participants who died during the long-term follow-up. We have revised the title to avoid misunderstanding.

9) Figure 2: Please note that each figure should be self-explanatory on its own, i.e. we ask you to include a very brief description of what the intervention entailed in the figure description.

**Response:** Thank you for your suggestion. We have added a brief description of the intervention below the figure title, as follows: Light purple area indicates 12-month active intervention phase. Light yellow area indicates post-trial observational phase. (C) indicates control arm receiving usual care (blue lines) and (I) indicates arm receiving the SINEMA intervention, a primary care-based mobile health intervention for stroke secondary prevention (orange lines).

10) l.349ff: We feel that you should explain the results for stroke-specific mortality in more detail, because compared to the results for all-cause and cardiovascular-specific mortality, the results were not significant, i.e. there was no evidence of an association.

**Response:** We have separated the descriptions of all-cause and cardiovascular cause-specific mortality from stroke cause-specific mortality, as follows **on p 16, lines 360-363**:“Although lower cumulative incidence was observed in the intervention arm (6.0%) compared with the control arm(7.9%), for stroke cause-specific mortality, such differences was not statistically significant (HR was 0.71 (95% CI [0.44, 1.15]; P=0.16).

11) l.357: The reference to Table F appears to be incorrect. The results for the sub-distribution model are presented in Table G. Please carefully check that you have referenced the appropriate tables and figures throughout your manuscript and revise accordingly.

**Response:** Thank you for pointing this out. We have revised the appendix and confirmed that all references to supplementary tables are now correct.

12) l.359: What about age, stroke duration, baseline systolic blood pressure and stroke type?

**Response:** Thank you for raising this point. We now have interpreted the findings for all identified significant between-arm differences for subgroups. Please refer to the following revision on **p 16, lines 369-373:** “The subgroup analysis on all-cause mortality showed that greater between-arm differences were observed with stronger association among participants who were female, younger than 65 years old, less educated, had lower household incomes, were divorced or widowed, had shorter stroke duration, had systolic blood pressure lower than 140 mmHg or had more severe disabilities at baseline (**Figure 3**)”

13) l.374: The term trend should be used only when the test for trend has been conducted. Please revise accordingly.

**Response:** Thank you, we have replaced "Trends" with "Observed differences" accordingly.

14) l.378: Please define ‘BMI’.

**Response:** We have defined "BMI" in the manuscript.

15) l.379: As noted in our comment 10), please carefully check that you have provided the relevant tables and have referred to the appropriate tables and figures throughout your manuscript. Table H does not show the results of the post-hoc analysis on changes in potential lifestyle factors.

**Response:** Thank you, we have updated the tables in the appendix and ensured that all references to supplementary tables and figures are now correct.

16) Figure 1: Please define 'SD'. Please revise for patient-centered language.

**Response:** We have updated Figure 1 accordingly, including defining "SD" and ensuring patient-centered language.

17) Table 1: Please see the comment about the terms gender and sex and revise accordingly.

**Response:** We have consistently used "sex" throughout the manuscript.

18) Tables: Please revise the reference callouts below the table to meet our formatting requirements.

**Response:** We have revised the citation formatting in the table notes to comply with PLOS Medicine’s style requirements.

**DISCUSSION**

General guidance: Please present and organize the Discussion as follows: a short, clear summary of the article's findings; what the study adds to existing research and where and why the results may differ from previous research; strengths and limitations of the study; implications and next steps for research, clinical practice, and/or public policy; one-paragraph conclusion.

**Response:** We have revised the discussion section to improve its flow and logic according to PLOS Medicine's requirements. We hope such revision increase the readability of the discussion section.

1) l.388: “Our study is unique among the few cRCTs in LMICs…” – please clarify that the current study is of observational nature and a long-term follow-up of a RCT.

**Response:** Thank you for your comment. As we mentioned in our response to the Editor’s first comment, our current analysis is based on both trial and post-trial follow-up data, so describing it as purely observational would be incomplete. However, we partially acknowledge the Editor’s point and have revised the sentence as follows: Our study, one of the few cRCTs on stroke patients in LMICs, also uniquely reports the sustained effects of a time-limited intensive intervention through long-term follow-up after the trial [2-4].

2) l.448: Please define ‘CVD’ at first use.

**Response:** We have revised the manuscript to ensure that "CVD" appears in full as “cardiovascular disease”.

3) Please remove any subheadings including the Conclusion subheading.

**Response:** We have removed the the "Conclusion" subheading from the manuscript.

**REFERENCES**

1) Please ensure that journal name abbreviations match those found in the National Center for Biotechnology Information (NCBI) databases (http://www.ncbi.nlm.nih.gov/nlmcatalog/journals), and are appropriately formatted and capitalized. For example, in reference [1], ‘The Lancet

Neurology’ should be ‘Lancet Neurol’.

**Response:** We have updated all journal names in the references according to the Title Abbreviation format in the NCBI database.

2) Please also see https://journals.plos.org/plosmedicine/s/submission-guidelines#loc-references for further details on reference formatting.

**Response:** We have reviewed the reference formatting guidelines again and confirmed that the references comply with PLOS Medicine’s style requirements.

**SUPPLEMENTARY MATERIAL**

1) Please ensure that all supplementary files are referenced in the main text.

**Response:** We have reviewed the manuscript and supplementary files and confirmed that all supplementary materials are properly referenced in the main text.

2) In the published article, supporting information files are accessed only through a hyperlink attached to the captions. For this reason, you must list captions at the end of your manuscript file. You may include a caption within the supporting information file itself, as long as that caption is also provided in the manuscript file. Do not submit a separate caption file.

When SI files are contained with a single file:

Please label the file as ‘S1 Supporting Information’.

Please apply alphabetical labelling to each table and figure contained within the S1 file. For example, ‘Fig A’ to ‘Fig Z’ and ‘Table A’ to ‘Table Z’.

Plain text does not need to be labelled and can just be given a title as necessary. For example, ‘Statistical Analysis Plan’.

Please cite tables/figures as ‘Fig A in S1 Supporting Information’ and/or ‘Table A in S1 Supporting Information’, for example.

Please cite plain text as, ‘Statistical Analysis Plan in S1 Supporting Information’, for example.

When SI files are uploaded as separate files:

Please label tables as ‘S1 Table’ (so on) and figures as ‘S1 Fig’ (and so on).

Any additional documents (protocols/analysis plans etc.) can be labelled as ‘S1 Protocol’, for example. Please cite items as exactly as labelled.

**Response:** We have added a “Supporting information” subheading at the end of the manuscript. Under this subheading, we have labelled all supplementary materials in accordance with the provided instructions. You may find them on pages 40–41 of the manuscript.

-----------------------------------------------------------

***General editorial requests:***

*1) We ask every co-author listed on the manuscript to fill in a contributing author statement. If any of the co-authors have not filled in the statement, we will remind them to do so when the paper is revised. If all statements are not completed in a timely fashion this could hold up the re-review process. Should there be a problem getting one of your co-authors to fill in a statement we will be in contact. YOU MUST NOT ADD OR REMOVE AUTHORS UNLESS YOU HAVE ALERTED THE EDITOR HANDLING THE MANUSCRIPT TO THE CHANGE AND THEY SPECIFICALLY HAVE AGREED TO IT.*

**Response:** We understand the journal’s policies. If any issues concerning authors arise, please feel free to contact us.

*2) Please ensure that the paper adheres to the PLOS Data Availability Policy (see http://journals.plos.org/plosmedicine/s/data-availability), which requires that all data underlying the study's findings be provided in a repository or as Supporting Information. For data residing with a third party, authors are required to provide instructions with contact information for obtaining the data. PLOS journals do not allow statements supported by "data not shown" or "unpublished results." For such statements, authors must provide supporting data or cite public sources that include it.*

**Response:** We have reviewed and adhered to the PLOS Medicine Data Availability Policy. In this revision, we have provided a detailed email address for the inquiry regarding data and code sharing.

*3) Please review your reference list to ensure that it is complete and correct. If you have cited papers that have been retracted, please include the rationale for doing so in the manuscript text, or remove these references and replace them with relevant current references. Any changes to the reference list should be mentioned in the rebuttal letter that accompanies your revised manuscript.*

**Response:** In this revision, we have not added or removed any references. However, following the editor's requirements, we have reformatted the journal names in the reference list to align with the NLM Title Abbreviation format.
